# Serological response and breakthrough infection after COVID-19 vaccination in patients with cirrhosis and post-liver transplant

**DOI:** 10.1097/HC9.0000000000000273

**Published:** 2023-10-18

**Authors:** Gautam Mehta, Antonio Riva, Maria Pilar Ballester, Eva Uson, Montserrat Pujadas, Ângela Carvalho-Gomes, Ivan Sahuco, Ariadna Bono, Federico D’Amico, Raffaela Viganò, Elena Diago, Beatriz Tormo Lanseros, Elvira Inglese, Dani Martinez Vazquez, Rajni Sharma, Hio Lam Phoebe Tsou, Nicola Harris, Annelotte Broekhoven, Marjolein Kikkert, Shessy P. Torres Morales, Sebenzile K. Myeni, Mar Riveiro-Barciela, Adriana Palom, Nicola Zeni, Alessandra Brocca, Annarosa Cussigh, Sara Cmet, Desamparados Escudero-García, Matteo Stocco, Leonardo Antonio Natola, Donatella Ieluzzi, Veronica Paon, Angelo Sangiovanni, Elisa Farina, Clara di Benedetto, Yolanda Sánchez-Torrijos, Ana Lucena-Varela, Eva Román, Elisabet Sánchez, Rubén Sánchez-Aldehuelo, Julia López-Cardona, Itzel Canas-Perez, Christine Eastgate, Dhaarica Jeyanesan, Alejandro Esquivel Morocho, Simone Di Cola, Lucia Lapenna, Giacomo Zaccherini, Deborah Bongiovanni, Paola Zanaga, Katia Sayaf, Sabir Hossain, Javier Crespo, Mercedes Robles-Díaz, Antonio Madejón, Helena Degroote, Javier Fernández, Marko Korenjak, Xavier Verhelst, Javier García-Samaniego, Raúl J. Andrade, Paula Iruzubieta, Gavin Wright, Paolo Caraceni, Manuela Merli, Vishal C Patel, Amir Gander, Agustín Albillos, Germán Soriano, Maria Francesca Donato, David Sacerdoti, Pierluigi Toniutto, Maria Buti, Christophe Duvoux, Paolo Antonio Grossi, Thomas Berg, Wojciech G. Polak, Massimo Puoti, Anna Bosch-Comas, Luca Belli, Patrizia Burra, Francesco Paolo Russo, Minneke Coenraad, José Luis Calleja, Giovanni Perricone, Marina Berenguer, Joan Claria, Richard Moreau, Vicente Arroyo, Paolo Angeli, Cristina Sánchez, Javier Ampuero, Salvatore Piano, Shilpa Chokshi, Rajiv Jalan

**Affiliations:** 1 Institute for Liver and Digestive Heath, University College London, London, UK; 2 The Roger Williams Institute of Hepatology, Foundation for Liver Research, London, UK; 3 Royal Free London NHS Foundation Trust, London, UK; 4 Faculty of Life Sciences and Medicine, King’s College London, London, UK; 5 Department of Gastroenterology and Hepatology, Hospital Clínico Universitario de Valencia, Valencia, Spain; 6 European Foundation for the Study of Chronic Liver Failure (EF CLIF), EASL-CLIF Consortium and Grifols Chair, Barcelona, Spain; 7 Hepatology, HBP Surgery and Transplantation, Hepatology & Liver Transplant Unit, La Fe University Hospital, Valencia, Spain; 8 Ciberehd, Universidad de Valencia, Valencia, Spain; 9 ASST Grande Ospedale Metropolitano Niguarda, Infectious Diseases Unit, Milan, Italy; 10 Department of Medical Biotechnology and Translational Medicine, Postgraduate School of Clinical Pharmacology and Toxicology, University of Milan, Milan, Italy; 11 ASST Grande Ospedale Metropolitano Niguarda, Hepatology and Gastroenterology Unit, Milan, Italy; 12 Department of Gastroenterology and Hepatology, Hospital Universitario Puerta de Hierro Majadahonda, IDIPHIM, Madrid, Spain; 13 Central Unit of Clinical Research and Clinical Trials, Hospital Universitario La Paz, IdiPaz, Madrid, Spain; 14 CIBERehd, Madrid, Spain; 15 Department of Brain and Behavioral Sciences, University of Pavia, Pavia, Italy; 16 Liver Unit, Hospital Universitario Valle de Hebron, Barcelona, Spain; 17 Department of Gastroenterology and Hepatology, Leiden University Medical Center, RC Leiden, the Netherlands; 18 Department of Medical Microbiology, Leiden University Medical Center, RC Leiden, the Netherlands; 19 Department of Medicine - DIMED, Unit of Internal Medicine and Hepatology (UIMH), University of Padova, Padova, Italy; 20 Hepatology and Liver Transplantation Unit, Azienda Sanitaria Universitaria Integrata, University of Udine, Udine, Italy; 21 Azienda Ospedaiera Universitaria Integrata Verona, Verona Italy; 22 Foundation IRCCS Ca’ Granda Ospedale Maggiore Policlinico, Division of Gastroenterology and Hepatology, Milan, Italy; 23 Hospital Universitario Virgen del Rocio, Sevilla. Instituto de Biomedicina de Sevilla, Universidad de Sevilla, Sevilla, Spain; 24 Hospital de la Santa Creu i Sant Pau, Barcelona, Spain; 25 EUI-Sant Pau School of Nursing, Barcelona, Spain; 26 Servicio de Gastroenterología, Hospital Universitario Ramón y Cajal, Instituto Ramón y Cajal de Investigación Sanitaria (IRYCIS), Centro de Investigación Biomédica en Red de Enfermedades Hepáticas y Digestivas (CIBEREHD), Instituto Salud Carlos III, Madrid, Spain; 27 Universidad de Alcalá, Madrid, Spain; 28 Institute of Liver Studies, King’s College Hospital NHS Foundation Trust, London, UK; 29 Department of Translational and Precision Medicine, University of Rome Sapienza, Roma, Italy; 30 Department of Medical and Surgical Sciences, University of Bologna, Bologna, Italy; 31 Department of Surgery, Oncology and Gastroenterology, University of Padova, Padova, Italy; 32 Gastroenterology and Multivisceral Transplant Units, Azienda Ospedale Università’ di Padova, Padova, Italy; 33 Mid & South Essex NHS Foundation Trust, Basildon, UK; 34 Gastroenterology and Hepatology Department, Marqués de Valdecilla University Hospital, Santander, Spain; 35 Clinical and Traslational Digestive Research Group, Instituto de Investigación Sanitaria Valdecilla (IDIVAL), Santander, Spain; 36 Servicio de Aparato Digestivo, Hospital Universitario Virgen de la Victoria, Universidad de Málaga, Málaga, Spain; 37 Liver Unit, Hospital Universitario La Paz, CIBERehd, IdiPAZ, Universidad Autónoma de Madrid, Madrid, Spain; 38 Department of Gastroenterology and Hepatology, Ghent University Hospital, Belgium; 39 Liver Research Center Ghent, Ghent University, Belgium; 40 European Reference Network (ERN)RARE-LIVER; 41 Liver Unit, Hospital Clínic, Universitat de Barcelona, Institut d’Investigacions Biomèdiques August Pi-Sunyer (IDIBAPS) and Centro de Investigación Biomèdica en Red (CIBEREHD), Barcelona, Spain; 42 European Liver Patients’ Association (ELPA); 43 Unit of Semeiotics, Liver and Alcohol-related Diseases, Azienda Ospedaliero-Universitaria di Bologna, Bologna, Italy; 44 Department of Hepatogy-Liver Transplant Unit, Henri Mondor Hospital-APHP, Paris Est University, Paris, France; 45 Department of Medicine and Surgery, University of Insubria, Infectious and Tropical Diseases Unit, ASST Sette Laghim, Varese, Italy; 46 European Association for the Study of the Liver (EASL); 47 Department of Surgery, Division of HPB and Transplant Surgery, Erasmus MC Transplant Institute, University Medical Center Rotterdam, Rotterdam, the Netherlands; 48 University of Milano Bicocca, Infectious Diseases Niguarda Great Metropolitan Hospital, Milan, Italy; 49 Hospital Clínic, Institut d’Investigacions Biomèdiques August Pi-Sunyer (IDIBAPS), Centro de Investigación Biomédica en Red (CIBERehd) and Universitat de Barcelona, Barcelona, Spain; 50 INSERM and Université Paris Cité, Centre de Recherche sur l’inflammation (CRI), Paris, France; 51 APHP, Service d’hépatologie, Hôpital Beaujon, Clichy, France

## Abstract

**Background:**

Vaccine hesitancy and lack of access remain major issues in disseminating COVID-19 vaccination to liver patients globally. Factors predicting poor response to vaccination and risk of breakthrough infection are important data to target booster vaccine programs. The primary aim of the current study was to measure humoral responses to 2 doses of COVID-19 vaccine. Secondary aims included the determination of factors predicting breakthrough infection.

**Methods:**

COVID-19 vaccination and Biomarkers in cirrhosis And post-Liver Transplantation is a prospective, multicenter, observational case-control study. Participants were recruited at 4–10 weeks following first and second vaccine doses in cirrhosis [n = 325; 94% messenger RNA (mRNA) and 6% viral vaccine], autoimmune liver disease (AILD) (n = 120; 77% mRNA and 23% viral vaccine), post-liver transplant (LT) (n = 146; 96% mRNA and 3% viral vaccine), and healthy controls (n = 51; 72% mRNA, 24% viral and 4% heterologous combination). Serological end points were measured, and data regarding breakthrough SARS-CoV-2 infection were collected.

**Results:**

After adjusting by age, sex, and time of sample collection, anti-Spike IgG levels were the lowest in post-LT patients compared to cirrhosis (*p* < 0.0001), AILD (*p* < 0.0001), and control (*p* = 0.002). Factors predicting reduced responses included older age, Child-Turcotte-Pugh B/C, and elevated IL-6 in cirrhosis; non-mRNA vaccine in AILD; and coronary artery disease, use of mycophenolate and dysregulated B-call activating factor, and lymphotoxin-α levels in LT. Incident infection occurred in 6.6%, 10.6%, 7.4%, and 15.6% of cirrhosis, AILD, post-LT, and control, respectively. The only independent factor predicting infection in cirrhosis was low albumin level.

**Conclusions:**

LT patients present the lowest response to the SARS-CoV-2 vaccine. In cirrhosis, the reduced response is associated with older age, stage of liver disease and systemic inflammation, and breakthrough infection with low albumin level.

## INTRODUCTION

Vaccination against SARS-CoV-2 has been remarkably successful in reducing infections, hospitalizations, and deaths from COVID-19 and has reopened much of society.^1^ However, vaccine acceptance has declined since the onset of the pandemic, and less than 70% of the global population have completed initial COVID-19 vaccination, with this percentage dropping to 35% in Africa.^2^ Among liver patients and solid organ transplant recipients, vaccine hesitancy remains an issue, demonstrating that targeted vaccination policy remains an important topic for the global hepatology community.^3,4^

Regarding immunogenicity of COVID-19 vaccination, data have emerged over recent months suggesting decreased immunogenicity in post-liver transplant (LT) patients largely associated with the use of immunosuppressive drugs and age [recently reviewed in Luo et al^5^. By contrast, the situation for patients with chronic liver disease (CLD)remains unclear, with major implications for guidance on vaccine booster regimens. Varying degrees of vaccine response in patients with cirrhosis have been reported in different studies, from comparable responses to healthy controls to markedly reduced responses. A summary of these data evaluating immunogenicity after 2 doses of COVID-19 vaccination in patients with CLD is presented in Table 1.

**TABLE 1:** Summary of the most relevant studies evaluating immunogenicity after complete COVID-19 vaccination in patients with chronic liver disease

References	Main end point	Patients included	Main results
Wang J, et al. J Hepatol^6^	Neutralizing antibody response at least 14 d after 2 doses of inactivated vaccine	381 patients with NAFLD, without a history of SARS-CoV-2 infection	Neutralizing antibodies were detected in 95.5% of patients
Thuluvath PJ, et al. J Hepatol^7^	Spike protein 4 wk after the second dose of mRNA vaccines or after the single dose of Johnson & Johnson vaccine	62 LT recipients, 79 patients with cirrhosis (10 decompensated), and 92 CLD without cirrhosis	Poor antibody responses were seen in 61% of LT patients and 24% of those with CLD
Ruether DF, et al. Clin Gastroenterol Hepatol^8^	Spike-protein titers and T-cell responses before and 10 to 84 d after second vaccination	194 patients (141 LT, 53 cirrhosis Child-Turcotte-Pugh A–C) and 56 controls	Seroconversion was achieved in 63% of LT and 100% of patients with cirrhosis and controls. Spike-specific T-cell response rates were 36.6%, 65.4%, and 100% in LT, cirrhosis, and controls
Willuweit K, et al. Vaccines (Basel)^9^	Immunogenicity after receiving 2 doses of the mRNA-based vaccine BNT162b2	110 patients with cirrhosis and 80 healthcare workers	96% and 99% of patients with cirrhosis and healthy controls developed antibodies, but the median IgG titer was significantly lower in patients with cirrhosis
Bakasis AD, et al. Viruses^10^	S-spike IgG antibodies and neutralizing activity in fully vaccinated patients and controls	87 patients with liver diseases and 40 controls	Seroconversion rates were 97.4%, 87.8%, and 100%, and adequate neutralizing activity was detected in 92.1%, 87.8%, and 100% of patients with cirrhosis, non-cirrhotics, and controls
Wang J, et al. Hepatol Int^11^	Neutralizing antibodies at least 14 d after the second dose of inactivated whole-virion COVID-19 vaccines	388 and 165 patients with compensated and decompensated cirrhosis	Positive rates of neutralizing antibodies were 71.6% and 66.1% in compensated and decompensated cirrhosis groups
Iavarone M, et al. Dig Liver Dis^12^	Spike- and nucleocapsid-protein antibodies and Spike-specific T-cells responses at baseline, 21 d after the first and second doses and during follow-up	182 patients with cirrhosis (85% SARS-CoV-2-naïve) and 38 controls	After 2 doses of vaccine, anti-S titers were significantly lower in patients with cirrhosis *vs.* controls and in SARS-CoV-2-naïve *vs.* previously infected patients with cirrhosis . T-cell responses in patients with cirrhosis were similar to controls, although with different kinetics

Abbreviations: CLD, chronic liver disease; LT, liver transplant; mRNA, messenger RNA.

Moreover, there are few data regarding the risk and severity of SARS-CoV-2 breakthrough infection, following vaccination, in patients with CLD. Preliminary data suggest a reduced risk of breakthrough infection with vaccination in patients with cirrhosis.^13–15^ However, factors associated with the risk of breakthrough infection remain poorly defined in this patient group. These data are urgently needed to inform vaccination booster policy, particularly as many patients globally remain at risk of severe breakthrough infection due to vaccine hesitancy or lack of access to vaccination.

The pan-European COVID-19 vaccination and Biomarkers in cirrhosis And post-Liver Transplantation consortium was established through the coordinated efforts of the European Foundation for the Study of Chronic Liver Failure, the Foundation for Liver Research, the European Association for Study of the Liver, the European Liver and Intestine Transplant Association, and the European Liver Patients’ Association in December 2020, with the aim to determine the degree of protection provided by COVID-19 vaccination to liver disease patients.

The primary aim of the study was to determine if patients with CLD mount comparable humoral immune responses to healthy control participants following COVID-19 vaccination. Secondary aims included the determination of vaccine immunogenicity, neutralizing activity against VOCs, and breakthrough symptomatic COVID-19 in subgroups with cirrhosis, AILD, and post-LT.

## METHODS

### Study design and participants

**FIGURE 1:**
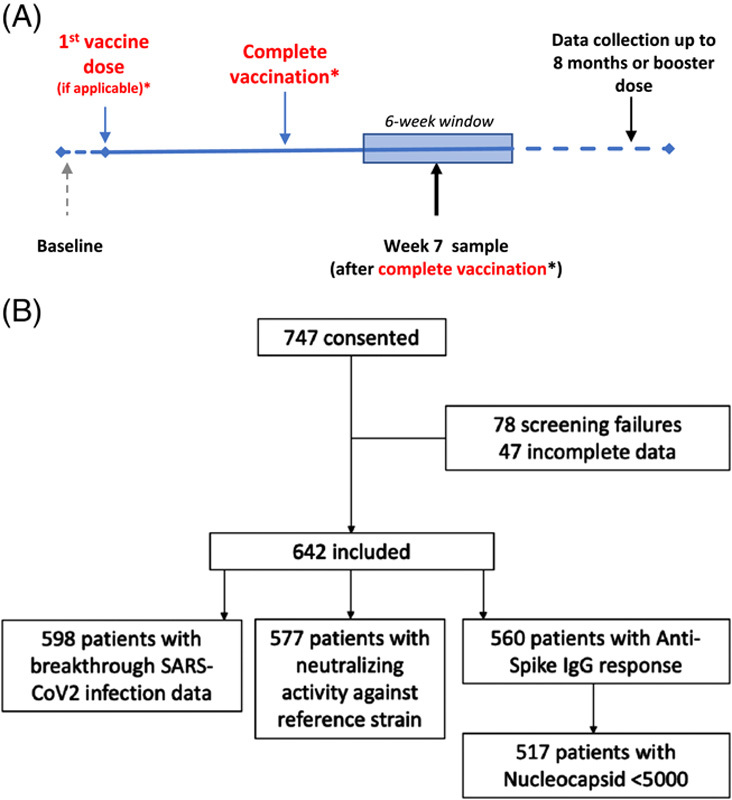
Study design and CONSORT diagram of recruitment and end points analyzed. COBALT is a prospective, multicenter, observational case-control study assessing immunogenicity of SARS-CoV-2 vaccine response in patients with cirrhosis, AILD, post-LT, and control. Participants were sampled within a 6-week window, between 4 and 10 weeks after the second SARS-CoV-2 vaccine dose (*or only vaccine dose if 1-dose regimen). Participants were followed up for breakthrough infection until booster vaccination or 8-month follow-up. Abbreviations: AILD, autoimmune liver disease; LT, liver transplant; COBALT, COVID-19 vaccination and Biomarkers in cirrhosis And post-Liver Transplantation.

COVID-19 vaccination and Biomarkers in cirrhosis And post-Liver Transplantation is a European, prospective, multicenter, observational, case-control study. The study design is outlined in Figure 1, and the protocol is available (Supplemental material, http://links.lww.com/HC9/A544). Recruitment for COVID-19 vaccination and Biomarkers in cirrhosis And post-Liver Transplantation took place in Italy, Spain, and the United Kingdom. Inclusion criteria were age 18 years or older, able to give written informed consent, diagnosis of cirrhosis (on imaging or liver biopsy), AILD (primary sclerosing cholangitis, primary biliary cholangitis, or autoimmune hepatitis) without cirrhosis, or post-LT (>6 mo) for cirrhosis, or control participant (absence of severe and uncontrolled cardiac, respiratory, liver, renal, or endocrine disease). Exclusion criteria were a history of COVID-19 (PCR-positive episode) or uncontrolled HIV infection. Patients were assessed for eligibility for the study, in the clinic or by telephone, at any point until 10 weeks after COVID-19 vaccination from May 8, 2021 to September 10, 2021.

### Data collection and biological sampling

The following information was collected at the time of inclusion: demographic data (date of birth, sex, race, and ethnicity); medical history [smoking status, drinking habits, date of onset/diagnosis and etiology of liver disease, history of decompensated cirrhosis (if applicable: jaundice, ascites, HE, peripheral edema, and bacterial infection), and hepatocellular cancer (if applicable)]; comorbidities (diabetes, hypertension, chronic lung disease, cardiovascular disease and clinically significant abnormalities observed in laboratory tests, or physical examination, as judged by the principal investigator), medications, and vaccine regimen received by participants [mRNA vaccines (2 doses of BNT162b2 Pfizer-BioNTech or mRNA-1273 Moderna), adenoviral vaccines (1 dose of Ad26.COV2.S Janssen/Johnson & Johnson or 2 doses of AZD1222 Oxford-AstraZeneca), or heterologous combinations]. Data were entered electronically into a predesigned electronic Case Report Form, maintained by the European Foundation for the Study of Chronic Liver Failure Data Management Center.

All participants underwent blood sampling at 7 ± 3 weeks following the second vaccine dose (or initial vaccine dose for 1-dose regimens) for laboratory analyses (outlined in more detail below). Immunological assays were conducted at the Roger Williams Institute of Hepatology, UK. Hematology (full blood count and coagulation) and biochemistry (liver and renal function) profiles were processed at the local center, and the results were clinician-reported.

### Participant follow-up

Participants were followed up after the second vaccination dose for 8 months or until the third vaccine dose (booster), whichever was sooner. Participants were contacted by telephone and asked about episodes of breakthrough SARS-CoV-2 infection (PCR or antigen test confirmed), specifically, date of infection, requirement for hospitalization/organ support, and outcome.

### Laboratory methods

#### Anti-Spike/RBD IgG and IgM immunoassays

Serum concentrations of IgG directed against SARS-CoV-2 Spike, SARS-CoV-2 S1 receptor binding domain (RBD), SARS-CoV-2 Nucleocapsid, and Spike from the following 6 human CoVs (SARS-CoV-1, Middle East Respiratory Syndrome-CoV, HCoV-229E, HCoV-HKU1, HCoV-NL63, and HCoV-OC43) were measured using an electrochemoluminescent immunoassay from meso scale discovery (MSD, V-PLEX COVID-19 CoV Panel 3 Kit, K15399U, Meso Scale Diagnostic, Maryland, USA). All serum samples were diluted 1:5000 before quantification, according to the manufacturer’s instructions, and assays were performed without modification. All data were normalized by log10 transformation before analysis. To prevent batch effects, all the samples were run using kits belonging to the same lots, to avoid lot-to-lot variability, and samples from the different groups were allocated so that each plate contained representation from all the groups.

To avoid bias due to the inclusion of subjects likely to have had asymptomatic COVID-19 before recruitment, participants with serum concentrations of anti-SARS-CoV-2 Nucleocapsid IgG antibodies > = 5000 U were excluded from subsequent analyses. This cutoff was suggested by the kit’s manufacturer as the optimal discriminant anti-N IgG threshold by the area under the receiver operating characteristic (AUROC) analysis, comparing pre-COVID negative serum samples to PCR-confirmed COVID-positive serum samples within 7–14 days of infection.

#### Neutralization assays (ACE2 binding and live virus)

The surrogate neutralization capacity of anti-SARS-CoV-2 Spike and anti-SARS-CoV-2 S1 RBD antibodies was measured using a pseudoviral neutralization assay based on competitive inhibition of angiotensin-converting enzyme-2 (ACE2) binding to viral strain-specific test Ags. The assay was obtained from meso scale discovery and specifically targeted SARS-CoV-2 Spike and RBD from the viral vaccine reference strain but also Spike from the Alpha, Beta, Gamma, Delta, and Omicron BA.1 viral variants as specified by the manufacturer (MSD V-PLEX SARS-CoV-2 Panel 24 Kit, K15578U). The samples were diluted 1:12.5 before quantification, and the assay was performed according to the manufacturer’s instructions without any modifications. The resulting percentage data were normalized by Logit transformation prior to analysis.

Neutralization assays against live SARS-CoV-2 wild-type virus were also performed using the microneutralization assay described by Algaissi and Hashem.^16^ The virus used for this assay was the clinical isolate SARS-CoV-2/human/NLD/Leiden-0008/2020 (GenBank accession number: MT705206.1). Neutralization titer was calculated by dividing the number of positive wells with complete inhibition of the virus-induced cytopathogenic effect, by the number of replicates, and adding 2.5 to stabilize the calculated ratio. The neutralizing antibody titer was defined as the log2 reciprocal of this value. All neutralization titers above 5 were considered positive.

#### Measurement of cytokines by multiplex analysis

A multiplex panel of 29 cytokines and chemokines was measured by Luminex following the manufacturer’s instructions (R&D-Systems/Bio-Techne, Abingdon-Oxford, UK), using a MAGPix instrument with xPonent v4.2 software (LuminexCorp, ’s-Hertogenbosch, the Netherlands). This multiplex panel included the following analytes: a proliferation-inducing ligand/TNF ligand superfamily member 13, B-cell activating factor (BAFF)/TNF ligand superfamily member 13B, chemokine ligand 2/monocyte chemoattractant protein 1, chemokine ligand 3/macrophage inflammatory protein 1α, chemokine ligand 4/macrophage inflammatory protein 1β, cluster of differentiation 40 ligand/TNF ligand superfamily member 5, C-X-C motif chemokine ligand 10/interferon gamma-induced protein 10, interferon gamma, IL-1α, IL-1β, IL-1ra, IL-2, IL-4, IL-5, IL-6, IL-8/CXCL8, IL-10, IL-12p70, IL-13, IL-15, IL-17A, IL-18, IL-23, IL-27, IL-28A, IL-28B, IL-33, lymphotoxin-α/TNFβ, and TNFα. All cytokines were quantified with 7-point standard curves built using a 5-parameter logistic regression model. Values that were assessed as “below the lower limit of quantitation” were not excluded for analysis but were, instead, assigned a standard half-minimum value as an arbitrarily lower threshold of detection.

Cytokines with low detection rate (>=25% undetectable values) and proportionally comparable distributions of detectable and undetectable samples across the 4 study groups (by chi-square analysis) were excluded from further analyses. Cytokines with low detection rates that displayed a significantly skewed distribution of detectable and undetectable samples across study groups were, however, maintained in all the models, as this group-specific bias was considered to be meaningful from a biological perspective.

### Statistical analyses

All data analyses were prespecified in the Statistical Analysis Plan, available in the Supplemental data, http://links.lww.com/HC9/A544.

#### Descriptive analyses and humoral responses to COVID-19 vaccination

Discrete variables were reported as counts (%); continuous variables normally distributed as mean (SD) and nonnormally distributed were summarized by median [25th percentile–75th percentile (p25–p75)]. In univariable statistical comparisons, associations between categorical variables were tested using Pearson chi-square test or log-linear models depending on data complexity. Concentrations of anti-Spike/RBD IgM, IgG, and IgG/IgM ratios at week 7 ± 3 following COVID-19 vaccination were normalized by log10 transformation. Percentages of viral strain-specific ACE2 binding neutralization were normalized by Logit transformation. The student *t*-test or general linear models (ANOVA or ANCOVA adjusted by age, sex, and time of sample collection after the second vaccine dose followed by Bonferroni-corrected pairwise comparisons between covariate-adjusted estimated group means) were used to evaluate group comparisons between cirrhosis, AILD, post-LT or control groups, and between all subgroups at week 7 ± 3 following COVID-19 vaccination. The Wilcoxon-Mann-Whitney or the Kruskal-Wallis tests were used for univariable comparisons when continuous variables were not normally distributed. Spearman’s rank correlations were used to evaluate the relationship between continuous variables and the degree of response determined by antibody levels. Linear relationships were assessed by Pearson’s product-moment correlation.

#### Factors associated with COVID-19 vaccine response

To study independent associations of antibody response in each independent group of study, univariable general linear models were performed, including demographic, clinical, drug-related, and biochemical data. Only covariates showing clinical and statistical significance in univariable models or participating as a confounding factor for the variable of interest were included in the final stepwise multivariable models.

#### Correlates of risk for breakthrough SARS-CoV-2 infection

To identify risk factors for breakthrough SARS-CoV-2 infection in each study group, the Cox proportional hazard model was used. Independent covariates were included in the models when showing statistical significance or confounding. proportional hazard assumptions were explored by testing zph (correlation between the Schoenfeld residuals and survival time), and proportional hazard assumptions were met for all variables included in the models.

#### Software and data quality assurance

Statistical analyses were carried out using SAS v 9.4, R v 4.1.0, SPSS v26/27, and SIMCA v15/v17, depending on package availability and functionality, with the cutoff for statistical significance set at 0.05.

#### Research reproducibility approach

All analyses were reproducibly performed and were hosted in the European Foundation for the Study of Chronic Liver Failure repository, which is publicly available on demand.

### Ethics

The study was conducted in accordance with the recommendations for physicians involved in research on human participants adopted by the 18th World Medical Assembly, Helsinki 1964, as revised and recognized by governing laws and EU Directives. The study was approved by ethical review boards at all study sites. Each participant’s consent to participate in the study was obtained after a full explanation was given. The right of the participant to refuse to participate in the study without giving reasons was respected. Details of ethical approvals are provided in the Supplemental materials, http://links.lww.com/HC9/A544.

## RESULTS

### Participants

A total of 767 participants consented from 25 centers in Italy, Spain, and the United Kingdom; 78 subjects were screening failures due to inclusion outside of the recruitment window, and 47 were excluded due to incomplete data, leaving 642 participants for analysis: 325 patients with cirrhosis (94% mRNA vaccine and 6% viral vaccine), 120 AILD (autoimmune hepatitis 66, primary biliary cholangitis 17, autoimmune hepatitis/primary biliary cholangitis overlap 15, primary sclerosing cholangitis 4, autoimmune hepatitis/primary sclerosing cholangitis overlap 1, and other 17; 77% mRNA vaccine and 23% viral vaccine), 146 post-LT (96% mRNA vaccine, 3% viral vaccine, and 1% heterologous combination), and 51 control (72% mRNA vaccine, 24% viral vaccine, and 4% heterologous combination). Of the AILD patients, 64 (53.3%) were treated with ursodeoxycholic acid. The CONSORT diagram of recruitment and end points analyzed is displayed in Figure 1. Typically, informed consent and biological sampling occurred on the same day. Participant characteristics are presented in Table 2. Several variables were significantly different between groups, consistent with the underlying disease state. Of note, age was significantly different between groups of study, with control participants younger than the disease groups. Alcohol and tobacco consumption was lower in the control group than in cirrhosis and LT groups. In terms of comorbidities, the prevalence of arterial hypertension, coronary artery disease, chronic kidney disease, and diabetes mellitus was greater in cirrhosis and LT groups than in control; other chronic systemic diseases were more prevalent in AILD patients than in other groups.

**TABLE 2:** Baseline characteristics of study population

Parameter	Cirrhosis (n = 325)	Autoimmune liver disease (n = 120)	Liver transplant (n = 146)	Healthy individuals (n = 51)	*p*
Sex, Male (n, %)	223/325 (69)	28/120 (23)	105/146 (72)	22/51 (43)	**<0.0001**
Age (y; median, p25–p75)	61 (56, 69)	59 (48, 65)	62 (55, 70)	52 (39, 65)	**<0.0001**
Sampling time postvaccination (d; median, p25–p75)	56 (47, 63)	55 (47, 61)	53 (48, 65)	53 (40, 62)	**0.3040**
Race (n, %)	—	—	—	—	1
White	317/320 (99)	119/120 (99)	144/145 (99)	49/49 (100)	—
Black or Afro-American	3/320 (1)	1/120 (1)	1/145 (1)	0/49 (0)	—
Ethnicity (n, %)	—	—	—	—	**0.0301**
North European	36/324 (11)	13/120 (11)	6/146 (4)	2/51 (4)	—
Mediterranean	272/324 (85)	98/120 (82)	135/146 (93)	46/51 (90)	—
Latin American	7/324 (2)	7/120 (6)	1/146 (1)	0/51 (0)	—
Other	7/324 (2)	1/120 (1)	4/146 (3)	3/51 (6)	—
Alcohol consumption (n, %)	—	—	—	—	**<0.0001**
No	125/293 (43)	111/119 (93)	101/146 (69)	46/49 (94)	—
Former drinker	126/293 (43)	2/119 (2)	39/146 (27)	1/49 (2)	—
Current drinker	42/293 (14)	6/119 (5)	6/146 (4)	2/49 (4)	—
Tobacco consumption (n, %)	—	—	—	—	**0.0317**
No	162/293 (55)	82/119 (69)	83/146 (57)	33/49 (67)	—
Former smoker	63/293 (22)	21/119 (18)	42/146 (29)	7/49 (14)	—
Current smoker	66/293 (23)	16/119 (13)	21/146 (14)	9/49 (18)	—
Etiology (n, %)					
Alcohol	170/325 (52)	N/A	N/A	N/A	—
HBV or HCV	128/325 (39)	—	—	—	—
NAFLD/NASH	65/325 (20)	—	—	—	—
Autoimmune^a^	40/325 (4)	—	—	—	—
Others	17/325 (5)	—	—	—	—
Years since diagnosis (n, %)		—	—	—	**<0.0001**
<1	32/270 (12)	6/74 (8)	0/126 (0)	NA	—
1–5	77/270 (29)	22/74 (30)	21/126 (17)	—	—
>5	161/270 (60)	46/74 (62)	105/126 (83)	—	—
History of acute decompensation (n,%)	150/270 (56)	0/87 (0)	61/117 (52)	NA	**<0.0001**
HCC at time of sampling	51/272 (19)	—	—	—	**<0.0001**
Within Milan criteria	31/51 (61)	0/113 (0)	NA	NA	—
Without Milan criteria	20/51 (39)	—	—	—	—
HCC on explant	—	—	39/139 (28)	—	—
Within Milan criteria	NA	NA	35/39 (90)	NA	—
Without Milan criteria	—	—	4/39 (10)	—	—
MELDNa score week 7 (median, p25–p75)	(311) 11 (8, 16)	NA	NA	NA	—
MELDNa ≥ 14 (n, %)	102/311 (33)	—	—	—	—
Child-Turcotte-Pugh class week 7 (n, %)					
A	210/325 (65)	NA	NA	NA	—
B	97/325 (30)	—	—	—	—
C	18/325 (6)	—	—	—	—
Comorbidities (n, %)					
COPD	21/294 (7)	2/119 (2)	7/146 (5)	1/49 (2)	0.1004
Heart failure	8/294 (3)	0/119 (0)	1/146 (1)	0/49 (0)	0.1699
Arterial hypertension	117/294 (40)	24/119 (20)	64/146 (44)	10/49 (20)	**<0.0001**
Coronary artery disease	25/294 (9)	3/119 (3)	6/146 (4)	0/49 (0)	**0.0166**
Chronic kidney disease	14/294 (5)	2/119 (2)	13/146 (9)	1/49 (2)	**0.0467**
Cerebrovascular disease	17/294 (6)	1/119 (1)	6/146 (4)	1/49 (2)	0.1061
Diabetes mellitus	74/294 (25)	3/119 (3)	44/146 (30)	3/49 (6)	**<0.0001**
Psychiatric disorders	19/294 (7)	4/119 (3)	4/146 (3)	1/49 (2)	0.2778
Other chronic systemic disease	75/294 (26)	48/119 (40)	36/146 (25)	13/49 (27)	**0.0172**
Laboratory (median, p25–p75; mean, SD)					
Albumin (g/dL)	3.9 (3.4, 4.3)	4.3 (4.1, 4.5)	4.20 (4, 4.5)	4.21 (4.2, 4.45)	**<0.0001**
AST (U/L)	36 (25, 56)	27 (22, 35)	23 (18, 30)	20 (17, 29)	**<0.0001**
ALT (U/L)	26 (18, 39)	25 (18, 36)	21 (15, 34)	18 (12, 40)	**0.0201**
Alkaline phosphatase (U/L)	112 (84, 154)	89 (66, 124)	91.5 (64, 124)	86 (64, 111)	**<0.0001**
GGT (U/L)	58 (29, 118)	28 (15, 60)	30 (17, 76)	21 (13, 28)	**<0.0001**
Total Bilirubin (mg/dL)	1.1 (0.7, 1.9)	0.6 (0.5, 0.8)	0.66 (0.5, 1)	0.52 (0.3, 0.62)	**<0.0001**
Creatinine (mg/dL)	0.8 (0.7, 1)	0.8 (0.6, 0.9)	1.08 (0.9, 1.3)	0.82 (0.71, 0.9)	**<0.0001**
Sodium (mEq/L)	139 (136, 140)	141 (139, 142)	141 (139, 142)	139 (139, 141)	**<0.0001**
Total cholesterol (mg/dL)	151 (130, 185)	196 (171, 226)	169 (145, 201)	200 (175, 231)	**<0.0001**
HDL-Cholesterol (mg/dL)	49 +/−21	61 +/− 22	43.1 +/− 15.78	54.3 +/− 9.56	**<0.0001**
LDL-Cholesterol (mg/dL)	90 +/− 55	113 +/− 32	369.4 +/− 1847.37	130.2 +/− 26.93	0.3795
Triglycerides (mg/dL)	85 (65, 104)	90 (71, 126)	103 (76, 163)	81.5 (65, 112)	**0.0002**
C-reactive protein (mg/L)	5.7 (1.6, 13)	2 (1–3)	2.4 (1–7)	2.38 (1.43, 40.5)	**0.0285**
Hemoglobin (g/dL)	12 +/− 3.1	13.6 +/− 2.7	13.7 +/− 2.25	13.2 +/− 2.79	**<0.0001**
Leukocyte (× 10^9cells/L)	5.03 (3.7, 6.8)	6.01 (4.98, 7.13)	5.68 (4.4, 7)	6.49 (5.96, 8.12)	**<0.0001**
Lymphocytes (× 10^9cells/L)	1.18 (0.8, 1.9)	1.81 (1.4, 2.3)	1.52 (1.04, 2)	2.03 (1.63, 2.54)	**<0.0001**
Monocytes (× 10^9cells/L)	0.48 (0.4, 0.7)	0.5 (0.38, 0.63)	0.46 (0.38, 0.61)	0.60 (0.60, 0.68)	0.062
Neutrophils (× 10^9cells/L)	3.03 (2, 4.1)	3.2 (2.7, 4.1)	3.16 (2.41, 4.34)	3.53 (3.01, 4.1)	0.0865
Neutrophil/Lymphocyte ratio (%)	2.95 (1.9, 12.3)	2.0 (1.3, 2.8)	2.3 (1.7, 3.2)	2.1 (1.6, 4.7)	**0.0009**
Platelet (× 10^3cells/uL)	10426 +/− 38616	4501 +/− 31377	4425 +/− 26288	262.4 +/− 95.83	0.3328
INR	1.2 (1.05, 1.3)	0.97 (0.9, 1.03)	1 (1, 1.08)	1.05 (0.91, 1.11)	**<0.0001**
Prothrombin time (sec)	14 (12, 16)	11.5 (11, 12.3)	12.5 (12.5, 13.7)	11.4 (11.3, 14)	**<0.0001**
Immunosuppressive drugs (n, %)					
Steroids	14/325 (4)	31/120 (26)	19/146 (13)	0/51 (0)	**<0.0001**
Calcineurin antagonist	4/325 (1)	8/120 (7)	128/146 (88)	0/51 (0)	**<0.0001**
Mycophenolate	4/325 (1)	9/120 (8)	73/146 (50)	0/51 (0)	**<0.0001**
Azathioprine or 6-MP	4/325 (1)	35/120 (29)	3/146 (2)	0/51 (0)	**<0.0001**
Other	0/325 (0)	1/120 (1)	17/146 (12)	0/51 (0)	**<0.0001**
Vaccine brand, n (%)	—	—	—	—	**<0.0001**
mRNA	292/312 (94)	90/117 (77)	134/139 (96)	33/46 (72)	—
Viral	20/312 (6)	27/117 (23)	4/139 (3)	11/46 (24)	—
Heterologous combination	0/312 (0)	0/117 (0)	1/139 (1)	2/46 (4)	—
Cytokines					
IL-6	−0.31 (−2.41, 0.21)	−2.41 (−2.41, −1.28)	−2.23 (−2.41, −0.20)	−2.41 (−2.41, −2.41)	**<0.0001**
BAFF/BLyS/TNFSF13B	2.54 (2.42, 2.67)	2.39 (2.30, 2.45)	2.43 (2.35, 2.52)	2.42 (2.29, 2.49)	**<0.0001**
CXCL10/IP-10/CRG-2	1.01 (0.79, 1.20)	0.74 (0.60, 0.92)	0.83 (0.67, 1.08)	0.64 (0.54, 0.77)	**<0.0001**
TNF-alpha	0.20 (−0.08, 0.40)	−0.07 (−0.38, 0.20)	0.28 (0.06, 0.43)	−0.63 (−2.05, −0.04)	**<0.0001**
APRIL/TNFSF13	2.55 (2.39, 2.74)	2.76 (2.65, 2.83)	2.59 (2.45, 2.71)	2.75 (2.62, 2.84)	**<0.0001**
CD40Ligand/TNFSF5	3.09 (2.82, 3.31)	3.35 (3.17, 3.47)	2.98 (2.59, 3.26)	3.33 (3.18, 3.46)	**<0.0001**
IL-18/IL-1F4	1.9 +/− 0.25	1.7 +/− 0.24	1.9 +/− 0.23	1.7 +/− 0.19	**<0.0001**
CCL2/JE/MCP-1	2.02 (1.87, 2.17)	2.16 (2.01, 2.30)	2.11 (1.99, 2.24)	2.16 (2.00, 2.28)	**<0.0001**
IL-27	1.04 (−0.25, 1.95)	−0.25 (−0.25, 0.27)	−0.25 (−0.25, 1.71)	−0.25 (−0.25, 0.79)	**<0.0001**
IL-8/CXCL8	0.87 (0.57, 1.18)	0.75 (0.56, 1.03)	0.72 (0.47, 0.95)	0.62 (0.40, 0.81)	**<0.0001**
CCL4/MIP-1 beta	1.52 (1.02, 1.69)	1.63 (1.37, 1.77)	1.51 (0.72, 1.71)	1.50 (0.72, 1.71)	**0.0041**
IL-28B/IFN-lambda3	0.41 (0.41, 0.41)	0.41 (0.41, 0.41)	0.41 (0.41, 0.41)	0.41 (0.41, 0.41)	**0.001**
IL-15	−2.87 (−2.87, −0.85)	−2.87 (−2.87, −0.41)	−2.87 (−2.87, −0.37)	−2.87 (−2.87, −2.87)	**0.0034**
IL-23	−0.40 (−0.40, 0.98)	−0.40 (−0.40, −0.40)	−0.40 (−0.40, 0.71)	−0.40 (−0.40, 0.11)	**0.0439**
IL-2	−1.80 (−1.80, 0.05)	−1.80 (−1.80, −0.26)	−1.80 (−1.80, 0.22)	−1.80 (−1.80, −1.80)	**0.0176**
Lymphotoxin-alpha/TNF-beta	−2.05 (−2.05, −2.05)	−2.05 (−2.05-−2.05)	−2.05 (−2.05, −2.05)	−2.05 (−2.05, −2.05)	**0.0031**
IL-17/IL-17A	−2.24 (−2.24, −2.24)	−2.24 (−2.24, −2.24)	−2.24 (−2.24, −2.24)	−2.24 (−2.24, −2.24)	**0.0206**
IL-12p70	−1.65 (−1.65, −0.09)	−1.65 (−1.65, −0.16)	−1.65 (−1.65, 0.13)	−1.65 (−1.65, −1.65)	**0.0098**
IL-1ra/IL-1F3	2.31 (2.17, 2.49)	2.35 (2.25, 2.49)	2.33 (2.18, 2.56)	2.44 (2.27, 2.54)	**0.0165**
IL-5	−1.18 (−1.18,	−1.18 (−1.18, −1.18)	−1.18 (−1.18, −1.18)	−1.18 (−1.18, −1.18)	0.1217
IL-13	0.49 (0.49, 0.49)	0.49 (0.49, 1.48)	0.49 (0.49, 1.48)	0.49 (0.49, 0.49)	0.1045
IL-33	−2.66 (−2.66, −2.66)	−2.66 (−2.66, −2.66)	−2.66 (−2.66, −2.66)	−2.66 (−2.66, −2.66)	0.3376
IL-4	−0.82 (−0.82, −0.82)	−0.82 (−0.82, −0.82)	−0.82 (−0.82, −0.82)	−0.82 (−0.82, −0.82)	0.2805
IFN-gamma	−3.12 (−3.12, −1.62)	−3.12 (−3.12, −1.45)	−3.12 (−3.12, −1.62)	−3.12 (−3.12, −3.12)	0.1573
IL-10	−2.16 (−2.16, −0.64)	−2.16 (−2.16, −0.67)	−2.16 (−2.16, −0.77)	−2.16 (−2.16, −1.03)	0.4703
CCL3/MIP-1 alpha	0.43 (0.43, 0.43)	0.43 (0.43, 0.43)	0.43 (0.43, 0.43)	0.43 (0.43, 0.43)	0.4774
IL-1alpha/IL-1F1	−1.51 (−1.51, −1.51)	−1.51 (−1.51, −1.51)	−1.51 (−1.51, −1.51)	−1.51 (−1.51, −1.51)	0.4409
IL-28A/IFN-lambda2	0.04 (0.04, 0.90)	0.04 (0.04, 0.53)	0.04 (0.04, 1.27)	0.04 (0.04, 0.53)	0.0038

Bold values indicate statistical significance.

*Notes:* ANOVA and Kruskal-Wallis test were used to compare continuous variables, while categorical variables were compared with the chi-square or Fisher exact test. *p*-value represents differences between all groups of the study.

a Includes autoimmune hepatitis, primary sclerosing cholangitis, and primary biliary cholangitis.

Abbreviations: ALT, alanine aminotransferase; AST, aspartate aminotranferase; 25th percentile–75th percentile (p25–p75), APRIL/TNFSF13, a proliferation-inducing ligand/TNF ligand superfamily member 13; BAFF/TNFSF13B, B-cell activating factor/TNF ligand superfamily member 13B; CCL2/MCP-1, chemokine ligand 2/monocyte chemoattractant protein 1; CCL3/MIP-1, chemokine ligand 3/macrophage inflammatory protein 1α; CCL4/MIP-1, chemokine ligand 4/macrophage inflammatory protein 1β; CD40L/TNFSF5, cluster of differentiation 40 ligand/TNF ligand superfamily member 5; COPD, Chronic obstructive pulmonary disease; CXCL10/IP-10, C-X-C motif chemokine ligand 10/interferon gamma-induced protein 10; GGT, gamma glutamyltransferase; IFN, IFNγ, interferon gamma; INR, international normalized ratio; MELDNa, Model for End Stage Liver Disease score-Na; mRNA, messenger RNA; NA, not applicable; 6-MP, 6-mercaptopurine; VOC, variant of concern.

### Humoral immune responses to COVID-19 vaccination in patients with cirrhosis and AILD, and post-LT

**FIGURE 2:**
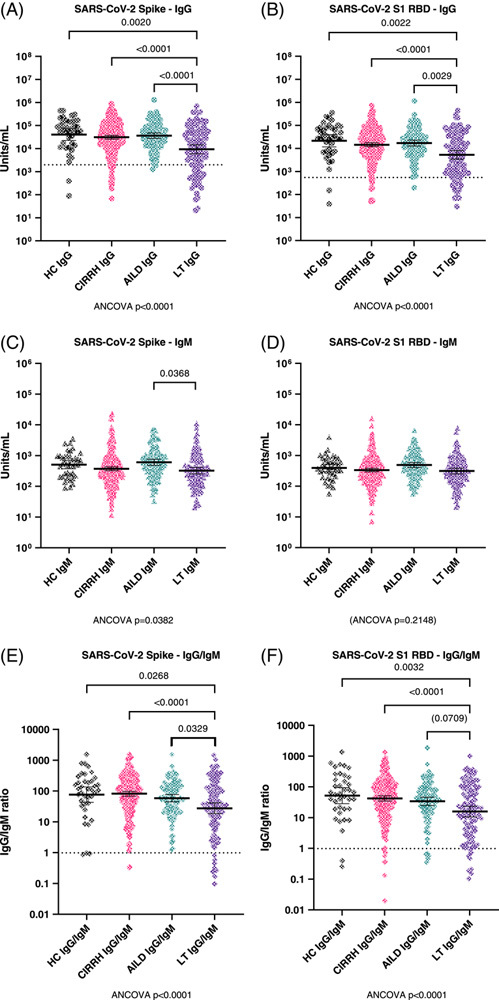
Serological measurements of anti-SARS-CoV-2 antibodies postvaccination. (A and B), Serum concentrations of anti-Spike and anti-RBD IgG antibodies (respectively) in healthy controls, patients with cirrhosis (CIRRH), autoimmune liver disease (AILD), and liver transplant (LT). All measurements were obtained using electrochemoluminescence-based meso scale discovery multiplex assays. Samples were diluted at 1:5,000 for quantification, as recommended by the manufacturer. All data are represented on log10 scale scatterplot graphs; lines and error bars represent geometric mean and 95% CI. All comparisons were assessed by ANCOVA, adjusted for age, sex, and time of sample collection postvaccination, followed by Bonferroni-corrected pairwise comparisons between covariate-adjusted estimated group means. Abbreviations: AILD, autoimmune liver disease; CIRRH, cirrhosis; LT, liver transplant; RBD, receptor binding domain.

A total of 43 patients presented anti-SARS-CoV-2 Nucleocapsid IgG antibodies ≥ 5000 units out of the 560 patients with anti-Spike IgG data, suggesting previous infection before vaccination. Therefore, the primary analysis of anti-Spike IgG response was conducted in 517 participants (Figure 2, panel A). Geometric mean anti-Spike IgG was significantly lower in post-LT patients compared to control [n = 106, 8609.0 (5482.86–13517.47) vs. n = 39, 47562.9 (28729.41–78742.63); ANCOVA after adjusting by age, sex, and time of sample collection *p* = 0.002], cirrhosis [n = 271, 30526.3 (25268.38–36878.30); *p* < 0.0001], and AILD [n = 101, 39444.0 (27911.67–55741.29); *p* < 0.0001] groups although no other between-group differences were noted. Similar findings were noted for anti-RBD IgG response (Figure 2, panel B). Data regarding humoral responses against SARS-CoV-2 nucleocapsid and other CoVs (including SARS-CoV-1 and Middle East Respiratory Syndrome ) are presented in Supplemental Figures 1–3, http://links.lww.com/HC9/A544.

### Factors associated with anti-Spike antibody levels in patients with cirrhosis and AILD, and post-LT

Multivariable modeling was conducted to identify variables associated with anti-Spike IgG response in each disease group (abbreviated data in Table 3, full data in Supplemental Table 1, http://links.lww.com/HC9/A544).

**TABLE 3:** Univariable and multivariable analyses of IgG Spike vaccine response

	Cirrhosis				Autoimmune liver disease^*^				Liver transplant			
	Univariable		Multivariable		Univariable		Multivariable		Univariable		Multivariable	
Parameter	E (95% CI)	*p*	E (95% CI)	*p*	E (95% CI)	*p*	E (95% CI)	*p*	E (95% CI)	*p*	E (95% CI)	*p*
Age	−0.02 (−0.03, -0.01)	**<0.0001**	−0.01 (−0.03, -0.00)	**0.0044**	−0.01 (−0.03, -0.00)	**0.0428**			−0.01 (−0.03, 0.01)	0.1828		
Race												
White	Ref.	0.1541	—	—	Ref.	**0.0462**	—	—	—	---	—	—
Black or Afro-American	0.57 (−0.21, 1.35)		—	—	−1.52 (−3.02, -0.03)	—	—	—	—	—	—	—
Alcohol consumption												
No	Ref.	—	—	—	Ref.	—	—	—	Ref.	—	—	—
Former drinker	−0.06 (−0.24, 0.13)	0.5566	—	—	−0.29 (−1.36, 0.79)	0.5940	—	—	−0.07 (−0.49, 0.35)	0.7529	—	—
Current drinker	−0.13 (−0.40, 0.14)	0.3505	—	—	0.50 (−0.13, 1.13)	0.1210	—	—	−0.94 (−1.79, −0.09)	**0.0301**	—	—
HCC	0.17 (−0.08, 0.41)	0.1769	—	—	---	---	—	—	−0.08 (−0.50, 0.34)	0.7117	—	—
Child-Turcotte-Pugh class												
A	0.22 (0.05, 0.39)	**0.0123**	0.23 (0.01, 0.45)	**0.0365**	NA	—	—	—	NA	—	—	—
B+C	Ref.	—	—	—	—	—	—	—	—	—	—	—
Comorbidities												
COPD	−0.27 (−0.61, 0.08)	0.1293	—	—	−0.50 (−1.58, 0.58)	0.3639	—	—	0.43 (−0.49, 1.36)	0.3532	—	—
Heart failure	−0.41 (−0.98, 0.16)	0.1543	—	—		—	—	—	—	—	—	—
Hypertension	−0.11 (−0.29, 0.07)	0.2365	—	—	−0.09 (−0.47, 0.29)	0.6358	—	—	−0.08 (−0.49, 0.32)	0.6823	—	—
Coronary disease	−0.05 (−0.35, 0.26)	0.7645	—	—	−0.39 (−1.27, 0.50)	0.3881	—	—	−1.04 (−2.05, −0.03)	**0.0441**	−1.08 (−1.96, −0.19)	**0.0177**
CRF	−0.32 (−0.71, 0.07)	0.1062	—	—	−0.02 (−1.11, 1.06)	0.9648	—	—	−0.04 (−0.74, 0.67)	0.9189	—	—
CVD	−0.18 (−0.55, 0.19)	0.3331	—	—	0.19 (−1.34, 1.71)	0.8097	—	—	−0.12 (−1.15, 0.91)	0.8196	—	—
DM	−0.21 (−0.41, −0.01)	**0.0417**	—	—	0.21 (−0.68, 1.10)	0.6359	—	—	−0.32 (−0.78, 0.14)	0.1705	—	—
Psychiatric	−0.15 (−0.52, 0.23)	0.4497	—	—	0.45 (−0.44, 1.33)	0.3208	—	—	−0.54 (−1.56, 0.49)	0.3030	—	—
Other disease	−0.01 (−0.20, 0.19)	0.9578	—	—	0.12 (−0.19, 0.43)	0.4408	—	—	−0.24 (−0.69, 0.21)	0.2961	—	—
Laboratory												
Albumin	−0.01 (−0.12, 0.11)	0.9128	—	—	0.40 (−0.06, 0.87)	0.0897	—	—	0.21 (−0.34, 0.76)	0.4489	—	—
AST	−0.00 (−0.00, 0.00)	0.1771	—	—	−0.00 (−0.01, 0.01)	0.9962	—	—	0.00 (−0.00, 0.01)	0.5602	—	—
ALT	−0.00 (−0.00, 0.00)	0.2120	—	—	0.00 (−0.00, 0.00)	0.3279	—	—	0.00 (−0.00, 0.01)	0.3037	—	—
ALP	−0.00 (−0.00, 0.00)	0.7740	—	—	−0.00 (−0.00, 0.00)	0.9062	—	—	−0.00 (−0.01, −0.00)	**0.0222**	—	—
GGT	0.00 (−0.00, 0.00)	0.5749	—	—	0.00 (−0.00, 0.00)	0.1077	—	—	−0.00 (−0.00, −0.00)	**0.0140**	—	—
Total Bilirubin	0.02 (−0.02, 0.05)	0.3426	—	—	−0.12 (−0.39, 0.16)	0.3985	—	—	0.05 (−0.25, 0.36)	0.5938	—	—
Creatinine	−0.20 (−0.42, 0.02)	0.0753	—	—	−0.01 (−0.77, 0.75)	0.9829	—	—	−0.13 (−0.68, 0.42)	0.1582	—	—
Sodium	0.02 (−0.01, 0.04)	0.1575	—	—	−0.03 (−0.10, 0.04)	0.4245	—	—	−0.01 (−0.03, 0.00)	0.1182	—	—
Total cholesterol	−0.00 (−0.00, 0.00)	0.5762	—	—	−0.00 (−0.01, 0.00)	0.4716	—	—	0.00 (−0.01, 0.01)	0.7344	—	—
HDL-Cholesterol	0.00 (−0.01, 0.01)	0.5118	—	—	−0.00 (−0.01, 0.01)	0.4349	—	—	0.01 (−0.01, 0.03)	0.4339	—	—
LDL-Cholesterol	−0.00 (−0.00, 0.00)	0.7743	—	—	−0.00 (−0.01, 0.01)	0.8960	—	—	−0.00 (−0.01, 0.01)	0.3996	—	—
Triglycerides	−0.00 (−0.00, 0.00)	0.5587	—	—	0.00 (−0.00, 0.01)	0.1612	—	—	−0.00 (−0.00, 0.00)	0.9674	—	—
C-reactive protein	−0.00 (−0.00, 0.00)	0.3622	—	—	−0.06 (−0.40, 0.29)	0.6733	—	—	−0.01 (−0.03, −0.00)	**0.0438**	—	—
Hemoglobin	0.03 (−0.00, 0.05)	0.0633	—	—	−0.01 (−0.07, 0.05)	0.7048	—	—	0.07 (−0.01, 0.16)	0.1036	—	—
Leukocyte	0.00 (−0.00, 0.00)	0.8283	—	—	0.00 (−0.00, 0.00)	0.8272	—	—	0.06 (−0.01, 0.12)	0.0816	—	—
Lymphocytes	0.10 (0.00, 0.20)	**0.0400**	—	—	0.00 (−0.20, 0.21)	0.9649	—	—	0.15 (−0.03, 0.33)	0.0983	—	—
Monocytes	0.15 (−0.11, 0.42)	0.2566	—	—	−0.49 (−1.42, 0.44)	0.3010	—	—	0.73 (−0.57, 2.03)	0.2596	—	—
Neutrophils	−0.05 (−0.10, 0.00)	0.0597	—	—	−0.02 (−0.14, 0.11)	0.8043	—	—	0.09 (−0.01, 0.20)	0.0853	—	—
Neutrophil/Lymphocyte	0.01 (0.00, 0.01)	**0.0128**	—	—	0.00 (−0.01, 0.02)	0.7056	—	—	0.00 (−0.00, 0.01)	0.4235	—	—
Platelet	−0.00 (−0.00, 0.00)	0.4440	—	—	−0.00 (−0.00, 0.00)	0.8196	—	—	−0.00 (−0.00, 0.00)	0.9572	—	—
INR	−0.04 (−0.28, 0.20)	0.7705	—	—	−0.45 (−1.47, 0.56)	0.3777	—	—	0.08 (−1.02, 1.18)	0.8885	—	—
Prothrombin time	0.01 (−0.14, 0.16)	0.9030	—	—	−0.25 (−0.81, 0.32)	0.3749	—	—	0.77 (−0.25, 1.80)	0.1350	—	—
Immunosuppressive drugs	−0.22 (−0.70, 0.27)	0.3812	—	—	−0.09 (−0.40, 0.21)	0.5454	—	—	0.71 (−0.21, 1.63)	0.1272	—	—
Steroids	−0.06 (−0.48, 0.36)	0.7816	—	—	−0.12 (−0.45, 0.21)	0.4694	—	—	−0.56 (−1.17, 0.05)	0.0714	—	—
Calcineurin antagonist	−0.68 (−1.64, 0.27)	0.1616	—	—	0.23 (−0.46, 0.93)	0.5041	—	—	0.45 (−0.12, 1.03)	0.1205	—	—
Mycophenolate	−0.28 (−1.24, 0.68)	0.5619	—	—	−0.15 (−0.68, 0.38)	0.5777	—	—	−0.72 (−1.09, −0.35)	**0.0002**	−0.78 (−1.13, −0.43)	**<0.0001**
Azathioprine or 6-MP	0.06 (−0.62, 0.74)	0.8613	—	—	−0.12 (−0.44, 0.21)	0.4814	—	—	1.26 (−0.77, 3.28)	0.2211	—	—
Other	---	—	—	—	0.29 (−1.23, 1.81)	0.7071	—	—	−0.19 (−0.79, 0.41)	0.5306	—	—
Vaccine brand												
mRNA	Ref.	—	—	—	Ref.	—	Ref.	—	Ref.	—	—	—
Viral	−0.52 (−0.92, −0.12)	**0.0107**	—	—	−0.47 (−0.84, −0.10)	**0.0128**	−0.47 (−0.84, 0.10)	0.0128	0.10 (−1.35, 1.55)	0.8913	—	—
Heterologous	—	—	—	—	—	—	—	—	0.35 (−1.69, 2.39)	0.7325	—	—
Cytokines												
IL-6	−0.11 (−0.18, −0.04)	0.0013	−0.10 (−0.18, −0.01)	**0.0355**	−0.15 (−0.33, 0.03)	0.0921	—	—	−0.15 (−0.33, 0.02)	0.0899	—	—
IL-33^a^	0.01 (−0.06, 0.08)	0.7609	—	—	−0.03 (−0.18, 0.11)	0.6295	—	—	−0.00 (−0.24, 0.24)	0.9840	—	—
IL-8/CXCL8	−0.02 (−0.17, 0.14)	0.8430	—	—	0.02 (−0.26, 0.30)	0.9029	—	—	−0.05 (−0.42, 0.31)	0.7716	—	—
CXCL10/IP-10/CRG-2	−0.36 (−0.62, −0.10)	**0.0076**	—	—	−0.36 (−0.96, 0.23)	0.2297	—	—	−0.56 (−1.33, 0.20)	0.1465	—	—
IL-10^a^	−0.08 (−0.17, 0.01)	0.0755	—	—	−0.06 (−0.23, 0.12)	0.5012	—	—	−0.10 (−0.34, 0.14)	0.4201	—	—
IL-27	−0.00 (−0.07, 0.07)	0.9670	—	—	−0.17 (−0.35, 0.01)	0.0685	—	—	−0.05 (−0.24, 0.15)	0.6432	—	—
IL-2^a^	0.02 (−0.06, 0.11)	0.5724	—	—	0.03 (−0.14, 0.20)	0.7220	—	—	0.08 (−0.11, 0.27)	0.4153	—	—
IFN-gamma	0.02 (−0.03, 0.07)	0.3772	—	—	0.01 (−0.08, 0.10)	0.8025	—	—	0.12 (−0.04, 0.28)	0.1312	—	—
IL-1ra/IL-1F3	0.13 (−0.19, 0.45)	0.4194	—	—	−0.13 (−0.86, 0.59)	0.7158	—	—	−0.02 (−0.80, 0.76)	0.9590	—	—
CCL3/MIP-1 alpha^a^	0.19 (−0.32, 0.70)	0.4732	—	—	0.23 (−0.87, 1.33)	0.6810	—	—	−0.24 (−1.16, 0.69)	0.6149	—	—
CCL4/MIP-1 beta	−0.05 (−0.24, 0.15)	0.6390	—	—	0.08 (−0.29, 0.44)	0.6833	—	—	−0.10 (−0.50, 0.30)	0.6315	—	—
IL-1 alpha/IL-1F1^a^	−0.09 (−0.41, 0.22)	0.5643	—	—	—	—	—	—	−0.16 (−1.30, 0.98)	0.7837	—	—
IL-4^a^	−0.06 (−0.20, 0.07)	0.3694	—	—	0.00 (−0.28, 0.29)	0.9780	—	—	−0.07 (−0.47, 0.34)	0.7412	—	—
IL-17/IL-17A	0.01 (−0.08, 0.11)	0.7599	—	—	−0.04 (−0.33, 0.24)	0.7560	—	—	0.24 (0.02, 0.46)	**0.0308**	—	—
APRIL/TNFSF13	0.02 (−0.32, 0.37)	0.8880	—	—	−0.10 (−1.18, 0.97)	0.8530	—	—	0.48 (−0.43, 1.38)	0.2996	—	—
BAFF/BLyS/TNFSF13B	−0.23 (−0.63, 0.17)	0.2676	—	—	−0.12 (−1.28, 1.04)	0.8381			−2.58 (−3.83, −1.33)	**<0.0001**	−2.34 (−3.51, −1.17)	**0.0001**
Lymphotoxin-alpha/TNF-beta	0.07 (−0.03, 0.17)	0.1562	—	—	0.14 (−0.26, 0.54)	0.4867	—	—	0.34 (0.02, 0.67)	**0.0392**	0.30 (0.02, 0.59)	**0.0375**
IL-13^a^	−0.15 (−0.30, 0.01)	0.0592	—	—	−0.11 (−0.41, 0.19)	0.4637	—	—	−0.43 (−0.77, −0.09)	**0.0144**	—	—
IL-5^a^	−0.13 (−0.47, 0.22)	0.4750	—	—	0.11 (−1.39, 1.60)	0.8886	—	—	−5.61 (−11.00, −0.22)	**0.0414**	—	—
IL-12p70	−0.03 (−0.11, 0.05)	0.4401	—	—	0.04 (−0.12, 0.19)	0.6390	—	—	−0.02 (−0.21, 0.17)	0.8456	—	—
CCL2/JE/MCP-1	−0.00 (−0.33, 0.32)	0.9826	—	—	0.25 (−0.42, 0.92)	0.4650	—	—	−0.71 (−1.73, 0.31)	0.1724	—	—
IL-15	−0.06 (−0.14, 0.01)	0.0828	—	—	0.01 (−0.11, 0.13)	0.8825	—	—	−0.07 (−0.23, 0.08)	0.3690	—	—
TNF-alpha	−0.11 (−0.24, 0.02)	0.0909	—	—	−0.02 (−0.22, 0.18)	0.8238	—	—	−0.50 (−0.83, −0.16)	**0.0042**	—	—
IL-28B/IFN-lambda3	0.09 (−0.11, 0.30)	0.3569	—	—	0.52 (−0.24, 1.27)	0.1781	—	—	0.26 (−0.25, 0.76)	0.3207	—	—
CD40Ligand/TNFSF5	−0.13 (−0.34, 0.07)	0.2077	—	—	0.11 (−0.35, 0.57)	0.6409	—	—	0.00 (−0.47, 0.47)	0.9892	—	—
IL-23^a^	0.02 (−0.09, 0.12)	0.7340	—	—	−0.14 (−0.40, 0.12)	0.2915	—	—	−0.10 (−0.36, 0.17)	0.4684	—	—
IL-18/IL-1F4	−0.01 (−0.33, 0.32)	0.9758	—	—	−0.27 (−0.94, 0.39)	0.4203	—	—	−0.88 (−1.71, −0.04)	**0.0395**	—	—
IL-28A/IFN-lambda2^a^	0.06 (−0.09, 0.21)	0.4207	—	—	−0.18 (−0.49, 0.12)	0.2417	—	—	0.08 (−0.20, 0.37)	0.5720	—	—

*Notes:* Categories with no statistically significant variables are not shown; full data are available in Supplemental Table 1, http://links.lww.com/HC9/A544. All cytokines are expressed as log10. Univariable general linear models were performed to study independent predictors of IgG Spike response. Covariates showing a clinical and statistical significance or participating as a confounding factor for the variable of interest were included in the final stepwise multivariable models.

* Includes autoimmune hepatitis, primary sclerosing cholangitis, and primary biliary cholangitis.

a Cytokines with more than 25% undetectable values are not included in the multivariable models.

Abbreviations: ALP, alkaline phosphatase; APRIL/TNFSF13, a proliferation-inducing ligand/TNF ligand superfamily member 13; BAFF/TNFSF13B, B-cell activating factor/TNF ligand superfamily member 13B; CCL2/MCP-1, chemokine ligand 2/monocyte chemoattractant protein 1; CCL3/MIP-1, chemokine ligand 3/macrophage inflammatory protein 1α; CCL4/MIP-1, chemokine ligand 4/macrophage inflammatory protein 1β; CD40L/TNFSF5, cluster of differentiation 40 ligand/TNF ligand superfamily member 5; COPD, Chronic obstructive pulmonary disease; CXCL10/IP-10, C-X-C motif chemokine ligand 10/interferon gamma-induced protein 10; IFN, IFNγ, interferon gamma; mRNA, messenger RNA; 6-MP, 6-mercaptopurine;CRF, chronic renal failure; CVD, cerebrovascular disease; DM, diabetes mellitus; E, estimate.

In patients with cirrhosis, younger age [estimate −0.01 (−0.03, −0.00), *p* = 0.0044], lower Child-Turcotte-Pugh class [estimate −0.23 (−0.45, −0.01) for class B + C, *p* = 0.0365], and lower IL-6 [estimate −0.10 (−0.18, −0.01), *p* = 0.0355] were independently associated with higher anti-Spike IgG response. In a subanalysis according to vaccine type, patients who received Moderna [n = 34; estimate 0.38 (0.14, 0.62, *p* = 0.0018)] showed higher anti-Spike IgG response compared with Pfizer-BioNTech (n = 213) (Supplemental Table 2, http://links.lww.com/HC9/A544).

In the AILD group, the only independent factor associated with higher anti-Spike IgG was nonviral vaccine type [estimate 0.47 (0.10, 0.84), *p* = 0.0128]. In a similar subanalysis according to vaccine type, patients who received Moderna [n = 13; estimate 0.67 (0.25, 1.09), *p* = 0.0022] showed higher anti-Spike IgG response, while those receiving Janssen [n = 5; estimate −0.93 (−1.58, −0.28), *p* = 0.0053] showed lower serological response compared with Pfizer-BioNTech (n = 66).

In the post-LT group, the independent associations with higher anti-Spike IgG response were the absence of coronary artery disease [estimate 1.08 (0.19, 1.96), *p* = 0.0177 for its absence], lack of MMF-based immunosuppression [estimate 0.78 (0.43, 1.13), *p* < 0.001 for lack of MMF treatment], lower levels of BAFF [estimate 2.34 (1.17, 3.51), *p* = 0.0001], and higher levels of lymphotoxin-α/TNFβ [estimate 0.30 (0.02, 0.59), *p* = 0.0375]. Cytokine data for each subgroup are shown in Supplemental Figure 4, http://links.lww.com/HC9/A544, and Table 2, http://links.lww.com/HC9/A544.

In the control group, the only independent predictor of anti-Spike IgG response was IL-13 (estimate –0.56 (−1.10, −0.03), *p* = 0.0387).

### Anti-SARS-CoV-2 neutralizing activity in patients with cirrhosis and AILD, and post-LT

**FIGURE 3:**
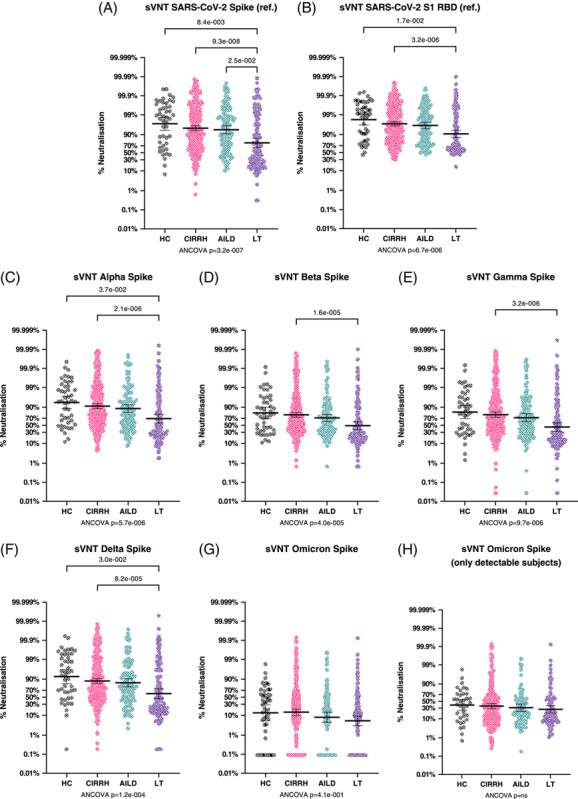
Anti-SARS-CoV-2 antibody neutralization potential measured by surrogate viral neutralization assay (sVNT). (A and B) Percentage neutralization achieved (PNA) against the reference strain Spike and RBD (respectively) by serum from healthy controls, patients with cirrhosis (CIRRH), autoimmune liver disease (AILD), and liver transplant (LT). (C–G) PNA against Alpha, Beta, Gamma, Delta, and Omicron Spike, respectively, by serum from the same 4 groups. (H) sensitivity analysis for Omicron PNA in the 4 subject groups after removing all subjects with Omicron PNA below the detection limit. All measurements were obtained using electrochemoluminescence-based meso scale discovery multiplex competitive binding assays. Samples were diluted at 1:12.5 for quantification, as recommended by the manufacturer. All data are represented on probability/percentage Logit scale scatterplot graphs; lines and error bars represent geometric mean and 95% CI. All comparisons were assessed by ANCOVA, adjusted for age, sex, and time of sample collection postvaccination, followed by Bonferroni-corrected pairwise comparisons between covariate-adjusted estimated group means. Abbreviations: AILD, autoimmune liver disease; CIRRH, cirrhosis; LT, liver transplant; PNA, percentage neutralization achieved; sVNT, surrogate viral neutralization assay; RBD, receptor binding domain.

Neutralizing activity of anti-SARS-CoV-2 antibodies was conducted in 577 patients, and it was assessed by live virus microneutralization assay against reference strain, as well as strain-specific neutralization using a surrogate ACE2 binding assay. Live virus microneutralization data for each patient group are shown in Supplemental Figure 5, http://links.lww.com/HC9/A544. The surrogate ACE2 binding assay was validated by correlation with live virus microneutralization; assays were highly correlated for the reference strain, *r* ^*2*^ = 0.887, *p* < 0.0001. Surrogate neutralizing activity for each variant of concern (VOC), by patient group, is shown in Figure 3. Similar to anti-Spike IgG data, the anti-SARS-CoV-2 surrogate neutralizing activity was lowest in the post-LT group, and this reached significance against each of the other disease groups for the anti-Spike reference strain but only against control and cirrhosis groups for anti-RBD reference, Alpha and Delta strains, and against control for Beta and Gamma strains. A subject-matched comparison between different viral strains is represented in Supplemental Figure 6, http://links.lww.com/HC9/A544. No significant between-group differences were noted for Omicron; however, surrogate Omicron neutralization was consistently lower across all disease groups compared with other strains.

### Factors associated with breakthrough SARS-CoV-2 infection in patients with cirrhosis and AILD, and post-LT

Participants were followed up for breakthrough infection until the third vaccine dose (booster) or 8 months after the second vaccine dose, whichever was sooner. Follow-up data were available from 598 participants from Italy and Spain (305 cirrhosis, 113 AILD, 135 post-LT, and 45 control). The exact duration of the third vaccine dose was available for 106 participants; among these, the median duration between second and third doses was 183 days (p25–p75: 142–204 d). Symptomatic breakthrough infections occurred in 20 patients (6.6%) in the cirrhosis group, 12 patients (10.6%) in the AILD group, 10 patients (7.4%) in the post-LT group, and 7 control (15.6%); these differences did not attain statistical significance in pairwise comparisons. Of these, 1 patient from the cirrhosis group (1/20, 5%) required hospitalization for respiratory symptoms and went on to require invasive ventilation, and 1 patient from the LT group (1/10, 10%) also required hospitalization for respiratory symptoms; no other hospitalizations were recorded.

**FIGURE 4:**
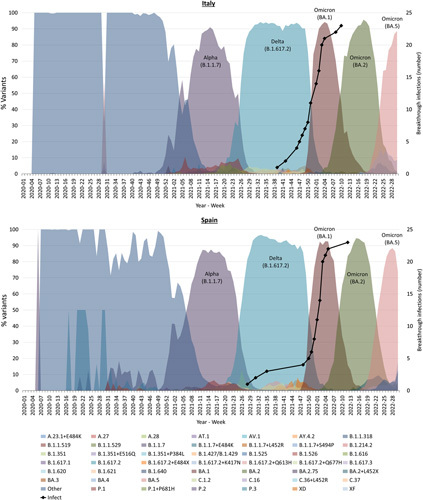
Breakthrough infections by country. The top graph illustrates breakthrough infections recorded in the Italian cohort (black line) longitudinally represented on the background of the main viral variants sequenced over time in Italy. The bottom graph is similar in relation to the Spanish cohort and main viral variants sequenced in Spain. Longitudinal country-specific viral variant data were downloaded from the ECDC website (European Centre for Disease Prevention and Control). No specific clusters of breakthrough infections were observed based on the subject group, but subjects from all 4 groups (healthy controls, cirrhosis, autoimmune liver disease, and liver transplant) were comparably spread along the infection timeline. The Spanish breakthrough infection cohort did not contain any healthy controls. Available data on the volume of COVID-19 sequencing, the number and percentage distribution of VOC for each country, week, and variant submitted since 2020-W40 to the GISAID EpiCoV database (https://www.gisaid.org/) and TESSy (as either case-based or aggregate data). Abbreviation: ECDC, European Centre for Disease Prevention and Control.

Characteristics of patients with and without breakthrough SARS-CoV-2 infection are shown in Supplemental Table 3, http://links.lww.com/HC9/A544. The timeline of infections is presented in Figure 4 as a schematic, with the predominant VOC in the relevant country also displayed; breakthrough infections occurred between July 2021 and March 2022, which were large periods of Delta or Omicron strain predominance.

**FIGURE 5:**
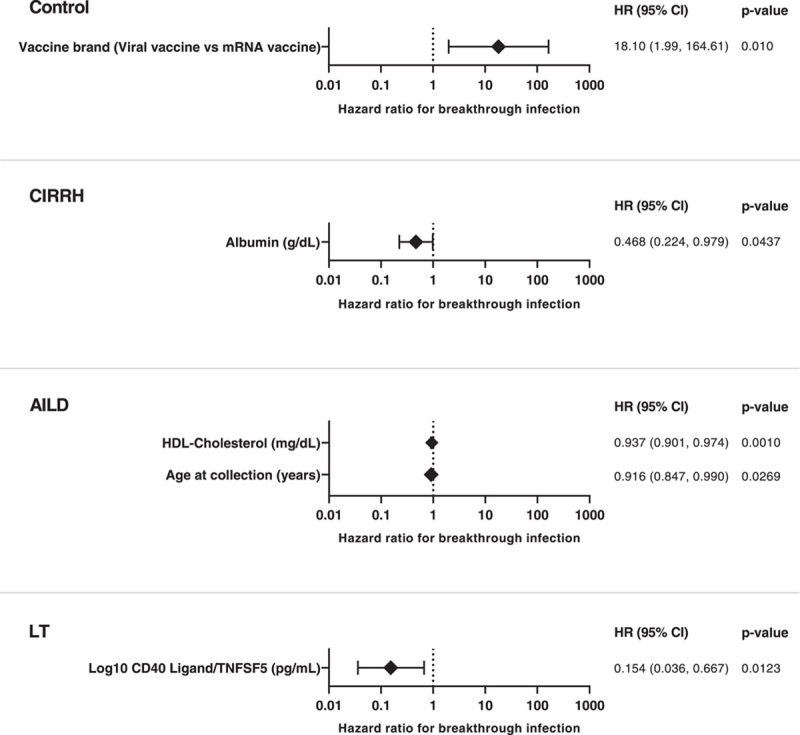
Cox regression analysis to identify demographic, clinical, serological, or immunological factors associated with high risk of breakthrough infection. The forest plot represents the HRs for factors associated with breakthrough infection, compared to no infection, in patients with cirrhosis (CIRRH) (A), autoimmune liver disease (AILD) (B), and liver transplant (LT) (C). Only factors with Cox regression coefficients significant at alpha = 0.05 in the multivariable analysis are represented in decreasing order of significance. Error bars represent the 95% CI for the respective HRs. The dotted black line marks HR=1, that is, no association. Abbreviations: AILD, autoimmune liver disease; CIRRH, cirrhosis; LT, liver transplant.

A Cox proportional hazard analysis was conducted in each disease group to evaluate risk factors for breakthrough infection; these data are presented in Table 4 (abbreviated data, full data in Supplemental Table 4, http://links.lww.com/HC9/A544) and Figure 5. In patients with cirrhosis, on multivariate analysis, the risk of breakthrough infection was independently associated with lower albumin concentration (HR 0.47, 95% CI: 0.2-1.0, *p* = 0.0437). Additionally, higher surrogate neutralizing capacity against the Omicron strain was noted to have borderline significance for the risk of breakthrough infection (HR 1.18, 95% CI: 1.0-1.4, *p* = 0.0559). In the AILD group, the risk of breakthrough infection was independently associated with older age (HR 0.92, 95% CI: 0.8-1.0, *p* = 0.0269) and lower HDL-cholesterol (HR 0.94, 95% CI: 0.9-1.0, *p* = 0.0010). In the post-LT group, the only significant factor associated with breakthrough infection was the level of cluster of differentiation 40 ligand, which was protective against breakthrough infection (HR 0.15, 95% CI: 0.0-0.7, *p* = 0.0123). Finally, in the control group, the only significant association with breakthrough infection was viral vaccine type (heterologous combination HR 17.58, 95% CI: 1.1-286.1, *p* = 0.0440, viral vaccine HR 14.57 1.7-126.3, *p* = 0.0150 compared Pfizer-BioNTech).

**TABLE 4:** Univariable and multivariable analyses for breakthrough infection

	Cirrhosis				Autoimmune liver disease				Liver transplant			
	Univariable		Multivariable		Univariable		Multivariable		Univariable		Multivariable	
Parameter	HR (95% CI)	*p*	HR (95% CI)	*p*	HR (95% CI)	*p*	HR (95% CI)	*p*	HR (95% CI)	*p*	HR (95% CI)	*p*
Age	0.97 (0.9, 1.0)	0.1679	—	—	0.95 (0.9, 0.99)	**0.0468**	0.92 (0.8, 1.0)	**0.0216**	1.07 (0.9, 1.2)	0.1125	—	—
Alcohol consumption												
No	Ref.	—	—	—	Ref.	—	—	—	Ref.	—	—	—
Former drinker	1.20 (0.4, 3.6)	0.7457	—	—	—	—	—	—	6.27 (1.5, 25.7)	**0.0108**	—	—
Current drinker	1.57 (0.4, 6.1)	0.9175	—	—	—	—	—	—	6.11 (0.7, 54.8)	0.1057	—	—
Etiology												
Alcohol	1.45 (0.6, 3.6)	0.4172	—	—	—	—	—	—	—	—	—	—
Viral	1.25 (0.5, 3.1)	0.6259	—	—	NA	—	—	—	NA	—	—	—
NAFLD	2.90 (1.1, 7.7)	**0.0316**	—	—	—	—	—	—	—	—	—	—
Autoimmune^a^	—	—	—	—	—	—	—	—	—	—	—	—
Other	1.05 (0.2, 4.6)	0.9511	—	—	—	—	—	—	—	—	—	—
Years since diagnosis												
<1	0.96 (0.2, 4.4)	0.9616	—	—	—	—	—	—	—	—	—	—
1--5	0.56 (0.2, 2.0)	0.3754	—	—	10.8 (1.1, 105)	**0.0408**	—	—	0.73 (0.1, 6.1)	0.7713	—	—
>5	Ref.	—	—	—	Ref.	—	—	—	Ref.		—	—
Comorbidities												
COPD	4.73 (1.3, 16.7)	**0.0159**	—	—	—	—	—	—	—	—	—	—
Heart failure	—	—	—	—	—	—	—	—	—	—	—	—
Hypertension	1.06 (0.4, 2.9)	0.9136	—	—	1.71 (0.4, 8.3)	0.5031	—	—	0.40 (0.1, 1.6)	0.1976	—	—
Coronary disease	2.85 (0.6-13.4)	0.1861	—	—	7.39 (0.9, 60.2)	0.0617	—	—	3.24 (0.4, 26.1)	0.269	—	—
CRF	—	—	—	—	—	—	—	—	3.41 (0.7, 16.5)	0.1262	—	—
CVD	0.84 (0.1, 6.4)	0.8668	—	—	—	—	—	—	—	—	—	—
DM	1.10 (0.4, 3.4)	0.8727	—	—	—	—	—	—	0.22 (0.0, 1.8)	0.1543	—	—
Psychiatric	2.73 (0.8, 9.7)	0.1197	—	—	—	—	—	—	—	—	—	—
Other disease	1.23 (0.4, 3.4)	0.6896	—	—	0.60 (0.1, 2.8)	0.5066	—	—	3.17 (0.9, 11.8)	0.0857	—	—
Laboratory												
Albumin	0.48 (0.3, 0.9)	**0.0168**	0.47 (0.2, 1.0)	**0.0437**	0.87 (0.1, 6.1)	0.8896	—	—	0.79 (0.2, 3.8)	0.7666	—	—
AST	1.01 (0.9, 1.02)	0.4105	—	—	1.01 (0.9, 1.0)	0.4967	—	—	0.98 (0.9, 1.0)	0.5477	—	—
ALT	1.01 (0.9, 1.02)	0.3825	—	—	1.00 (0.9, 1.0)	0.6848	—	—	0.99 (0.9, 1.0)	0.751	—	—
ALP	1.00 (0.9, 1.01)	0.985	—	—	0.99 (0.9, 1.0)	0.4089	—	—	1.00 (0.9, 1.0)	0.5707	—	—
GGT	1.00 (0.9, 1.01)	0.699	—	—	0.99 (0.9, 1.0)	0.3072	—	—	1.00 (0.9, 1.0)	0.3539	—	—
Total Bilirubin	1.03 (0.9, 1.2)	0.6844	—	—	0.59 (0.1, 5.1)	0.6355	—	—	1.69 (0.8, 3.4)	0.1421	—	—
Creatinine	1.48 (0.2, 9.6)	0.6846	—	—	0.16 (0.0, 8.9)	0.3692	—	—	0.61 (0.1, 5.7)	0.6687	—	—
Sodium	0.93 (0.8, 1.1)	0.2898	—	—	0.92 (0.7, 1.2)	0.5049	—	—	0.98 (0.7, 1.3)	0.9018	—	—
Total cholesterol	0.99 (0.9, 1.0)	0.1108	—	—	1.00 (0.9, 1.0)	0.8985	—	—	1.00 (0.9, 1.0)	0.7945	—	—
HDL-Cholesterol	0.98 (0.9, 1.0)	0.3887	—	—	0.96 (0.9, 0.99)	**0.0041**	0.94 (0.9-1.0)	**0.0031**	1.04 (0.9, 1.2)	0.5556	—	—
LDL-Cholesterol	0.99 (0.9, 1.0)	0.6263	—	—	0.99 (0.9, 1.0)	0.6967	—	—	1.01 (0.9, 1.1)	0.9308	—	—
Triglycerides	0.99 (0.9, 1.0)	0.7565	—	—	0.99 (0.9, 1.0)	0.44	—	—	0.99 (0.9, 1.0)	0.3804	—	—
C-reactive protein	1.00 (0.9, 1.0)	0.2038	—	—	---	---	—	—	1.02 (0.9, 1.1)	0.3752	—	—
Hemoglobin	1.08 (0.9, 1.3)	0.3414	—	—	0.68 (0.5, 0.9)	**0.004**	—	—	0.83 (0.6, 1.1)	0.2526	—	—
Leukocyte	0.91 (0.7, 1.2)	0.4353	—	—	0.99 (0.8, 1.3)	0.9681	—	—	0.95 (0.7, 1.2)	0.6729	—	—
Lymphocytes	0.66 (0.3, 1.4)	0.2623	—	—	1.06 (0.5, 2.2)	0.8829	—	—	0.97 (0.6, 1.6)	0.9126	—	—
Monocytes	1.27 (0.4, 3.8)	0.6772	—	—	0.24 (0.0, 24.1)	0.5397	—	—	0.05 (0.0, 69.1)	0.4125	—	—
Neutrophils	0.88 (0.6, 1.3)	0.5055	—	—	1.11 (0.7, 1.7)	0.6319	—	—	0.93 (0.7, 1.3)	0.7071	—	—
Neutrophil/Lymphocyte	1.01 (0.9, 1.0)	0.7052	—	—	2.29 (0.9, 6.1)	0.0955	—	—	1.08 (0.5, 2.5)	0.848	—	—
Platelet	0.99 (1.0, 1.0)	0.0539	—	—	1.00 (0.9, 1.0)	0.7698	—	—	1.00 (0.9, 1.0)	0.9078	—	—
INR	1.47 (0.4, 6.1)	0.5942	—	—	0.30 (0.0, 48.6)	0.6409	—	—	0.62 (0.1, 3.7)	0.5965	—	—
Prothrombin time	—	—	—	—	1.27 (0.5-3.1)	0.5914	—	—	—	—	—	—
Cytokines												
IL-6	1.23 (0.8, 1.8)	0.3027	—	—	0.47 (0.1, 1.5)	0.2156	—	—	0.86 (0.4, 1.7)	0.6562	—	—
IL-33^b^	1.10 (0.8, 1.5)	0.5506	—	—	—	—	—	—	—	—	—	—
IL-8/CXCL8	2.10 (0.8, 5.6)	0.1345	—	—	0.89 (0.4, 2.1)	0.7935	—	—	0.72 (0.2, 2.2)	0.5706	—	—
CXCL10/IP-10/CRG-2	3.02 (0.8, 11.8)	0.111	—	—	1.93 (0.2, 21.1)	0.5895	—	—	1.66 (0.3, 10.2)	0.586	—	—
IL-10^b^	0.35 (0.1, 0.9)	**0.0267**	—	—	0.52 (0.2, 1.5)	0.2258	—	—	0.66 (0.2, 1.8)	0.4039	—	—
IL-27	0.93 (0.6, 1.4)	0.7193	—	—	1.46 (0.8, 2.6)	0.2102	—	—	0.87 (0.5, 1.7)	0.672	—	—
IL-2^b^	1.65 (1.0, 2.7)	**0.0432**	—	—	1.29 (0.7, 2.3)	0.3688	—	—	1.21 (0.6, 2.3)	0.5645	—	—
IFN-gamma	1.04 (0.8, 1.3)	0.7627	—	—	0.92 (0.6, 1.3)	0.64	—	—	0.77 (0.4, 1.4)	0.3862	—	—
IL-1ra/IL-1F3	1.32 (0.2, 7.0)	0.7439	—	—	0.13 (0.0, 2.2)	0.1585	—	—	0.08 (0.0, 1.1)	0.0617	—	—
CCL3/MIP-1 alpha^b^	—	—	—	—	—	—	—	—	—	—	—	—
CCL4/MIP-1 beta	0.40 (0.1, 1.1)	0.0883	—	—	0.89 (0.2, 3.5)	0.8642	—	—	0.53 (0.1, 2.1)	0.3609	—	—
IL-1 alpha/IL-1F1^b^	—	—	—	—	—	—	—	—	—	—	—	—
IL-4^b^	1.06 (0.5, 2.2)	0.874	—	—	0.56 (0.1-2.4)	0.4389	—	—	0.77 (0.2, 3.4)	0.7334	—	—
IL-17/IL-17A	0.939 (0.5, 1.6)	0.8249	—	—	—	—	—	—	1.02 (0.5, 2.2)	0.9527	—	—
APRIL/TNFSF13	0.76 (0.1, 5.6)	0.786	—	—	0.74 (0.0-62.9)	0.8954	—	—	0.14 (0.0, 1.5)	0.1016	—	—
BAFF/BLyS/TNFSF13B	4.89 (0.9, 27.4)	0.0709	—	—	0.32 (0.0, 34.2)	0.6301	—	—	0.75 (0.0, 57.4)	0.8959	—	—
Lymphotoxin-alpha/TNF-beta	0.89 (0.5, 1.7)	0.7198	—	—	—	—	—	—	—	—	—	—
IL-13^b^	0.31 (0.1, 1.6)	0.1623	—	—	0.90 (0.3, 2.7)	0.8477	—	—	1.34 (0.4, 4.1)	0.6073	—	—
IL-5^b^	—	—	—	—	—	—	—	—	—	—	—	—
IL-12p70	1.06 (0.7, 1.6)	0.7792	—	—	1.51 (0.9, 2.6)	0.1248	—	—	1.06 (0.5, 2.1)	0.8591	—	—
CCL2/JE/MCP-1	1.29 (0.2, 8.0)	0.782	—	—	0.40 (0.0, 6.0)	0.5104	—	—	0.81 (0.0, 19.9)	0.8958	—	—
IL-15	1.23 (0.8, 1.8)	0.3032	—	—	1.39 (0.9, 2.2)	0.1789	—	—	1.17 (0.7, 2.0)	0.54	—	—
TNF-alpha	1.37 (0.6, 3.2)	0.4796	—	—	0.60 (0.4, 1.3)	0.2665	—	—	0.87 (0.2-3.8)	0.8569	—	—
IL-28B/IFN-lambda3	0.34 (0.1, 1.9)	0.2178	—	—	—	—	—	—	—	—	—	—
CD40Ligand/TNFSF5	0.83 (0.3, 1.6)	0.753	—	—	0.78 (0.2, 3.2)	0.7333	—	—	0.15 (0.0, 0.7)	**0.0123**	0.15 (0.0, 0.7)	**0.0123**
IL-23^b^	1.02 (0.6, 1.9)	0.9406	—	—	0.54 (0.1, 3.1)	0.488	—	—	—	—	—	—
IL-18/IL-1F4	3.90 (0.7, 22.7)	0.1303	—	—	2.78 (0.3, 28.2)	0.3884	—	—	0.98 (0.0, 27.9)	0.9891	—	—
IL-28A/IFN-lambda2^b^	1.47 (0.7, 3.3)	0.3466	—	—	2.88 (1.0, 8.7)	0.0609	—	—	1.63 (0.6, 4.1)	0.2985	—	—
Anti-SARS-CoV-2 antibodies												
IgG SARS-CoV-2 Spike	1.22 (0.7, 2.3)	0.537	—	—	1.63 (0.5, 5.7)	0.4442	—	—	0.55 (0.3, 1.2)	0.1229	—	—
IgG/IgM ratio SARS-CoV-2 Spike	0.69 (0.3, 1.4)	0.2969	—	—	0.49 (0.2, 1.3)	0.1636	—	—	0.58 (0.2, 1.6)	0.2812	—	—
Neutr. Spike Reference Strain	1.13 (0.9, 1.3)	0.1718	—	—	1.16 (0.9, 1.5)	0.2786	—	—	0.97 (0.8, 1.2)	0.8008	—	—
Neutr. S1 RBD Reference Strain	1.17 (0.9, 1.4)	0.153	—	—	1.25 (0.9, 1.8)	0.2233	—	—	0.96 (0.7, 1.4)	0.8364	—	—
Neutr. Alpha Spike	1.14 (0.9, 1.3)	0.1291	—	—	1.09 (0.8, 1.4)	0.4808	—	—	0.94 (0.7, 1.3)	0.676	—	—
Neutr. Beta Spike	1.17 (1.0, 1.4)	0.0503	—	—	1.06 (0.8, 1.4)	0.6808	—	—	0.92 (0.6, 1.3)	0.6367	—	—
Neutr. Gamma Spike	1.12 (0.9, 1.3)	0.1344	—	—	1.06 (0.8, 1.4)	0.6501	—	—	0.88 (0.6, 1.3)	0.5003	—	—
Neutr. Delta Spike	1.12 (0.9, 1.3)	0.1668	—	—	1.15 (0.9, 1.5)	0.2766	—	—	0.95 (0.7, 1.2)	0.7291	—	—
Neutr. Omicron Spike	1.25 (1.1, 1.5)	**0.0082**	1.18 (1.0, 1.4)	0.0559	0.95 (0.7, 1.3)	0.7408	—	—	0.98 (0.7, 1.4)	0.9288	—	—

*Notes:* Categories with no statistically significant variables not shown; full data available in supplemental Table 3, http://links.lww.com/HC9/A544. All cytokines, IgG, and IgM are expressed as log10. Neutralization is expressed as Logit %.

Cox proportional hazard models were used to study independent predictors of breakthrough infection. Independent covariates were included in the models when showing statistical significance or confounding. Proportional hazard assumptions were explored by testing zph based on the weighted Schoenfeld, and PH assumptions were met for all variables included in the models.

a Includes autoimmune hepatitis, primary sclerosing cholangitis, and primary biliary cholangitis.

b Cytokines with more than 25% undetectable values. These cytokines are not included in the multivariable models.

Abbreviations: MP, 6-mercaptopurine; ALP, alkaline phosphatase; BAFF, B-cell activating factor; TNFSF13B, TNF ligand superfamily member 13; CCL2/MCP-1, chemokine ligand 2/monocyte chemoattractant protein 1; CCL3, chemokine ligand 3/macrophage inflammatory protein 1α; CCL4/MIP-1, chemokine ligand 4/macrophage inflammatory protein 1β; CD40L/TNFSF5, cluster of differentiation 40 ligand/TNF ligand superfamily member 5; CVD, cerebrovascular disease; COVD, chronic obstructive pulmonary disease; CRF, chronic renal failure; CXCL10/IP-10, C-X-C motif chemokine ligand 10/interferon gamma-induced protein 10; DM, diabetes mellitus; E, estimate; neutr, neutralization; PH, proportional hazard; RBD, receptor binding domain.

## DISCUSSION

This study provides data on vaccine response and risk of breakthrough infection following initial COVID-19 vaccination in 1 of the largest reported cohorts of patients with cirrhosis or AILD. Although many developed countries have moved on to booster dosing regimens, many patients remain unboosted due to vaccine hesitancy and lack of access, and the data presented here have particular relevance for this patient group. These data also augment the existing literature for LT recipients and provide novel data regarding neutralizing activity. There was no statistically significant reduction in humoral response or neutralizing activity in patients with cirrhosis, but the vaccine responses were very variable within this group and breakthrough infections occurred with a higher incidence than reported in other cohorts.

Among patients with cirrhosis, deficient vaccine response was, in part, associated with factors known to be associated with decreased COVID-19 vaccine immunogenicity, such as age and disease severity. However, these data support a novel association of systemic inflammation, represented by elevated IL-6, with decreased vaccine response in cirrhosis. Chronic inflammation has been demonstrated to impair vaccine responses to other viral infections,^17–19^ and targeting inflammation has been suggested as a means to augment vaccine response, but inflammation has not been previously associated with vaccine response in liver disease.^20^ There are several roles for IL-6 in response to acute infection or tissue injury, relating to the acute phase response and tissue repair.^21^ Indeed, IL-6 is associated with B-cell differentiation and positively correlated with serological vaccine response in some settings.^22^ However, we believe that the data presented here are more in keeping with the chronic state of antigen stimulation in cirrhosis, as a consequence of gut-derived translocation of bacterial products. Several investigators have reported impaired vaccine response in the context of chronic background inflammation, which has been attributed to inflammation.^23–26^ Therefore, although further work is required to delineate mechanisms, these data are consistent with the growing appreciation of chronic inflammation as being a dominant factor influencing the risk of liver-related complications in cirrhosis.

Patients with AILD had comparable vaccine responses to control, and the major variable associated with humoral response was mRNA vaccine type as reported for other populations. Of note, the Janssen adenoviral vaccine showed a lower IgG Spike response compared to Pfizer-BioNTech, which may be due to the fact that the initial vaccine regimen included only 1 dose as opposed to Pfizer’s 2-dose regimen. Additionally, immunosuppression was not a factor associated with vaccine response in this group although less than half were taking immunosuppression and only 8% were receiving mycophenolate, which has previously been associated with impaired vaccine response. By contrast, the post-LT population had consistently lower responses to COVID-19 vaccination, in terms of both humoral response and SARS-CoV-2 neutralizing activity. These data are consistent with other reports in this population, in particular an independent, negative association with mycophenolate use.^27–31^ The novel findings in this cohort are additional independent associations with the presence of comorbid cardiovascular disease, which, although noted in nontransplant populations, has not been reported post-LT.^32^ Additionally, dysregulation of BAFF and lymphotoxin-α were found to be independently associated with response in this group. The negative association with BAFF and lymphotoxin-α may reflect the induction of regulatory B cells and reduced inflammatory responses, respectively, which together act to suppress immune responses.^33^

Consistent with existing data, a surrogate marker for neutralization activity decreased for VOCs relative to the reference strain. Decreasing immunogenicity of VOCs and potential immune escape has been a public health concern since the alpha (B.1.1.7) VOC was reported in November 2020. Subsequent VOCs have shown greater resistance to neutralization with accumulating mutations in the Spike protein; indeed, the most recent Omicron subvariant (BA.4/5) is markedly resistant to neutralization by sera from vaccinated individuals^34^ and currently accounts for the majority of new infections. Although strain-specific nAbs were not directly measured for each VOC in this study, which is a limitation of the data presented, the assay used represents the ability of participant serum to impede binding between ACE2 and strain-specific RBD domains and is, therefore, a surrogate for strain-specific neutralization. Moreover, the assay was validated against “live virus” neutralization. Consequently, data from his cohort are consistent with data from other conditions demonstrating lower neutralization for Omicron compared to other strains across all disease groups.

Breakthrough infections occurred in all groups, with rates between ~6% and 10% in the disease groups that are higher than reported in other disease cohorts.^35^ The rate of breakthrough infection showed a trend towards a higher rate in the control group although this may relate to different behavior in nonshielding populations. In the post-LT group, the finding of CD40 ligand being protective against breakthrough infection is novel. Of note, cluster of differentiation 40 ligand is involved in B-cell proliferation, and immunoglobulin class switching, antibody secretion, and CD40 agonists are currently being used to expand B cells for cancer immunotherapy.^36^ In the AILD group, the independent associations with breakthrough infection were older age and lower HDL-cholesterol levels; the association between HDL levels and susceptibility to COVID-19 has been previously observed.^37^

Within the cirrhosis cohort, the only significant independent association with breakthrough infection was lower serum albumin concentration. The finding is consistent with the well-established favorable immunomodulatory role of albumin in cirrhosis.^38^ The significance of the borderline inverse association between surrogate Omicron neutralization capacity and breakthrough infection is uncertain.

The results of this study should be interpreted in light of some of the limitations. First, due to the speed of the vaccine roll-out, we were unable to acquire baseline (prevaccine) samples to measure dynamic changes in immune response. However, participants with prior COVID-19 were excluded, and therefore, we do not anticipate previous infection confounding the data shown here. Second, for the same reason, peripheral blood mononuclear cells were not sampled, which prevents any detailed analysis of cellular immune response to vaccination. Data on cellular immune response to COVID-19 vaccination in cirrhosis are emerging and suggest impaired T-cell response^39^. Third, although we have accounted for many demographic and biological variables in multivariable analyses of vaccine response and infection risk, other major factors influencing breakthrough infection include local prevalence of infection, viral load exposure, and shielding behavior. Although we were unable to account for these factors in this study, we anticipate shielding behavior to be relatively consistent within disease groups, and consequently, we conducted within-group comparisons. To limit the impact of geographic variation in SARS-CoV-2 infection rates, follow-up data were collected from Italy and Spain only. Nevertheless, the relatively small number of infections means that these data should be interpreted cautiously. Fourth, asymptomatic SARS-CoV-2 breakthrough infection during follow-up was not evaluated, which may have introduced some bias on the subsequent analysis of vaccine efficacy; nonetheless, symptomatic COVID-19 was reliably assessed through telephone contact with the patient and not just by reviewing medical records (that may not have reflected a home antigen positive test). Fifth, participants were followed up for breakthrough infection until 8 months or administration of the third vaccine dose, whichever was sooner. Although the data collection process was robust, the exact date of third dose administration is only available in a subset but is likely to be similar across the cohort given the nature of the booster roll-out. Finally, although a relatively large number of breakthrough infections were reported, we were unable to confirm, by molecular testing, the strain of each infection. Therefore, we have compared dates of infection with dominant strains prevalent at the time in each geographic region, confirming that most infections were due to Delta or Omicron.

In summary, the data presented here demonstrate heterogeneous COVID-19 vaccine response, following the initial vaccination course, in patients with cirrhosis, with no clear correlate of protection. Poor vaccine response was associated with older age, disease severity, and systemic inflammation. Symptomatic breakthrough infection in this group was also associated with lower serum albumin. Post-LT patients were found to have consistently lower anti-SARS-CoV-2 humoral responses following initial vaccination, associated with mycophenolate-based immunosuppression and concomitant cardiovascular disease. These data provide a basis to target booster vaccination programs to higher risk patients, particularly in areas of the world where access to vaccination remains a barrier (such as Africa and parts of central Asia).^2^ Additionally, the data may be used to counter vaccine hesitancy in high-risk patients. Secondly, these data support a personalized approach to vaccine dosing in patients with cirrhosis. Specifically, augmented vaccination or booster regimens may be required in older patients or those with Child-Turcotte-Pugh B or C disease.

## Supplementary Material
