# Supplementary material for: Serological response and breakthrough infection after COVID-19 vaccination in patients with cirrhosis and post-liver transplant
Source: Hepatol Commun. 2023 Oct 18;7(11):e0273. doi: 10.1097/HC9.0000000000000273 (PMC10586829; doi:10.1097/HC9.0000000000000273)
Supplement: Supplementary file 1 [file hc9-7-e0273-s001.pdf]

Supplementary Files and Data

Serological Response and Breakthrough Infection After Initial Covid-19 Vaccination in Cirrhosis and Liver Transplant Patients

Gautam Mehta*, Antonio Riva*, Maria Pilar Ballester*, Eva Uson*, et al. on behalf of the COBALT Consortium

1. **Supplementary Tables 1-4**
2. **Supplementary Figures 1-6**
3. **Ethical Approvals**
4. **Study Protocol**
5. **Statistical Plan**

Supplementary Table 1. Univariable and multivariable sensitivity analyses of IgG Spike response in patients with nucleocapsid <5000.

	Cirrhosis				**Autoimmune liver disease**				**Liver transplant**			
	Univariable		Multivariable		**Univariable**		**Multivariable**		**Univariable**		**Multivariable**	
**Parameter**	E (95%CI)	p-value	E (95%CI)	p-value	**E (95%CI)**	**p-value**	**E (95%CI)**	**p-value**	**E (95%CI)**	**p-value**	**E (95%CI)**	**p-value**
Sex (female)	0.14 (-0.03, 0.32)	0.1075			0.06 (-0.30, 0.42)	0.7481			0.10 (-0.35, 0.55)	0.6714		
Age	-0.02 (-0.03, -0.01)	**<.0001**	-0.01 (-0.03, -0.00)	**0.0044**	-0.01 (-0.03, -0.00)	**0.0428**			-0.01 (-0.03, 0.01)	0.1828		
Race												
White	Ref.	0.1541			Ref.	**0.0462**				---		
Black or Afro-American	0.57 (-0.21, 1.35)				-1.52 (-3.02, -0.03)							
Ethnicity												
North European	-0.65 (-0.19, -0.12)	**0.0166**			-0.76 (-1.94, 0.41)	0.1997			-1.12 (-3.33, 1.10)	0.3191		
Mediterranean	-0.58 (-1.06, -0.10)	**0.0185**			-0.45 (-1.53, 0.64)	0.4152			-1.26 (-3.29, 0.77)	0.2207		
Latin American	-0.05 (-0.81, 0.71)	0.8958			-0.47 (-1.70, 0.77)	0.4574			---			
Other	Ref.				Ref.				Ref.			
Alcohol consumption												
No	Ref.				Ref.				Ref.			
Former drinker	-0.06 (-0.24, 0.13)	0.5566			-0.29 (-1.36, 0.79)	0.5940			-0.07 (-0.49, 0.35)	0.7529		
Current drinker	-0.13 (-0.40, 0.14)	0.3505			0.50 (-0.13, 1.13)	0.1210			-0.94 (-1.79, -0.09)	**0.0301**		
Tobacco consumption												
No	Ref.				Ref.				Ref.			
Former smoker	-0.07 (-0.26, 0.12)	0.4534			0.32 (-0.07, 0.70)	0.1097			-0.10 (-0.54, 0.34)	0.6474		
Current smoker	-0.12 (-0.39, 0.15)º	0.3741			-0.12 (-0.57, 0.33)	0.6010			-0.29 (-0.88, 0.29)	0.3222		
Aetiology												
Alcohol	0.02 (-0.15, 0.18)	0.8578										
Viral	-0.16 (-0.33, 0.00)	0.0568										
NAFLD	-0.30 (-0.50, -0.10)	**0.0036**			NA				NA			
Autoimmune*	-0.17 (-0.42, 0.09)	0.1954										
Other	0.37 (-0.01, 0.75)	0.0564										

Years since diagnosis												
<1	0.16 (-0.13, 0.45)	0.2844			-0.14 (-0.68, 0.41)	0.6241			---			
1-5	0.13 (-0.07, 0.34)	0.2062			-0.16 (-0.47, 0.16)	0.3278			-0.23 (-0.84, 0.38)	0.4517		
>5	Ref.				Ref.				Ref.			
History of acute decompensation	-0.07 (-0.26, 0.11)	0.4467			NA				-0.11 (-0.55, 0.32)	0.6038		
HCC	0.17 (-0.08, 0.41)	0.1769			---	---			-0.08 (-0.50, 0.34)	0.7117		
MELDNa week 7	-0.01 (-0.03, 0.01)	0.3254			NA				NA			
Child-Turcotte-Pugh class												
A	0.22 (0.05, 0.39)	**0.0123**	0.23 (0.01, 0.45)	**0.0365**	NA				NA			
B+C	Ref.											
Comorbidities												
COPD	-0.27 (-0.61, 0.08)	0.1293			-0.50 (-1.58, 0.58)	0.3639			0.43 (-0.49, 1.36)	0.3532		
Heart failure	-0.41 (-0.98, 0.16)	0.1543										
Hypertension	-0.11 (-0.29, 0.07)	0.2365			-0.09 (-0.47, 0.29)	0.6358			-0.08 (-0.49, 0.32)	0.6823		
Coronary disease	-0.05 (-0.35, 0.26)	0.7645			-0.39 (-1.27, 0.50)	0.3881			-1.04 (-2.05, -0.03)	**0.0441**	-1.08 (-1.96, -0.19)	**0.0177**
CRF	-0.32 (-0.71, 0.07)	0.1062			-0.02 (-1.11, 1.06)	0.9648			-0.04 (-0.74, 0.67)	0.9189		
CVD	-0.18 (-0.55, 0.19)	0.3331			0.19 (-1.34, 1.71)	0.8097			-0.12 (-1.15, 0.91)	0.8196		
DM	-0.21 (-0.41, -0.01)	**0.0417**			0.21 (-0.68, 1.10)	0.6359			-0.32 (-0.78, 0.14)	0.1705		
Psychiatric	-0.15 (-0.52, 0.23)	0.4497			0.45 (-0.44, 1.33)	0.3208			-0.54 (-1.56, 0.49)	0.3030		
Other disease	-0.01 (-0.20, 0.19)	0.9578			0.12 (-0.19, 0.43)	0.4408			-0.24 (-0.69, 0.21)	0.2961		
Laboratory												
Albumin	-0.01 (-0.12, 0.11)	0.9128			0.40 (-0.06, 0.87)	0.0897			0.21 (-0.34, 0.76)	0.4489		
AST	-0.00 (-0.00, 0.00)	0.1771			-0.00 (-0.01, 0.01)	0.9962			0.00 (-0.00, 0.01)	0.5602		
ALT	-0.00 (-0.00, 0.00)	0.2120			0.00 (-0.00, 0.00)	0.3279			0.00 (-0.00, 0.01)	0.3037		
ALP	-0.00 (-0.00, 0.00)	0.7740			-0.00 (-0.00, 0.00)	0.9062			-0.00 (-0.01, -0.00)	**0.0222**		
GGT	0.00 (-0.00, 0.00)	0.5749			0.00 (-0.00, 0.00)	0.1077			-0.00 (-0.00, -0.00)	**0.0140**		
Total Bilirubin	0.02 (-0.02, 0.05)	0.3426			-0.12 (-0.39, 0.16)	0.3985			0.05 (-0.25, 0.36)	0.5938		
Creatinine	-0.20 (-0.42, 0.02)	0.0753			-0.01 (-0.77, 0.75)	0.9829			-0.13 (-0.68, 0.42)	0.1582		
Sodium	0.02 (-0.01, 0.04)	0.1575			-0.03 (-0.10, 0.04)	0.4245			-0.01 (-0.03, 0.00)	0.1182		
Total cholesterol	-0.00 (-0.00, 0.00)	0.5762			-0.00 (-0.01, 0.00)	0.4716			0.00 (-0.01, 0.01)	0.7344		

HDL-Cholesterol	0.00 (-0.01, 0.01)	0.5118			-0.00 (-0.01, 0.01)	0.4349			0.01 (-0.01, 0.03)	0.4339		
LDL-Cholesterol	-0.00 (-0.00, 0.00)	0.7743			-0.00 (-0.01, 0.01)	0.8960			-0.00 (-0.01, 0.01)	0.3996		
Triglycerides	-0.00 (-0.00, 0.00)	0.5587			0.00 (-0.00, 0.01)	0.1612			-0.00 (-0.00, 0.00)	0.9674		
C-reactive protein	-0.00 (-0.00, 0.00)	0.3622			-0.06 (-0.40, 0.29)	0.6733			-0.01 (-0.03, -0.00)	**0.0438**		
Haemoglobin	0.03 (-0.00, 0.05)	0.0633			-0.01 (-0.07, 0.05)	0.7048			0.07 (-0.01, 0.16)	0.1036		
Leucocyte	0.00 (-0.00, 0.00)	0.8283			0.00 (-0.00, 0.00)	0.8272			0.06 (-0.01, 0.12)	0.0816		
Lymphocytes	0.10 (0.00, 0.20)	**0.0400**			0.00 (-0.20, 0.21)	0.9649			0.15 (-0.03, 0.33)	0.0983		
Monocytes	0.15 (-0.11, 0.42)	0.2566			-0.49 (-1.42, 0.44)	0.3010			0.73 (-0.57, 2.03)	0.2596		
Neutrophils	-0.05 (-0.10, 0.00)	0.0597			-0.02 (-0.14, 0.11)	0.8043			0.09 (-0.01, 0.20)	0.0853		
Neutrophil/Lymphocyte	0.01 (0.00, 0.01)	**0.0128**			0.00 (-0.01, 0.02)	0.7056			0.00 (-0.00, 0.01)	0.4235		
Platelet	-0.00 (-0.00, 0.00)	0.4440			-0.00 (-0.00, 0.00)	0.8196			-0.00 (-0.00, 0.00)	0.9572		
INR	-0.04 (-0.28, 0.20)	0.7705			-0.45 (-1.47, 0.56)	0.3777			0.08 (-1.02, 1.18)	0.8885		
Prothrombin time	0.01 (-0.14, 0.16)	0.9030			-0.25 (-0.81, 0.32)	0.3749			0.77 (-0.25, 1.80)	0.1350		
Immunosuppressive drugs	-0.22 (-0.70, 0.27)	0.3812			-0.09 (-0.40, 0.21)	0.5454			0.71 (-0.21, 1.63)	0.1272		
Steroids	-0.06 (-0.48, 0.36)	0.7816			-0.12 (-0.45, 0.21)	0.4694			-0.56 (-1.17, 0.05)	0.0714		
Calcineurin antagonist	-0.68 (-1.64, 0.27)	0.1616			0.23 (-0.46, 0.93)	0.5041			0.45 (-0.12, 1.03)	0.1205		
Mycophenolate	-0.28 (-1.24, 0.68)	0.5619			-0.15 (-0.68, 0.38)	0.5777			-0.72 (-1.09, -0.35)	**0.0002**	-0.78 (-1.13, -0.43)	**<.0001**
Azathioprine or 6-MP	0.06 (-0.62, 0.74)	0.8613			-0.12 (-0.44, 0.21)	0.4814			1.26 (-0.77, 3.28)	0.2211		
Other	---				0.29 (-1.23, 1.81)	0.7071			-0.19 (-0.79, 0.41)	0.5306		
Vaccine brand												
mRNA	Ref.				Ref.		Ref.		Ref.			
Viral	-0.52 (-0.92, -0.12)	**0.0107**			-0.47 (-0.84, -0.10)	**0.0128**	-0.47 (-0.84, -0.10)	0.0128	0.10 (-1.35, 1.55)	0.8913		
Heterologous									0.35 (-1.69, 2.39)	0.7325		
Cytokines												
IL-6	-0.11 (-0.18, -0.04)	0.0013	-0.10 (-0.18, -0.01)	**0.0355**	-0.15 (-0.33, 0.03)	0.0921			-0.15 (-0.33, 0.02)	0.0899		
IL-33**	0.01 (-0.06, 0.08)	0.7609			-0.03 (-0.18, 0.11)	0.6295			-0.00 (-0.24, 0.24)	0.9840		
IL-8/CXCL8	-0.02 (-0.17, 0.14)	0.8430			0.02 (-0.26, 0.30)	0.9029			-0.05 (-0.42, 0.31)	0.7716		
CXCL10/IP-10/CRG-2	-0.36 (-0.62, -0.10)	**0.0076**			-0.36 (-0.96, 0.23)	0.2297			-0.56 (-1.33, 0.20)	0.1465		
IL-10**	-0.08 (-0.17, 0.01)	0.0755			-0.06 (-0.23, 0.12)	0.5012			-0.10 (-0.34, 0.14)	0.4201		
IL-27	-0.00 (-0.07, 0.07)	0.9670			-0.17 (-0.35, 0.01)	0.0685			-0.05 (-0.24, 0.15)	0.6432		

IL-2**	0.02 (-0.06, 0.11)	0.5724			0.03 (-0.14, 0.20)	0.7220			0.08 (-0.11, 0.27)	0.4153		
IFN-gamma	0.02 (-0.03, 0.07)	0.3772			0.01 (-0.08, 0.10)	0.8025			0.12 (-0.04, 0.28)	0.1312		
IL-1ra/IL-1F3	0.13 (-0.19, 0.45)	0.4194			-0.13 (-0.86, 0.59)	0.7158			-0.02 (-0.80, 0.76)	0.9590		
CCL3/MIP-1 alpha**	0.19 (-0.32, 0.70)	0.4732			0.23 (-0.87, 1.33)	0.6810			-0.24 (-1.16, 0.69)	0.6149		
CCL4/MIP-1 beta	-0.05 (-0.24, 0.15)	0.6390			0.08 (-0.29, 0.44)	0.6833			-0.10 (-0.50, 0.30)	0.6315		
IL-1 alpha/IL-1F1**	-0.09 (-0.41, 0.22)	0.5643			---				-0.16 (-1.30, 0.98)	0.7837		
IL-4**	-0.06 (-0.20, 0.07)	0.3694			0.00 (-0.28, 0.29)	0.9780			-0.07 (-0.47, 0.34)	0.7412		
IL-17/IL-17A	0.01 (-0.08, 0.11)	0.7599			-0.04 (-0.33, 0.24)	0.7560			0.24 (0.02, 0.46)	**0.0308**		
APRIL/TNFSF13	0.02 (-0.32, 0.37)	0.8880			-0.10 (-1.18, 0.97)	0.8530			0.48 (-0.43, 1.38)	0.2996		
BAFF/BLyS/TNFSF13B	-0.23 (-0.63, 0.17)	0.2676			-0.12 (-1.28, 1.04)	0.8381			-2.58 (-3.83, -1.33)	**<.0001**	-2.34 (-3.51, -1.17)	**0.0001**
Lymphotoxin-alpha/TNF-beta	0.07 (-0.03, 0.17)	0.1562			0.14 (-0.26, 0.54)	0.4867			0.34 (0.02, 0.67)	**0.0392**	0.30 (0.02, 0.59)	**0.0375**
IL-13**	-0.15 (-0.30, 0.01)	0.0592			-0.11 (-0.41, 0.19)	0.4637			-0.43 (-0.77, -0.09)	**0.0144**		
IL-5**	-0.13 (-0.47, 0.22)	0.4750			0.11 (-1.39, 1.60)	0.8886			-5.61 (-11.00, -0.22)	**0.0414**		
IL-12p70	-0.03 (-0.11, 0.05)	0.4401			0.04 (-0.12, 0.19)	0.6390			-0.02 (-0.21, 0.17)	0.8456		
CCL2/JE/MCP-1	-0.00 (-0.33, 0.32)	0.9826			0.25 (-0.42, 0.92)	0.4650			-0.71 (-1.73, 0.31)	0.1724		
IL-15	-0.06 (-0.14, 0.01)	0.0828			0.01 (-0.11, 0.13)	0.8825			-0.07 (-0.23, 0.08)	0.3690		
TNF-alpha	-0.11 (-0.24, 0.02)	0.0909			-0.02 (-0.22, 0.18)	0.8238			-0.50 (-0.83, -0.16)	**0.0042**		
IL-28B/IFN-lambda3	0.09 (-0.11, 0.30)	0.3569			0.52 (-0.24, 1.27)	0.1781			0.26 (-0.25, 0.76)	0.3207		
CD40Ligand/TNFSF5	-0.13 (-0.34, 0.07)	0.2077			0.11 (-0.35, 0.57)	0.6409			0.00 (-0.47, 0.47)	0.9892		
IL-23**	0.02 (-0.09, 0.12)	0.7340			-0.14 (-0.40, 0.12)	0.2915			--0.10 (-0.36, 0.17)	0.4684		
IL-18/IL-1F4	-0.01 (-0.33, 0.32)	0.9758			-0.27 (-0.94, 0.39)	0.4203			-0.88 (-1.71, -0.04)	**0.0395**		
IL-28A/IFN-lambda2**	0.06 (-0.09, 0.21)	0.4207			-0.18 (-0.49, 0.12)	0.2417			0.08 (-0.20, 0.37)	0.5720		

All cytokines are expressed as Log10. Univariable general linear models were performed to study independent predictors of IgG Spike response.
Covariates showing a clinical and statistical significance or participating as a confounding factor for the variable of interest were included in the final step-wise multivariable models.
Abbreviations: 6-mercaptopurine (MP), Alkaline phosphatase (ALP), cerebrovascular disease (CVD), chronic obstructive pulmonary disease (COPD), chronic renal failure (CRF), diabetes mellitus (DM), estimate (E), hepatitis C virus (HCV), hepatitis B virus (HBV), hepatitis D virus (HDV), hepatocellular carcinoma (HCC), non-alcoholic fatty liver disease (NAFLD), standard error (SR).
* Includes autoimmune hepatitis, primary sclerosing cholangitis and primary biliary cholangitis.
** Cytokines with more than 25% undetectable values are not included in the multivariable models.

Supplementary Table 2. Proportion of vaccine types and univariable sensitivity analyses of IgG Spike response according to vaccine types.

Parameter	Cirrhosis (n=271)	Autoimmune liver disease (n=101)	Liver transplant (n=106)	Healthy controls (n=39)
Pfizer-BioNTech	213/260 (82%)<br>Ref.	66/99 (67%)<br>Ref.	48/99 (49%)<br>Ref.	20/38 (53%)<br>Ref.
Moderna	34/260 (13%)<br>0.38 (0.14, 0.62) (p=**0.0018**)	13/99 (13%)<br>0.67 (0.25, 1.09) (p=**0.0022**)	48/99 (49%)<br>0.03 (-0.38, 0.44) (p=0.8751)	6/38 (16%)<br>0.47 (-0.13, 1.06) (p=0.1206)
Janssen	6/260 (2%)<br>-0.48 (-1.05, 0.09) (p=0.0973)	5/99 (5%)<br>-0.93 (-1.58, -0.28) (p=**0.0053**)	1/99 (1%)<br>-0.65 (-2.75, 1.45) (p=0.5401)	---
Oxford-AstraZeneca	6/260 (2%)<br>-0.63 (-1.20, -0.06) (p=**0.0304**)	15/99 (15%)<br>-0.23 (-0.63, 0.17) (p=0.2534)	1/99 (1%)<br>1.12 (-0.38, 2.62) (p=0.1403)	8/38 (21%)<br>-0.43 (-0.96, 0.11) (p=0.1137)
Heterologous mRNA	1/260 (0.4%)<br>-0.69 (-2.07, 0.70) (p=0.3288)	---	---	---
Heterologous viral/mRNA	---	---	1/99 (1%)<br>0.30 (-1.80, 2.40) (p=0.7755)	4/38 (11%)<br>0.54 (-0.16, 1.24) (p=0.1274)

Univariable general linear models were used to study IgG Spike response according to vaccine types.

Supplementary Table 3. Characteristics of study population according to SARS-CoV-2 infection

Parameter	Cirrhosis			Autoimmune liver disease			Liver transplant		
	Non-infected (n=285)	Infected (n=20)	p-value	Non-infected (n=101)	Infected (n=12)	p-value	Non-infected (n=125)	Infected (n=10)	p-value
Sex: Male (n, %)	192 (67)	15 (75)	0.4799	24/101 (24)	2/12 (17)	0.7301	92/125 (74)	7/10 (70)	0.7261
Age (years; median, p25-p75)	61 (56-69)	57 (50- 68)	0.1122	(101) 59 (49-65)	(12) 48 (44-57)	**0.0459**	(125) 63 (56-69)	(10) 63 (58-72)	0.7883
Race (n, %)									
White	279/281 (99)	19/19 (100)	1	100/101 (99)	12/12 (100)	1	124/125 (99)	10/10 (100)	1
Black or Afro-American	2/281 (1)	0/19 (0)		1/101 (1)	0/0 (0)		1/125 (1)	0/0 (0)	
Ethnicity (n, %)									
North European	31/284 (11)	0/20 (0)		8/101 (8)	1/12 (8)		2/125 (2)	0/10 (0)	
Mediterranean	239/284 (84)	18/20 (90)	0.198	85/101 (84)	10/12 (83)	0.2863	120/125 (96)	10/10 (100)	1
Latin American	6/284 (2)	1/20 (5)		7/101 (7)	0/12 (0)		1/125 (1)	0/0 (0)	
Other	8/284 (3)	1/20 (5)		1/101 (1)	1/12 (0)		2/125 (2)	0/0 (0)	
Alcohol consumption (n, %)									
No	115/258 (45)	7/17 (41)	0.8889	94/100 (94)	12/12 (100)	1	89/125 (71)	5/10 (50)	0.1803
Former drinker	108/258 (42)	7/17 (41)		2/100 (2)	0/12 (0)		31/125 (25)	4/10 (40)	
Current drinker	35/258 (14)	3/17 (18)		4/100 (4)	0/12 (0)		5/125 (4)	1/10 (10)	
Tobacco consumption (n, %)									
No	146/258 (57)	9/17 (53)	0.787	73/100 (73)	7/12 (58)	0.1487	74/125 (59)	4/10 (40)	0.0761
Former smoker	57/258 (22)	5/17 (29)		12/100 (12)	4/12 (33)		32/125 (26)	6/10 (60)	
Current smoker	55/258 (21)	3/17 (18)		15/100 (15)	1/12 (8)		19/125 (15)	0/10 (0)	
Aetiology (n, %)									
Alcohol	144/285 (51)	11/20 (55)	0.6989	NA	NA		NA	NA	
Hepatitis B or C virus	111/285 (39)	9/20 (45)	0.5922						
NAFLD/NASH	56/285 (20)	6/20 (30)	0.2588						
Autoimmune*	39/285 (14)	0/20 (0)	0.0883						
Others	15/285 (5)	2/20 (10)	0.3082						
Years since diagnosis (n, %)									
<1	28/236 (12)	2/16 (13)	0.7445	6/67 (9)	0/4 (0)	0.1651	0/110 (0)	0/8 (0)	1
1-5	68/236 (29)	3/16 (19)		18/67 (27)	3/4 (75)		19/110 (17)	1/8 (13)	
>5	140/236 (59)	11/16 (69)		43/67 (64)	1/4 (25)		91/110 (83)	7/8 (88)	
History of acute decompensation (n,%)	129/240 (54)	11/16 (69)	0.2432	0/77 (0)	0/6 (0)		52/101 (52)	5/6 (83)	0.2115
HCC at time of sampling	45/238 (19)	2/16 (13)	0.7436	0/97 (0)	0/11 (0)		NA	NA	
Within Milan criteria	25/45 (56)	2/2 (100)	0.5005						

Without Milan criteria	20/45 (44)	0/2 (0)							
HCC on explant							36/122 (30)	2/10 (20)	
Within Milan criteria	NA	NA		NA	NA		32/36 (89)	2/2 (100)	0.7233
Without Milan criteria							4/36 (11)	0/2 (0)	
MELDNa ≥ 14 (n, %)	87/272 (32)	8/19 (42)	0.3631	NA	NA		NA	NA	
Child-Turcotte-Pugh class week 7 (n, %)									
A	188/285 (66)	11/20 (55)	0.1525	NA	NA		NA	NA	
B	83/285 (29)	6/20 (30)							
C	14/285 (5)	3/20 (15)							
Comorbidities (n, %)									
COPD	18/258 (7)	3/17 (18)	0.1299	1/100 (1)	0/12 (0)	1	5/125 (4)	0/10 (0)	1
Heart failure	5/258 (2)	0/17 (0)	1	0/100 (0)	0/12 (0)		1/125 (1)	0/10 (0)	1
Arterial hypertension	103/258 (40)	6/17 (35)	0.7055	19/100 (19)	3/12 (25)	0.7008	56/125 (45)	3/10 (30)	0.5125
Coronary artery disease	21/258 (8)	2/17 (12)	0.6419	2/100 (2)	1/12 (8)	0.2905	5/125 (4)	1/10 (10)	0.3756
Chronic renal failure	12/258 (5)	0/17 (0)	1	2/100 (2)	0/12 (0)	1	9/125 (7)	2/10 (20)	0.1893
Cerebrovascular disease	15/258 (6)	1/17 (6)	1	1/100 (1)	0/12 (0)	1	6/125 (5)	0/10 (0)	1
Diabetes mellitus	65/258 (25)	5/17 (29)	0.7742	3/100 (3)	0/12 (0)	1	40/125 (32)	1/10 (10)	0.2818
Psychiatric disorders	14/258 (5)	3/17 (18)	0.0777	4/100 (4)	0/12 (0)	1	4/125 (3)	0/10 (0)	1
Other chronic systemic disease	68/258 (26)	6/17 (35)	0.4082	42/100 (42)	2/12 (17)	0.1213	29/125 (23)	5/10 (50)	0.1212
Laboratory (median, p25-p75); mean, SD)									
Albumin (g/dL)	(285) 3.9 (3.5-4.3)	(20) 3.6 (3.0-4.0)	0.069	(101) 4.3 (4.1-4.4)	(12) 4.4 (4.1-4.6)	0.4098	(108) 4.2 (4.0-4.4)	(9) 4.2 (3.9-4.3)	0.9104
AST (U/L)	(262) 35 (25-55)	(19) 47 (29-60)	0.2259	(99) 27 (22-35.	(12) 26 (21-42)	0.9962	(121) 24 (18-31)	(10) 24 (18-27)	0.8219
ALT (U/L)	(267) 26 (17-40)	(19) 30 (22-41)	0.3634	(99) 25 (19-35)	(12) 23 (16-50)	1	(121) 21 (15-34)	(10) 27 (17-32)	0.659
Alkaline phosphatase (U/L)	(237) 112 (83-154)	(18) 98 (84-141)	0.426	(95) 88 (66-126)	(12) 93 (62-109)	0.7865	(118) 95 (65-124)	(9) 70 (58-94)	0.2778
GGT (U/L)	(253) 57 (29-116)	(19) 69 (37-112)	0.5378	(99) 28 (15-60)	(12) 28 (18-53)	1	(120) 32 (19-78)	(10) 32 (17-105)	0.8649
Total Bilirubin (mg/dL)	(285) 1.03 (0.7-1.8)	(20) 1.48 (0.9-2.4)	0.0844	(101) 0.60 (0.5-0.8)	(12) 0.59 (0.4-0.7)	0.7694	(117) 0.65 (0.5-0.9)	(9) 0.86 (0.6-1.1)	0.1749
Creatinine (mg/dL)	(280) 0.81 (0.7-1)	(20) 0.79 (0.7-1.0)	0.9362	(101) 0.79 (0.6-0.9)	(12) 0.74 (0.6-0.8)	0.2987	(120) 1.08 (0.9-1.3)	(9) 1.12 (0.9-1.3)	0.9779
Sodium (mEq/L)	(275) 139 (137-140)	(19) 138 (136-141)	0.7144	(98) 140 (139-142)	(12) 142 (139-142)	0.5966	(115) 141 (139-142)	(9) 141 (139-142)	0.8463
Total cholesterol (mg/dL)	(123) 153 (131-186)	(11) 143 (121-167)	0.2082	(76) 194 (171-226)	(12) 209 (168-227)	0.8037	(100) 168 (143-200)	(8) 171 (152-185)	0.6994
HDL-Cholesterol (mg/dL)	(76) 48.7 +/- 20.8	(3) 44.3 +/- 22.8	0.725	(55) 63.1 +/- 19.5	(9) 47.8 +/- 28.09	**0.0446**	(53) 45.0 +/- 15.20	(2) 45.0 +/- 24.04	0.9992
LDL-Cholesterol (mg/dL)	(66) 83 (56-103)	(3) 80 (68-92)	0.8604	(52) 104 (91-136)	(8) 111 (88- 133)	0.8624	(40) 102 (82-122)	(2) 90 (87-92)	0.5777
Triglycerides (mg/dL)	(122) 85 (65-103)	(10) 86 (77-101)	0.9795	(72) 88 (72-114)	(12) 99 (52-127)	0.8235	(97) 104 (78-169)	(8) 90 (68-133)	0.2464
C-reactive protein (mg/L)	(95) 5.2 (1.2-12.3)	(11) 15 (4.9-26.0)	**0.0144**	(7) 1.7 (1.1-2.1)			(24) 1.8 (0.7-6.5)	(2) 24.5 (1.0-48)	0.7385
Haemoglobin (g/dL)	(272) 12.7 (11-14)	(20) 13.0 (11-14)	0.3147	(98) 13.5 (13- 15)	(12) 13.1 (12-14)	0.176	(121) 14.2 (13-15)	(10) 12.5 (12-16)	0.3875
Leucocyte (x10^9cells/L)	(249) 5.1 (3.6-7.0)	(19) 4.6 (3.7- 5.2)	0.2753	(98) 6.0 (5.0-7.1)	(12) 5.8 (4.8-6.8)	0.4884	(120) 5.7 (4.5-7.1)	(10) 5.7 (5.3-6.1)	0.9028
Lymphocytes (x10^9cells/L)	(226) 1.2 (0.8-1.9)	(16) 1.0 (0.6-1.5)	0.1588	(93) 1.8 (1.4-2.4)	(12) 2.0 (1.4-2.5)	0.6804	(111) 1.5 (1.0-2.0)	(10) 1.9 (1.3-2.0)	0.2216

Monocytes (x10^9cells/L)	(189) 0.5 (0.4-0.7)	(13) 0.5 (0.4-0.6)	0.5968	(81) 0.5 (0.4-0.6)	(8) 0.4 (0.4-0.6)	0.3142	(51) 0.5 (0.4-0.6)	(3) 0.5 (0.4-0.5)	0.5358
Neutrophils (x10^9cells/L)	(226) 3.0 (2.0-4.1)	(16) 2.5 (1.9-3.5)	0.3185	(93) 3.2 (2.7-4.2)	(12) 3.1 (2.5-3.6)	0.4487	(111) 3.2 (2.5-4.5)	(10) 2.9 (2.4-3.7)	0.3158
Neutrophil/Lymphocyte ratio (%)	(121) 3.0 (2.0-11)	(8) 2.9 (1.9-43.0)	1	(24) 2.0 (1.3-2.7)	(6) 2.2 (2.0-3.0)	0.4572	(69) 2.4 (1.7-3.2)	(7) 1.7 (1.0-2.5)	0.1082
Platelet (x10^3cells/uL)	(177) 123 (76-181)	(11) 60 (43-167)	**0.0266**	(47) 252 (205-283)	(8) 261 (171-292)	0.7216	(97) 150 (113-214)	(7) 176 (127-197)	0.587
INR	(285) 1.2 (1.1-1.3)	(20) 1.2 (1.1-1.4)	0.6855	(101) 1.0 (0.9-1.0)	(12) 1.0 (0.9-1.0)	0.6218	(103) 1.0 (1.0-1.1)	(8) 1.0 (1.0-1.2)	0.6546
Prothrombin time (sec)	(102) 14 (12-16)	(11) 15 (13-19)	0.5174	(73) 11 (11-12)	(12) 12 (11-12)	0.7482	(80) 13 (13-14)	(7) 13 (13-16)	0.3312
Immunosuppressive drugs (n, %)									
Steroids	13/285 (5)	1/20 (5)	1	27/101 (27)	3/12 (25)	1	16/125 (13)	1/10 (10)	1
Calcineurin antagonist	3/285 (1)	1/20 (5)	0.2387	8/101 (8)	0/12 (0)	0.5967	110/125 (88)	8/10 (80)	0.614
Mycophenolate	3/285 (1)	1/20 (5)	0.2387	8/101 (8)	0/12 (0)	0.5967	63/125 (50)	7/10 (70)	0.3284
Azathioprine or 6-MP	3/285 (1)	0/20 (0)	1	27/101 (27)	5/12 (42)	0.3156	1/125 (1)	0/10 (0)	1
Other	0/285 (0)	0/20 (0)	---	0/101 (0)	1/12 (8)	0.1062	16/125 (13)	0/10 (0)	0.607
Vaccine brand, n (%)									
mRNA	230/285 (81)	19/20 (95)	0.1599	73/101 (72)	11/12 (92)	0.0923	120/125 (96)	9/10 (90)	0.3756
Viral	14/285 (5)	1/20 (5)		23/101 (23)	0/12 (0)		2/125 (2)	1/10 (10)	
Heterologous combination	41/285 (14)	0/20 (0)		5/101 (5)	1/12 (8)		3/125 (2)	0/10 (0)	
Cytokines									
IL-6	(269) -0.39 (-2.4-0.2)	(19) 0.13 (-2.4-0.6)	0.238	(90) -2.41 (-2.4--1.2)	(12) -2.41 (-2.4--2.4)	0.0869	(103) -1.28 (-2.4--0.2)	(10) -2.4 (-2.4--1.3)	0.4391
IL-33	(269) -2.66 (-2.7--2.7)	(19) -2.66 (-2.7--2.7)	0.7849	(90) -2.66 (-2.7--2.7)	(12) -2.66 (-2.7--2.7)	0.1344	(103) -2.66 (-2.7--2.7)	(10) -2.66 (-2.7--2.7)	0.2631
IL-8/CXCL8	(268) 0.86 (0.6-1.2)	(19) 0.87 (0.6-1.3)	0.5466	(90) 0.74 (0.6-1.0)	(12) 0.74 (0.6-1.4)	0.756	(103) 0.72 (0.5-1.0)	(10) 0.55 (-0.2-1.0)	0.456
CXCL10/IP-10/CRG-2	(269) 1.00 (0.8-1.2)	(19) 1.02 (0.9-1.2)	0.6579	(90) 0.8 +/- 0.25	(12) 0.8 +/- 0.31	0.6257	(104) 0.9 +/- 0.28	(10) 1.0 +/- 0.26	0.4175
IL-10	(269) -2.16 (-2.2--0.6)	(19) -2.16 (-2.2--2.2)	0.0193	(90) -2.16 (-2.2--0.6)	(12) -2.16 (-2.2--2.2)	0.1005	(103) -2.16 (-2.2--0.7)	(10) -2.16 (-2.2--1.3)	0.5282
IL-27	(269) 1.13 (-0.3-2.0)	(19) 0.50 (-0.3-1.7)	0.6565	(90) -0.25 (-0.3--0.3)	(12) -0.25 (-0.3-1.6)	0.4138	(103) 0.50 (-0.3-1.8)	(10) 0.84 (-0.3-1.8)	0.9487
IL-2	(269) -1.80 (-1.8-0.1)	(19) -0.40 (-1.8-0.5)	0.0585	(90) -1.80 (-1.8--0.5)	(12) -1.18 (-1.8-0.2)	0.2168	(103) -1.80 (-1.8-0.1)	(10) -1.19 (-1.8-0.6)	0.3488
IFN-gamma	(269) -3.12 (-3.1--1.6)	(19) -3.12 (-3.1-0.2)	0.8548	(90) -3.12 (-3.1--1.6)	(12) -3.12 (-3.1--1.5)	0.9481	(103) -3.12 (-3.1--1.6)	(10) -3.12 (-3.1--1.6)	0.3549
IL-1ra/IL-1F3	(269) 2.31 (2.2-2.5)	(19) 2.24 (2.1-2.6)	0.9841	(90) 2.4 +/- 0.20	(12) 2.3 +/- 0.20	0.2272	(103) 2.4 +/- 0.25	(10) 2.3 +/- 0.37	0.2043
CCL3/MIP-1 alpha	(269) 0.43 (0.4-0.4)	(19) 0.43 (0.4-0.4)	0.4222	(90) 0.43 (0.4-0.4)	(12) 0.43 (0.4-0.4)	0.368	(103) 0.43 (0.4-0.4)	(10) 0.43 (0.4-0.4)	0.4884
CCL4/MIP-1 beta	(269) 1.52 (1.0-1.7)	(19) 1.37 (0.7-1.6)	0.0614	(90) 1.63 (1.4-1.8)	(12) 1.60 (1.0-1.8)	0.8599	(103) 1.51 (0.7-1.7)	(10) 1.48 (0.7-1.6)	0.4253
IL-1 alpha/IL-1F1	(269) -1.51 (-1.5--1.5)	(19) -1.51 (-1.5--1.5)	0.5542	(90) -1.51 (-1.5--1.5)	(12) -1.51 (-1.5--1.5)	0.7385	(103) -1.51 (-1.5--1.5)	(10) -1.51 (-1.5--1.5)	0.5393
IL-4	(269) -0.82 (-0.8--0.8)	(19) -0.82 (-0.8--0.8)	0.8633	(90) -0.82 (-0.8--0.8)	(12) -0.82 (-0.8--0.8)	0.2626	(103) -0.82 (-0.8--0.8)	(10) -0.82 (-0.8--0.8)	0.6704
IL-17/IL-17A	(269) -2.24 (-2.2--2.2)	(19) -2.24 (-2.2--2.2)	0.6998	(90) -2.24 (-2.2--2.2)	(12) -2.24 (-2.2--2.2)	0.1501	(103) -2.24 (-2.2--2.2)	(10) -2.24 (-2.2--2.2)	0.7895
APRIL/TNFSF13	(269) 2.56 (2.4-2.7)	(19) 2.49 (2.4-2.8)	0.7563	(90) 2.7 +/- 0.13	(12) 2.7 +/- 0.20	0.7338	(103) 2.60 (2.4-2.8)	(10) 2.51 (2.4-2.6)	0.1799
BAFF/BLyS/TNFSF13B	(269) 2.6 +/- 0.21	(19) 2.6 +/- 0.18	0.1873	(90) 2.39 (2.3-2.5)	(12) 2.40 (2.4-2.5)	0.5586	(103) 2.4 +/- 0.15	(10) 2.4 +/- 0.13	0.7629
Lymphotoxin-alpha/TNF-beta	(269) -2.05 (-2.1--2.1)	(19) -2.05 (-2.1--2.1)	0.9928	(90) -2.05 (-2.1--2.1)	(12) -2.05 (-2.1--2.1)	0.536	(103) -2.05 (-2.1--2.1)	(10) -2.05 (-2.1--2.1)	0.34

IL-13	(255) 0.49 (0.5-0.8)	(17) 0.49 (0.5-0.5)	0.149	(83) 0.49 (0.5-1.5)	(12) 0.49 (0.4-1.5)	0.5462	(80) 0.49 (0.5-1.5)	(9) 1.48 (0.5-1.8)	0.183
IL-5	(269) -1.18 (-1.2--1.2)	(19) -1.18 (-1.2--1.2)	0.2941	(90) -1.18 (-1.2--1.2)	(12) -1.18 (-1.2--1.2)	0.62	(103) -1.18 (-1.2--1.2)	(10) -1.18 (-1.2--1.2)	0.7797
IL-12p70	(269) -1.65 (-1.7--0.1)	(19) -1.65 (-1.7--0.0)	0.7298	(90) -1.65 (-1.7--1.7)	(12) -1.65 (-1.7-0.5)	0.1711	(103) -1.65 (-1.7-0.1)	(10) -1.65 (-1.7-0.5)	0.9515
CCL2/JE/MCP-1	(269) 2.02 (1.9-2.2)	(19) 2.02 (1.8-2.2)	0.9353	(90) 2.1 +/- 0.22	(12) 2.1 +/- 0.21	0.4095	(103) 2.12 (2-2.2)	(10) 1.98 (1.9-2.2)	0.2257
IL-15	(269) -2.87 (-2.9--0.9)	(19) -1.40 (-2.9--0.6)	0.2617	(90) -2.87 (-2.9--0.8)	(12) -1.87 (-2.9--0.2)	0.4939	(103) -2.87 (-2.9--0.5)	(10) -0.81 (-2.9--0.3)	0.5893
TNF-alpha	(269) 0.20 (-0.1-0.4)	(19) 0.17 (-0.2-0.3)	0.9229	(90) -0.04 (-0.4-0.2)	(12) -0.31 (-1.4-0.2)	0.2187	(103) 0.27 (0.1-0.4)	(10) 0.35 (0.0-0.4)	0.8837
IL-28B/IFN-lambda3	(269) 0.41 (0.4-0.4)	(19) 0.41 (0.4-0.4)	0.2134	(90) 0.41 (0.4-0.4)	(12) 0.41 (0.4-0.4)	0.4147	(103) 0.41 (0.4-0.8)	(10) 0.41 (0.41-0.41)	0.0653
CD40Ligand/TNFSF5	(269) 3.09 (2.8-3.3)	(19) 3.00 (2.8-3.3)	0.6233	(90) 3.37 (3.2-3.5)	(12) 3.29 (2.9-3.5)	0.2331	(103) 2.99 (2.7-3.2)	(10) 2.55 (2.2-3.1)	0.0875
IL-23	(267) -0.40 (-0.4-1.1)	(18) -0.40 (-0.4-1.1)	0.7645	(89) -0.40 (-0.4--0.4)	(12) -0.40 (-0.4--0.4)	0.2335	(97) -0.40 (-0.4-1.1)	(10) -0.40 (-0.4--0.4)	0.0902
IL-18/IL-1F4	(269) 1.9 +/- 0.25	(19) 1.9 +/- 0.30	0.248	(90) 1.7 +/- 0.23	(12) 1.8 +/- 0.33	0.7375	(103) 1.9 +/- 0.22	(10) 1.9 +/- 0.28	0.8242
IL-28A/IFN-lambda2	(269) 0.04 (0.0-0.9)	(19) 0.04 (0.0-1.3)	0.5101	(90) 0.04 (0.0-0.5)	(12) 0.04 (0.0-0.5)	0.9097	(103) 0.04 (0.0-1.3)	(10) 1.00 (0.0-1.6)	0.327
Anti-SARS-CoV-2 antibodies									
IgG SARS-CoV-2 Spike	(264) 4.60 (4.2-5.1)	(18) 4.86 (4.3-5.5)	0.2189	(88) 4.63 (4.2-5.0)	(12) 4.70 (4.6-5.1)	0.2749	(93) 4.09 (3.3-4.7)	(10) 3.83 (2.8-4.8)	0.6934
IgG/IgM ratio SARS-CoV-2 Spike	(263) 2.05 (1.6-2.4)	(18) 2.12 (1.7-2.4)	0.8188	(88) 1.90 (1.4-2.3)	(12) 1.76 (1.1-1.9)	0.116	(93) 1.53 (0.9-2.1)	(10) 1.30 (0.6-1.7)	0.2656
Neutr. Spike Reference Strain	(269) 2.43 (0.7-5.2)	(19) 4.09 (2.6-6.8)	0.043	(90) 2.6 +/- 2.66	(12) 3.4 +/- 2.14	0.3219	(102) 0.19 (-1.3-3.4)	(10) -0.61 (-1.1-2.6)	0.9959
Neutr. S1 RBD Reference Strain	(269) 3.11 (1.5-5.3)	(19) 4.69 (3.3-6.5)	0.0378	(90) 3.16 (1.3-4.9)	(12) 4.14 (2.7-5.1)	0.2496	(103) 0.93 (0.2-3.8)	(10) 0.57 (0.4-3.3)	0.9077
Neutr. Alpha Spike	(269) 1.65 (0.2-4.1)	(19) 3.24 (1.7-6.4)	0.0397	(90) 1.68 (0.0-3.1)	(12) 2.52 (1.3-3.9)	0.3136	(102) -0.26 (-1.3-2.2)	(10) -0.88 (-1.1-1.6)	0.8987
Neutr. Beta Spike	(268) 0.65 (-0.6-2.5)	(19) 2.27 (0.4-5.5)	0.0148	(89) 0.56 (-0.8-1.8)	(12) 0.85 (0.1-2.5)	0.4361	(101) -0.99 (-1.8-0.6)	(10) -1.29 (-1.4-0.4)	0.9959
Neutr. Gamma Spike	(267) 0.76 (-0.7-2.8)	(19) 2.48 (0.5-5.7)	0.022	(89) 0.61 (-0.8-2.3)	(12) 0.85 (0.1-2.9)	0.4239	(102) -1.12 (-2.0-0.6)	(10) -1.43 (-1.8-0.3)	0.9716
Neutr. Delta Spike	(269) 1.27 (-0.1-3.9)	(19) 3.13 (1.0-6.4)	0.0383	(90) 1.6 +/- 2.66	(12) 2.4 +/- 2.07	0.3243	(99) -0.51 (-1.8-2.3)	(10) -0.95 (-1.6-1.4)	0.9708
Neutr. Omicron Spike	(217) -0.93 (-2.4-0.5)	(15) 0.77 (0.4-2.0)	0.0017	(62) -1.27 (-2.1-0.2)	(11) -0.80 (-2.2--0.1)	0.9694	(70) -1.88 (-2.4--0.5)	(7) -1.70 (-3.0-0.8)	0.9226

All cytokines are expressed as Log10.

Abbreviations: Chronic obstructive pulmonary disease (COPD), hepatocellular carcinoma (HCC), Interquartile range (IQR), not applicable (NA), standard deviation (S
6-mercaptopurine (MP).

* Includes autoimmune hepatitis, primary sclerosing cholangitis and primary biliary cholangitis.

Supplementary Table 4. Univariable and multivariable analyses of time to breakthrough infection (complete analysis)

	Cirrhosis				Autoimmune liver disease				Liver transplant			
	Univariable		Multivariable		Univariable		Multivariable		Univariable		Multivariable	
**Parameter**	**HR (95CI)**	**p-value**	**HR (95CI)**	**p-value**	**HR (95CI)**	**p-value**	**HR (95CI)**	**p-value**	**HR (95CI)**	**p-value**	**HR (95CI)**	**p-value**
**Sex (female)**	0.61 (0.2-1.7)	0.3404			0.75 (0.2-3.5)	0.7128			0.98 (0.2-4.7)	0.9756		
**Age**	0.97 (0.9-1.0)	0.1679			0.95 (0.9-0.99)	**0.0468**	0.92 (0.8-1.0)	**0.0216**	1.07 (0.9-1.2)	0.1125		
**Race**												
White	Ref.				Ref.				Ref.			
Black / Afro-American	---	---			---	---			---	---		
**Ethnicity**												
Mediterranean	0.46 (0.1-2.0)	0.3045			0.42 (0.1-1.9)	0.2713			---	---		
Other	Ref.				Ref.							
**Alcohol consumption**												
No	Ref.				Ref.				Ref.			
Former drinker	1.20 (0.4-3.6)	0.7457			---	---			6.27 (1.5-25.7)	**0.0108**		
Current drinker	1.57 (0.4-6.1)	0.9175			---	---			6.11 (0.7-54.8)	0.1057		
**Tobacco consumption**												
No	Ref.				Ref.				Ref.			
Former smoker	1.47 (0.5-4.4)	0.4902			3.04 (0.8-11.9)	0.7788			1.99 (0.5-7.5)	0.3021		
Current smoker	0.92 (0.2-4.3)	0.9175			0.74 (0.1-6.1)	0.109			---	---		
**Aetiology**												
Alcohol	1.45 (0.6-3.6)	0.4172										
Viral	1.25 (0.5-3.1)	0.6259			NA				NA			
NAFLD	2.90 (1.1-7.7)	**0.0316**										
Autoimmune*	---	---										
Other	1.05 (0.2-4.6)	0.9511										
**Years since diagnosis**												
<1	0.96 (.2-4.4)	0.9616			---	---			---	---		
1-5	0.56 (0.2-2.0)	0.3754			10.8 (1.1-105)	**0.0408**			0.73 (0.1-6.1)	0.7713		
>5	Ref.				Ref.				Ref.			
**History of acute decompensation**	2.27 (0.8-6.7)	0.1358			---	---			7.19 (0.8-64.6)	0.0785		
**HCC at time of sampling**	0.81 (0.2-3.6)	0.7865			---	---			NA			
**MELDNa week 7**	1.05 (1.0-1.1)	0.2401			NA				NA			
Child-Turcotte-Pugh class					NA				NA			
A	0.61 (0.2-1.5)	0.2993										

B+C	Ref.											
**Comorbidities**												
COPD	4.73 (1.3-16.7)	**0.0159**			---	---			---	---		
Heart failure	---	---			---	---			---	---		
Hypertension	1.06 (0.4-2.9)	0.9136			1.71 (0.4-8.3)	0.5031			0.40 (0.1-1.6)	0.1976		
Coronary disease	2.85 (0.6-13.4)	0.1861			7.39 (0.9-60.2)	0.0617			3.24 (0.4-26.1)	0.269		
CRF	---	---			---	---			3.41 (0.7-16.5)	0.1262		
CVD	0.84 (0.1-6.4)	0.8668			---	---			---	---		
DM	1.10 (0.4-3.4)	0.8727			---	---			0.22 (0.0-1.8)	0.1543		
Psychiatric	2.73 (0.8-9.7)	0.1197			---	---			---	---		
Other disease	1.23 (0.4-3.4)	0.6896			0.60 (0.1-2.8)	0.5066			3.17 (0.9-11.8)	0.0857		
**Laboratory**												
Albumin	0.48 (0.3-0.9)	**0.0168**	0.47 (0.2-1.0)	**0.0437**	0.87 (0.1-6.1)	0.8896			0.79 (0.2-3.8)	0.7666		
AST	1.01 (0.9-1.02)	0.4105			1.01 (0.9-1.0)	0.4967			0.98 (0.9-1.0)	0.5477		
ALT	1.01 (0.9-1.02)	0.3825			1.00 (0.9-1.0)	0.6848			0.99 (0.9-1.0)	0.751		
ALP	1.00 (0.9-1.01)	0.985			0.99 (0.9-1.0)	0.4089			1.00 (0.9-1.0)	0.5707		
GGT	1.00 (0.9-1.01)	0.699			0.99 (0.9-1.0)	0.3072			1.00 (0.9-1.0)	0.3539		
Total Bilirubin	1.03 (0.9-1.2)	0.6844			0.59 (0.1-5.1)	0.6355			1.69 (0.8-3.4)	0.1421		
Creatinine	1.48 (0.2-9.6)	0.6846			0.16 (0.0-8.9)	0.3692			0.61 (0.1-5.7)	0.6687		
Sodium	0.93 (0.8-1.1)	0.2898			0.92 (0.7-1.2)	0.5049			0.98 (0.7-1.3)	0.9018		
Total cholesterol	0.99 (0.9-1.0)	0.1108			1.00 (0.9-1.0)	0.8985			1.00 (0.9-1.0)	0.7945		
HDL-Cholesterol	0.98 (0.9-1.0)	0.3887			0.96 (0.9-0.99)	**0.0041**	0.94 (0.9-1.0)	**0.0031**	1.04 (0.9-1.2)	0.5556		
LDL-Cholesterol	0.99 (0.9-1.0)	0.6263			0.99 (0.9-1.0)	0.6967			1.01 (0.9-1.1)	0.9308		
Triglycerides	0.99 (0.9-1.0)	0.7565			0.99 (0.9-1.0)	0.44			0.99 (0.9-1.0)	0.3804		
C-reactive protein	1.00 (0.9-1.0)	0.2038			---	---			1.02 (0.9-1.1)	0.3752		
Haemoglobin	1.08 (0.9-1.3)	0.3414			0.68 (0.5-0.9)	**0.004**			0.83 (0.6-1.1)	0.2526		
Leucocyte	0.91 (0.7-1.2)	0.4353			0.99 (0.8-1.3)	0.9681			0.95 (0.7-1.2)	0.6729		
Lymphocytes	0.66 (0.3-1.4)	0.2623			1.06 (0.5-2.2)	0.8829			0.97 (0.6-1.6)	0.9126		
Monocytes	1.27 (0.4-3.8)	0.6772			0.24 (0.0-24.1)	0.5397			0.05 (0.0-69.1)	0.4125		
Neutrophils	0.88 (0.6-1.3)	0.5055			1.11 (0.7-1.7)	0.6319			0.93 (0.7-1.3)	0.7071		
Neutrophil/Lymphocyte	1.01 (0.9-1.0)	0.7052			2.29 (0.9-6.1)	0.0955			1.08 (0.5-2.5)	0.848		
Platelet	0.99 (1.0-1.0)	0.0539			1.00 (0.9-1.0)	0.7698			1.00 (0.9-1.0)	0.9078		
INR	1.47 (0.4-6.1)	0.5942			0.30 (0.0-48.6)	0.6409			0.62 (0.1-3.7)	0.5965		
Prothrombin time	---	---			1.27 (0.5-3.1)	0.5914			---	---		
**Immunosuppressive drugs**												
Steroids	1.58 (0.2-12.0)	0.6605			0.69 (0.2-3.2)	0.6471			0.91 (0.1-7.3)	0.9273		
Calcineurin antagonist	3.17 (0.4-24.0)	0.2647			---	---			0.37 (0.1-1.8)	0.2173		

Mycophenolate	5.25 (0.7-40.3)	0.1111			---	---			0.84 (0.2-3.4)	0.8005		
Azathioprine or 6-MP	---	---			1.90 (0.6-6.5)	0.305			---	---		
Other	---	---			6.25 (0.8-50.0)	0.0845			---	---		
**Vaccine brand**												
mRNA	Ref.				Ref.				Ref.			
Viral	0.90 (0.1-6.8)	0.9182			---				---	---		
Heterologous	---				---				---	---		
**Cytokines**												
IL-6	1.23 (0.8-1.8)	0.3027			0.47 (0.1-1.5)	0.2156			0.86 (0.4-1.7)	0.6562		
IL-33**	1.10 (0.8-1.5)	0.5506			---	---			---	---		
IL-8/CXCL8	2.10 (0.8-5.6)	0.1345			0.89 (0.4-2.1)	0.7935			0.72 (0.2-2.2)	0.5706		
CXCL10/IP-10/CRG-2	3.02 (0.8-11.8)	0.111			1.93 (0.2-21.1)	0.5895			1.66 (0.3-10.2)	0.586		
IL-10**	0.35 (0.1-0.9)	**0.0267**			0.52 (0.2-1.5)	0.2258			0.66 (0.2-1.8)	0.4039		
IL-27	0.93 (0.6-1.4)	0.7193			1.46 (0.8-2.6)	0.2102			0.87 (0.5-1.7)	0.672		
IL-2**	1.65 (1.0-2.7)	**0.0432**			1.29 (0.7-2.3)	0.3688			1.21 (0.6-2.3)	0.5645		
IFN-gamma	1.04 (0.8-1.3)	0.7627			0.92 (0.6-1.3)	0.64			0.77 (0.4-1.4)	0.3862		
IL-1ra/IL-1F3	1.32 (0.2-7.0)	0.7439			0.13 (0.0-2.2)	0.1585			0.08 (0.0-1.1)	0.0617		
CCL3/MIP-1 alpha**	---	---			---	---			---	---		
CCL4/MIP-1 beta	0.40 (0.1-1.1)	0.0883			0.89 (0.2-3.5)	0.8642			0.53 (0.1-2.1)	0.3609		
IL-1 alpha/IL-1F1**	---	---			---	---			---	---		
IL-4**	1.06 (0.5-2.2)	0.874			0.56 (0.1-2.4)	0.4389			0.77 (0.2-3.4)	0.7334		
IL-17/IL-17A	0.939 (0.5-1.6)	0.8249			---	---			1.02 (0.5-2.2)	0.9527		
APRIL/TNFSF13	0.76 (0.1-5.6)	0.786			0.74 (0.0-62.9)	0.8954			0.14 (0.0-1.5)	0.1016		
BAFF/BLyS/TNFSF13B	4.89 (0.9-27.4)	0.0709			0.32 (0.0-34-2)	0.6301			0.75 (0.0-57.4)	0.8959		
Lymphotoxin-alpha/TNF-beta	0.89 (0.5-1.7)	0.7198			---	---			---	---		
IL-13**	0.31 (0.1-1.6)	0.1623			0.90 (0.3-2.7)	0.8477			1.34 (0.4-4.1)	0.6073		
IL-5**	---	---			---	---			---	---		
IL-12p70	1.06 (0.7-1.6)	0.7792			1.51 (0.9-2.6)	0.1248			1.06 (0.5-2.1)	0.8591		
CCL2/JE/MCP-1	1.29 (0.2-8.0)	0.782			0.40 (0.0-6.0)	0.5104			0.81 (0.0-19.9)	0.8958		
IL-15	1.23 (0.8-1.8)	0.3032			1.39 (0.9-2.2)	0.1789			1.17 (0.7-2.0)	0.54		
TNF-alpha	1.37 (0.6-3.2)	0.4796			0.60 (0.4-1.3)	0.2665			0.87 (0.2-3.8)	0.8569		
IL-28B/IFN-lambda3	0.34 (0.1-1.9)	0.2178			---	---			---	---		
CD40Ligand/TNFSF5	0.83 (0.3-1.6)	0.753			0.78 (0.2-3.2)	0.7333			0.15 (0.0-0.7)	**0.0123**	0.15 (0.0-0.7)	**0.0123**
IL-23**	1.02 (0.6-1.9)	0.9406			0.54 (0.1-3.1)	0.488			---	---		
IL-18/IL-1F4	3.90 (0.7-22.7)	0.1303			2.78 (0.3-28.2)	0.3884			0.98 (0.0-27.9)	0.9891		
IL-28A/IFN-lambda2**	1.47 (0.7-3.3)	0.3466			2.88 (1.0-8.7)	0.0609			1.63 (0.6-4.1)	0.2985		
**Anti-SARS-CoV-2 antibodies**												
IgG SARS-CoV-2 Spike	1.22 (0.7-2.3)	0.537			1.63 (0.5-5.7)	0.4442			0.55 (0.3-1.2)	0.1229		
IgG/IgM ratio SARS-CoV-2 Spike	0.69 (0.3-1.4)	0.2969			0.49 (0.2-1.3)	0.1636			0.58 (0.2-1.6)	0.2812		

Neutr. Spike Reference Strain	1.13 (0.9-1.3)	0.1718			1.16 (0.9-1.5)	0.2786			0.97 (0.8-1.2)	0.8008		
Neutr. S1 RBD Reference Strain	1.17 (0.9-1.4)	0.153			1.25 (0.9-1.8)	0.2233			0.96 (0.7-1.4)	0.8364		
Neutr. Alpha Spike	1.14 (0.9-1.3)	0.1291			1.09 (0.8-1.4)	0.4808			0.94 (0.7-1.3)	0.676		
Neutr. Beta Spike	1.17 (1.0-1.4)	0.0503			1.06 (0.8-1.4)	0.6808			0.92 (0.6-1.3)	0.6367		
Neutr. Gamma Spike	1.12 (0.9-1.3)	0.1344			1.06 (0.8-1.4)	0.6501			0.88 (0.6-1.3)	0.5003		
Neutr. Delta Spike	1.12 (0.9-1.3)	0.1668			1.15 (0.9-1.5)	0.2766			0.95 (0.7-1.2)	0.7291		
Neutr. Omicron Spike	1.25 (1.1-1.5)	**0.0082**	1.18 (1.0-1.4)	0.0559	0.95 (0.7-1.3)	0.7408			0.98 (0.7-1.4)	0.9288		

All cytokines, IgG and IgM are expressed as Log10. Neutralization is expressed as Logit %. Proportional hazard models were used to study independent predictors of breakthrough infection. Independent covariates were included in the models when showing statistical significance or confounding. Porportional hazard assumptions were explored by testing zph based on the weighted Schoenfeld and PH assumptions were met for all variables included in the models.

Abbreviations: 6-mercaptopurine (MP), Alkaline phosphatase (ALP), cerebrovascular disease (CVD), chronic obstructive pulmonary disease (COPD), chronic renal failure (CRF), diabetes mellitus (DM), estimate (E), hepatitis C virus (HCV), hepatitis B virus (HBV), hepatitis D virus (HDV), hepatocellular carcinoma (HCC), non-alcoholic fatty liver disease (NAFLD), neutralization (neutr), standard error (SR).
* Includes autoimmune hepatitis, primary sclerosing cholangitis and primary biliary cholangitis.
** Cytokines with more than 25% undetectable values. These cytokines are not included in the multivariable models.

Supplementary Figure Legends:

Supplementary Fig 1. Serological measurements of post-vaccination anti-SARS-CoV-2 antibodies against Nucleocapsid. The first and second graph represent serum concentrations of anti-Nucleocapsid IgG antibodies in healthy controls, patients with cirrhosis (CIRRH), autoimmune liver disease (AILD) and liver transplant (LT).

All the measurements were obtained using electrochemoluminescence-based Meso Scale Discovery multiplex assays. All the serum samples were diluted 1:5000 for quantification, as recommended by the manufacturer. All the data are represented on Log10 scale scatterplot graphs; lines and error bars represent the geometric mean and 95% C.I. for each dataset. All the comparisons were assessed by one-way ANOVA followed by Dunn's corrected pairwise comparisons between observed group means. Significance set at alpha=0.05 (5.0e-002).

Supplementary Fig 2. Serological measurements of post-vaccination cross-reactive anti-Spike antibodies targeting SARS-CoV-1 and MERS-CoV. The top graph represents serum concentrations of IgG targeting SARS-CoV-1 Spike in healthy controls, patients with cirrhosis (CIRRH), autoimmune liver disease (AILD) and liver transplant (LT). The bottom graph represents serum concentrations of IgG targeting MERS-CoV Spike in the four groups of subjects.

All the measurements were obtained using electrochemoluminescence-based Meso Scale Discovery multiplex assays. All the serum samples were diluted 1:5000 for quantification, as recommended by the manufacturer. All the data are represented on Log10 scale scatterplot graphs; lines and error bars represent the geometric mean and 95% C.I. for each dataset. All the comparisons were assessed by one-way ANOVA followed by Dunn's corrected pairwise comparisons between observed group means. Significance set at alpha=0.05 (5.0e-002).

Supplementary Fig 3. Serological measurements of post-vaccination cross-reactive anti-Spike antibodies targeting common cold human coronaviruses (HCoVs). The graphs represent serum concentrations of IgG antibodies targeting Spike from HCoV 229E, HKU1, NL63 and OC43 respectively, in healthy controls, patients with cirrhosis (CIRRH), autoimmune liver disease (AILD) and liver transplant (LT).

All the measurements were obtained using electrochemoluminescence-based Meso Scale Discovery multiplex assays. All the serum samples were diluted 1:5000 for quantification, as recommended by the manufacturer. All the data are represented on Log10 scale scatterplot graphs; lines and error bars represent the geometric mean and 95% C.I. for each dataset. All the comparisons were assessed by one-way ANOVA followed by Dunn's corrected pairwise comparisons between observed group means. Significance set at alpha=0.05 (5.0e-002).

Supplementary Fig 4. Serum cytokine concentrations. The three boxplot panels represent serum concentrations of 29 cytokines in healthy controls, patients with cirrhosis (CIRRH), autoimmune liver disease (AILD) and liver transplant (LT).

All the measurements were obtained by Luminex multiplex assays using serum samples diluted 1:2, as recommended by the manufacturer. The data are represented on linear scale as box-

and-whiskers plots. For the purpose of graphic comparability of the relative cytokine levels by subject groups, the Log10-transformed serum concentration data for each cytokine were standardised to z-scores. For each boxplot, the box represents the 25th, 50th and 75th percentile (i.e. median and interquartile range, IQR); the whiskers include all the data up and including to the top or bottom values within 1.5x IQR from the respective box edge. Circles are the close outliers, between 1.5x and 3x IQR from the respective box edge; stars are the far outliers, beyond 3x IQR from the respective box edge.

Supplementary Fig 5. Comparative analysis of live SARS-CoV-2 neutralization. Microneutralization assay was conducted according to the method described by Algaissi and Hashem (REF). The virus used for this assay was the clinical isolate SARS-CoV-2/human/NLD/Leiden-0008/2020 (GenBank accession number: MT705206.1). Data are displayed as the neutralization titre in healthy controls, patients with cirrhosis (CIRRH), autoimmune liver disease (AILD) and liver transplant (LT). This value is calculated by dividing the number of positive wells with complete inhibition of the virus-induced cytopathogenic effect, by the number of replicates, and adding 2.5 to stabilize the calculated ratio. The neutralizing antibody titre was defined as the log2 reciprocal of this value. All neutralization titres above 5 were considered as positive. Lines and error bars represent geometric mean and 95% C.I. All comparisons were assessed by ANCOVA, adjusted for age, sex and time of sample collection post-vaccination, followed by Bonferroni-corrected pairwise comparisons between covariate-adjusted estimated group means.

Supplementary Fig 6. Comparative analysis of percentage neutralisation achieved (PNA) against Spike from the six viral variants tested by surrogate viral neutralisation assay (sVNT). Panels (A) to (D), PNA against SARS-CoV-2 Spike from Reference, Alpha, Beta, Gamma, Delta and Omicron variants was compared within each subject group: (A) healthy controls ~~(HC)~~, (B) cirrhosis (CIRRH), (C) autoimmune liver disease (AILD), and (D) liver transplant (LT). Panels (E) to (H), sensitivity analysis in the four subject groups after removing all subjects with Omicron PNA below detection limit.

All the measurements were obtained using electrochemoluminescence-based Meso Scale Discovery multiplex competitive binding assays. All the serum samples were diluted 1:12.5 for quantification, as recommended by the manufacturer. All the data are represented on Probability/Percentage Logit scale scatterplot graphs; lines and error bars represent the geometric mean and 95% C.I. for each dataset. The comparisons in panels (A) to (D) were assessed by Repeated Measures ANOVA with Greenhouse-Geisser sphericity correction (matching by subject). Due to the removal of subjects with Omicron PNA below detection limit from the Omicron data subset, the comparisons in panels (E) to (H) were assessed by Mixed Model ANOVA (matching by subject) instead. Both sets of omnibus tests were followed by Dunn's-corrected pairwise comparisons. Significance set at alpha=0.05 (5.0e-002). Significance asterisks used for pairwise comparisons: ****, p<0.0001.

Supplementary Figure 1

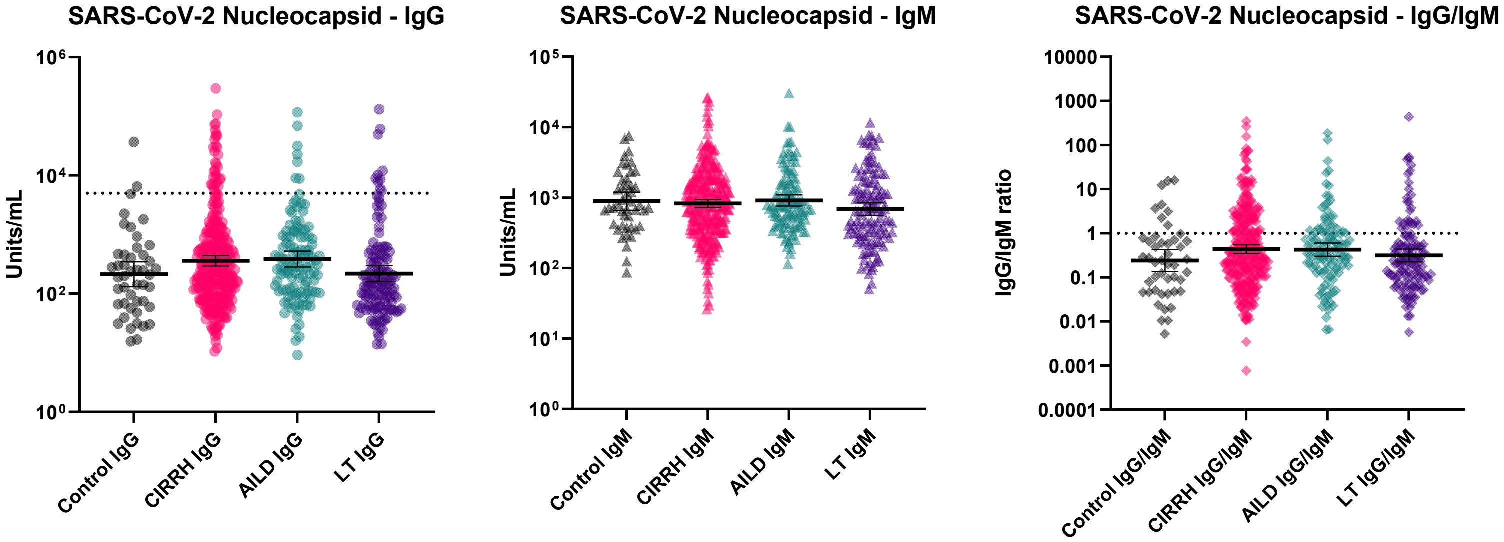

Supplementary Figure 2

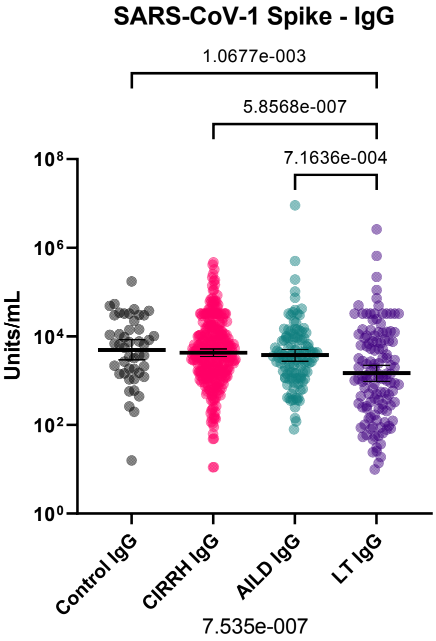

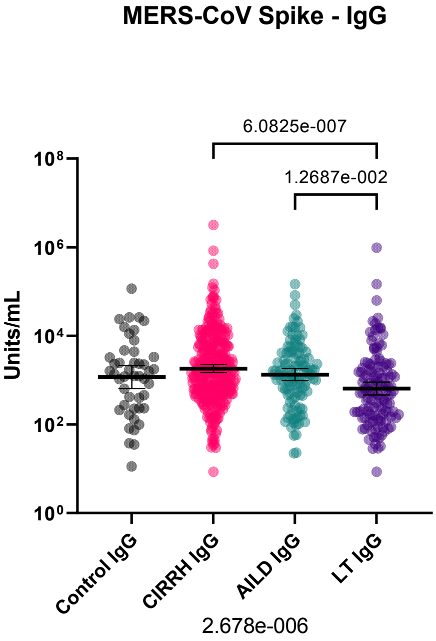

Supplementary Figure 3

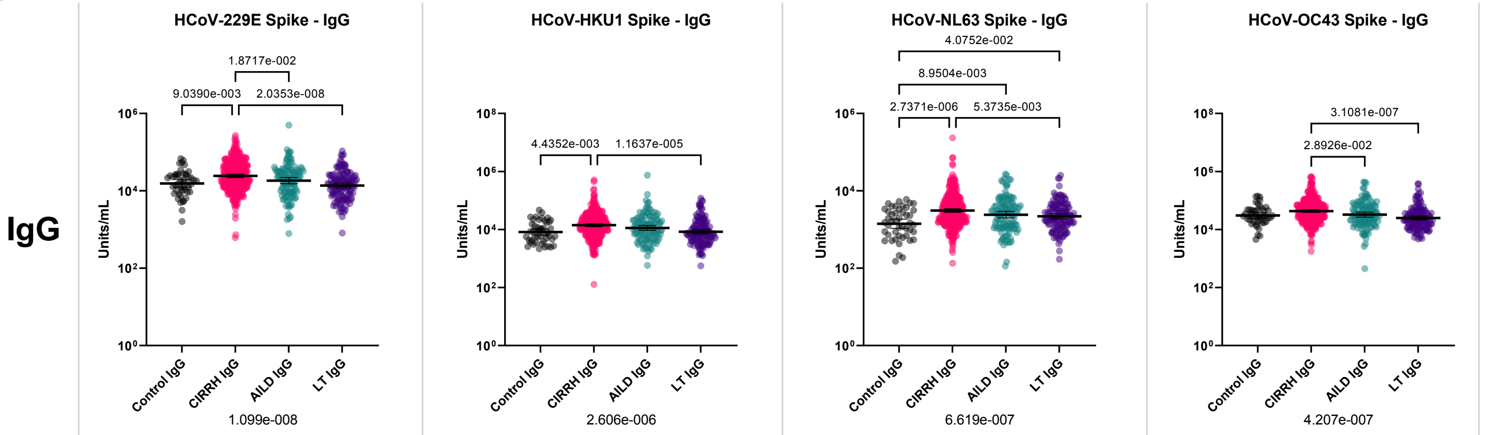

Supplementary Figure 4

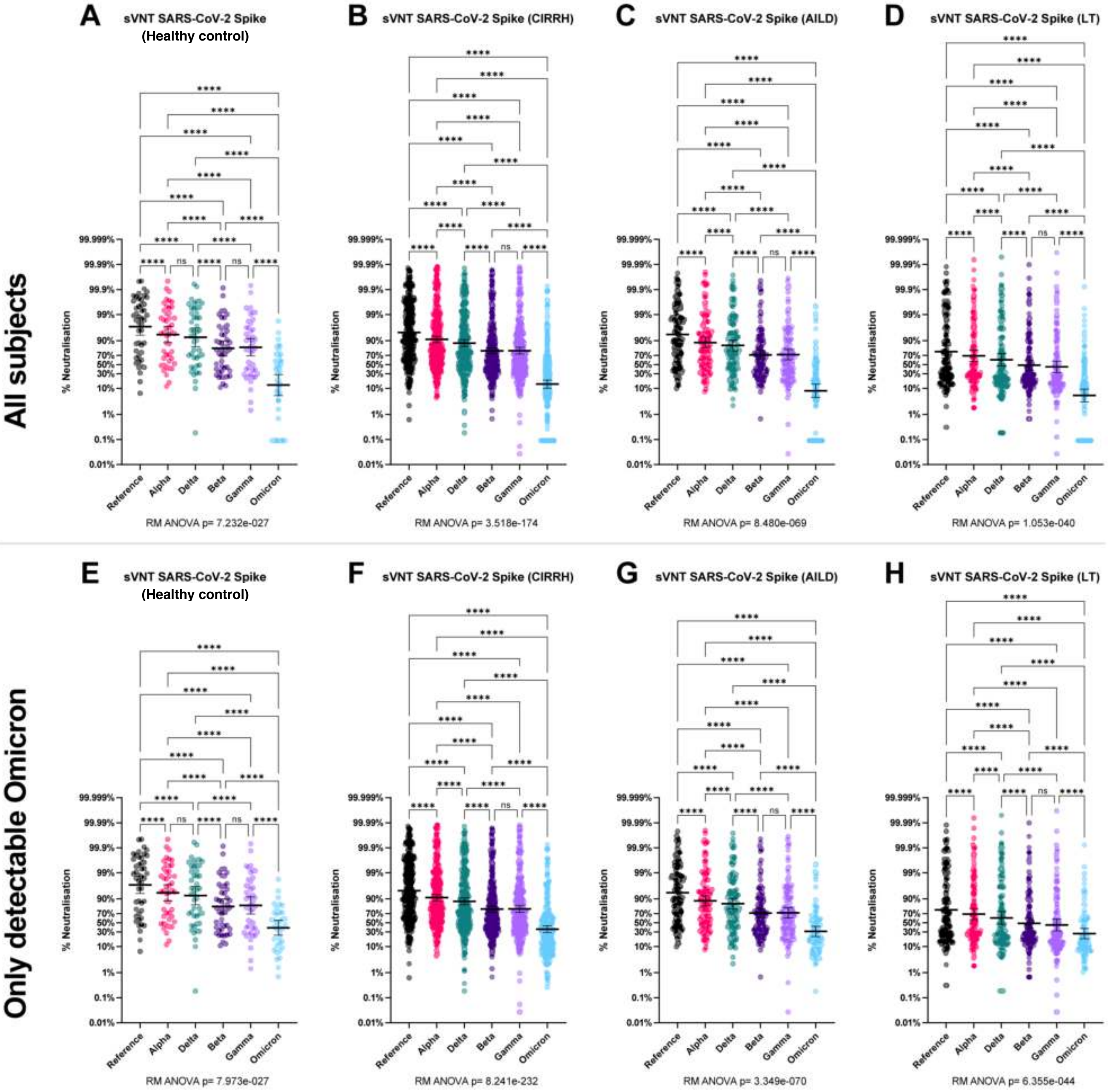

Supplementary Figure 6

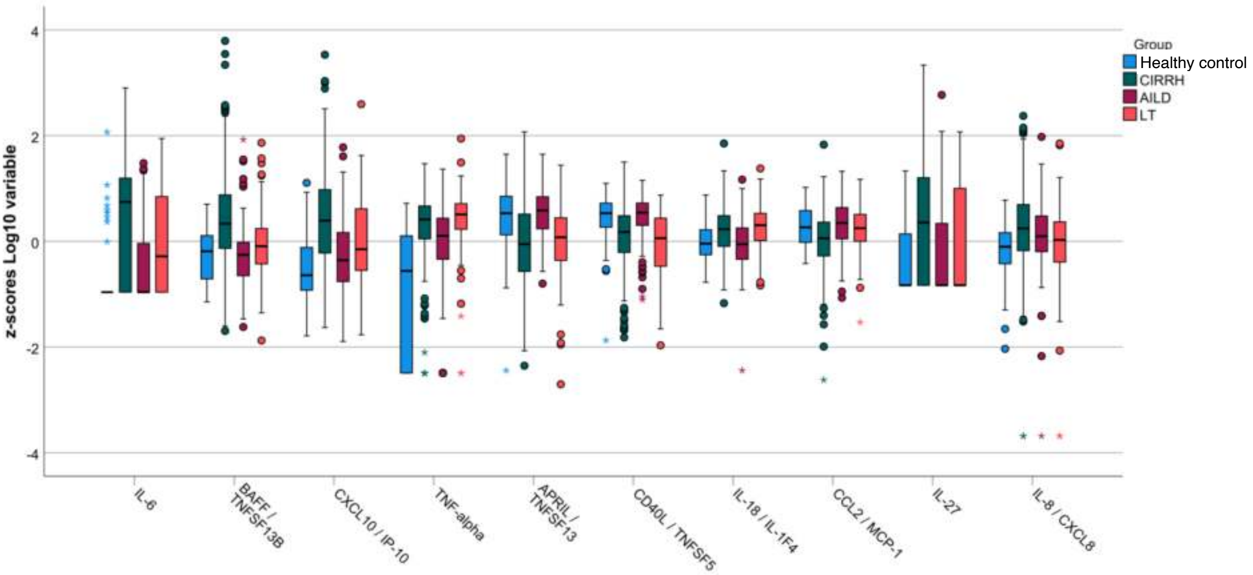

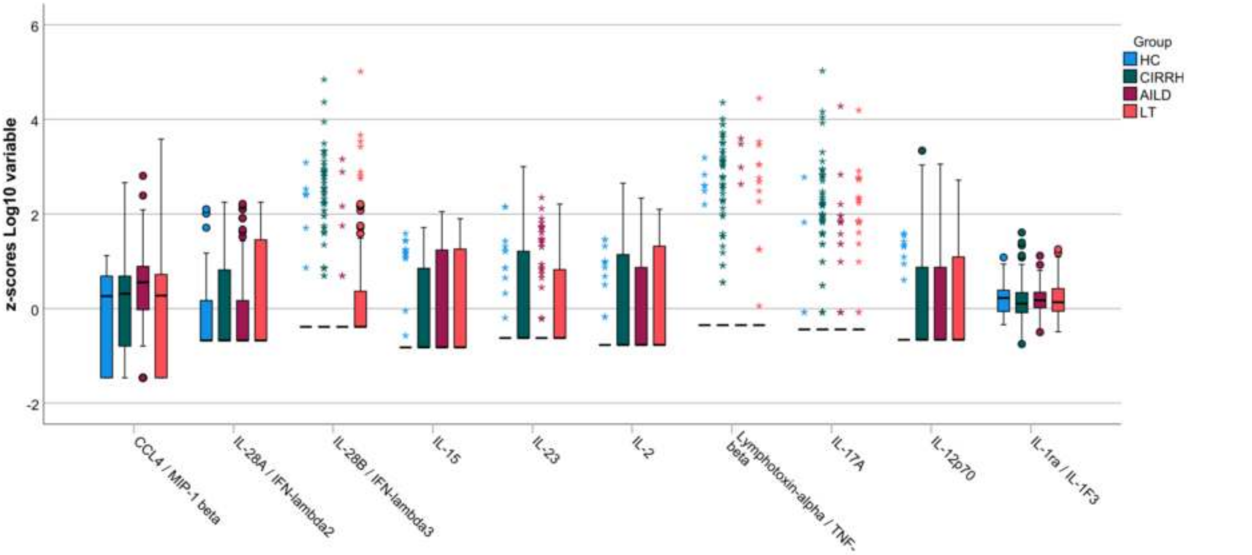

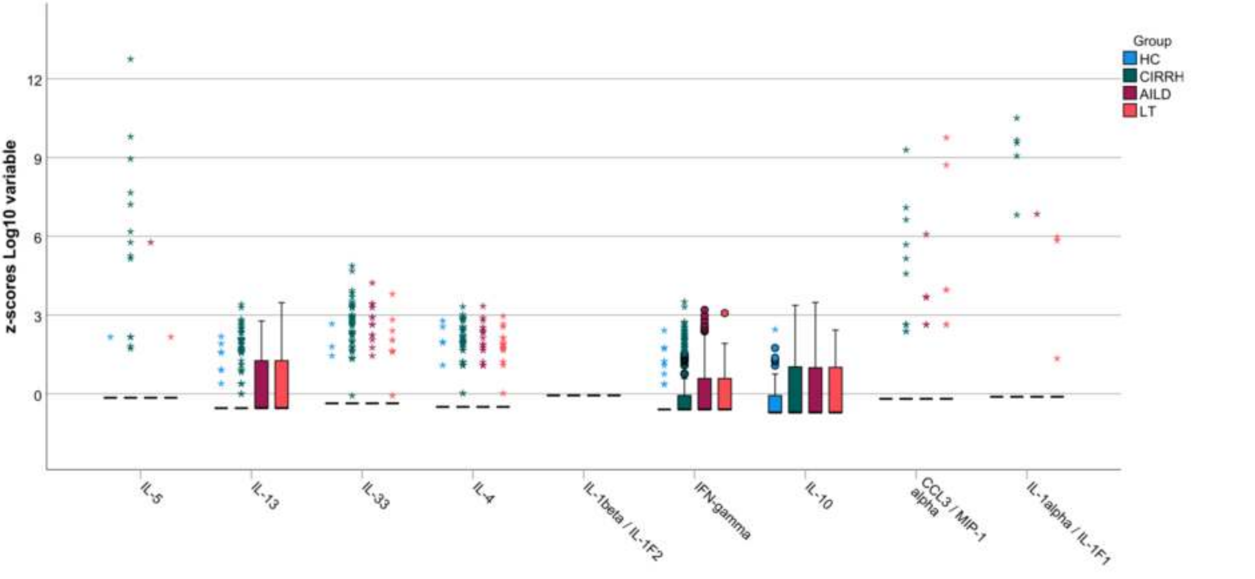

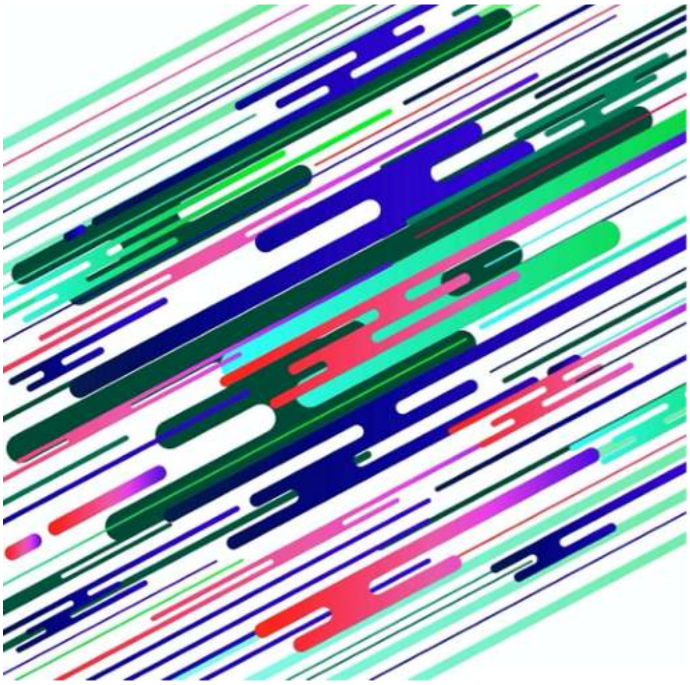

COvid-19 vaccination and Biomarkers in cirrhosis And post-Liver Transplantation (COBALT)

COBALT Study Protocol

Sponsor: EF-CLIF

Protocol authorship: Study Coordinator (SC), Principal Investigator (PI) and Study Steering Committee (SSC, co-PIs)

MAIN STUDY CONTACTS

**Study Coordinator:**	Rajiv Jalan<br>Scientific Director, European Foundation for the Study of Chronic Liver Failure (EF-Clif)<br>r.jalan@ucl.ac.uk
**Principal Investigator:**	Gautam Mehta<br>Honorary Consultant and Associate Professor, UCL Institute for Liver and Digestive Health<br>gautam.mehta@ucl.ac.uk

STUDY STEERING COMMITTEE AND CO-PRINCIPAL INVESTIGATOR(S)

Paolo Angeli	EF-Clif Consortium (Chair) and University Hospital of Padua, pangeli@unipd.it
Luca Belli	ELITA (Board) and Ospedale Niguarda, University of Milano Bicocca, luca.belli@ospedaleniguarda.it
Patrizia Burra	University Hospital of Padua and President 'Fondazione ONLUS Marina Minnaja', burra@unipd.it
Shilpa Chokshi	Acting Director, Institute of Hepatology, Foundation for Liver Research, s.chokshi@researchinliver.org.uk
Christophe Duvoux	ELITA (Board) and Henri Mondor Hospital-Paris Est University, christophe.duvoux@aphp.fr
Paolo Grossi	University of Insubria, paolo.grossi@uninsubria.it
Wojtek Polak	ELITA (Chair) and Erasmus MC Transplant Institute, University Medical Center Rotterdam, w.polak@erasmusmc.nl
Massimo Puoti	Ospedale Niguarda, University of Milano Bicocca, massimo.puoti@unimib.it
Francesco Russo	University Hospital of Padua, and AISF (Board), francescopaolo.russo@unipd.it

SPONSOR

EF-Clif is the research sponsor for this study. The principal contact is Anna Bosch, General Manager of Ef-CLIF, anna.bosch@efclif.com.

Address:	European Foundation for the Study of Chonic Liver Failure,<br>Travessera de Gràcia,<br>11 planta 7a 08021,<br>Barcelona, Spain.	CIF: G-66519000<br>Tel. 932271400

REGIONAL CHAMPIONS AND CO-PRINCIPAL INVESTIGATOR(S)

- **Austria/Switzerland:**
  Vanessa Stadlbauer, Medical University of Graz.
  vanessa.stadlbauer@medunigraz.at

- **France:**
  Laure Elkrief, Trousseau University Hospital, Tours.
  laure_elkrief@yahoo.fr
  Marika Rudler, La Pitié Salpétrière University Hospital, Paris.
  marika.rudler@aphp.fr

- **Germany:**
  Cornelius Engelmann, Charité Universitätsmedizin, Berlin.
  cornelius.engelmann@charite.de

- **Italy:**
  Salvatore Piano, University Hospital of Padua.
  salvatorepiano@gmail.com

- **Belgium/Netherlands:**
  Minneke Coenraad, Leiden University Medical Centre.
  m.j.coenraad@lumc.nl

- **Scandinavia:**
  Karen Louise Thomsen, Aarhus University Hospital, Denmark.
  karethom@rm.dk

- **Spain:**
  Javier Ampuero, Virgen del Rocío University Hospital, Seville.
  javi.ampuero@gmail.com

DATA MANAGEMENT AND STATISTICS

Data custodian: Cristina Sanchez, Ef-Clif, cristina.sanchez@efclif.com

Senior statistician: Alex Amorós, Ef-Clif, alex.amoros@efclif.com

Contents

1.0 Introduction 5
1.1. Background 5
1.2. Biomarkers of immune response 6
2. Study Outline 7
2.1. Study Objectives 7
2.2. Study Design 8
2.3. Study Eligibility 9
2.4. Recruitment Strategies 9
2.5. Consent 9
2.6. Withdrawal of Subjects 10
3. Study Observations and Procedures 10
3.1. Phase 1: Baseline data and biological sampling 10
3.2. Phase 2: Data registry 11
4. Statistics and Data Analysis 12
4.1. Sample Size Considerations 12
4.2. Statistical Analysis Plan 12
5. Regulatory 13
5.1. Clinical Trial Authorisation 13
6. Ethical Considerations 13
6.1. Ethical Approval 13
6.2. Informed Consent Process 13
6.3. Confidentiality 13
7. Sponsor 14
7.1. Indemnity 14
7.2. Funding 14
8. Study Oversight Groups 14
8.1. Study Steering Committee (SSC) 14
9. Data Management 14
10. Publication Policy 15
11. References 15
12. Appendices 16

1.0 Introduction

This is a protocol for a pan-European, longitudinal observational cohort study to interrogate immune responses to vaccination against coronavirus disease-2019 (Covid-19) in patients with cirrhosis, autoimmune liver disease or post-liver transplantation. The study consortium comprises the European Foundation for the Study of Chronic Liver Failure (EF-CLIF), the European Association for the Study of the Liver (EASL), the European Liver and Intestine Transplant Association (ELITA), and the Foundation for Liver Research. This version has been compiled in accordance with SPIRIT guidance.

1.1. Background

The Covid-19 pandemic is the largest public health challenge in living memory. The illness due to Covid-19 has a variable presentation, ranging from asymptomatic to severe pneumonia with multi-organ failure(1). Liver involvement is a frequent feature of Covid-19; amongst hospitalized patients with symptomatic Covid-19 the prevalence of elevated serum transaminases is 14-53%, and degree of elevation is an independent predictor of outcome(2,3). The pattern of liver enzyme abnormality is typically hepatocellular, but the pathobiology of liver dysfunction remains unclear. The novel coronavirus, SARS-CoV-2, has not been shown to directly infect hepatocytes, which typically lack ACE2 receptors, and therefore the liver injury may be a consequence of systemic inflammation(4). Of note, animal models of chronic liver injury suggest that ACE2 expression may be more widely expressed in hepatocytes in the context of liver disease, and thus patients with liver disease may be predisposed to deleterious outcomes from SARS-CoV-2 infection(5,6).

Additionally, patients with chronic liver disease (CLD), including cirrhosis, have dysregulated innate and adaptive immunity, and therefore may be at higher risk of complications from Covid-19 or Covid-19 vaccination. Recent data from Europe, the USA and Asia support deleterious outcomes from Covid-19 in CLD, with a ~3-fold higher risk of mortality with Covid-19 in CLD compared to patients without CLD, and a further increased risk in patients with established cirrhosis (~5-fold)(7-11).

The impact of Covid-19 on liver transplant recipients is also largely unknown, despite the unique immune characteristics of this population. It remains unclear whether immunosuppressive therapies may increase susceptibility to, and severity of, Covid-19, or whether immunosuppression may blunt the systemic inflammation associated with SARS-CoV-2 infection. The same question is applicable to patients with non-cirrhotic autoimmune liver disease. Data from the European Liver and Intestine Transplantation Association (ELITA) and European Liver Transplant Registry (ELTR) suggests increased mortality from Covid-19 in liver transplant recipients, particularly older patients or those with comorbidities. By contrast, use of tacrolimus-based immunosuppression was associated with improved survival(12,13). These factors are therefore also relevant for outcomes following vaccination for Covid-19 in this population.

The advent of approved vaccinations for Covid-19 is an incredibly important and welcome development for these patients and others with chronic illness. However, due to the aforementioned dysregulated immunity in cirrhosis or post-liver transplantation (LT), the efficacy and potential for adverse effects or toxicity from Covid-19 vaccination remains uncertain in these populations.

Immune responses to other vaccines in CLD have been shown to be sub-optimal, and to decline with the stage of the disease(14). Additionally, the immunogenicity of vaccines is also decreased in the post-LT setting, due to maintenance immunosuppression. Although several mechanisms of protection are important following vaccination, the induction of antigen-specific antibody responses is thought to confer the majority of early protective efficacy. Recently, investigators have raised concerns about potential deleterious responses to Covid-19 vaccination if antibody responses are of low quality (i.e. low in titre, avidity, specificity or neutralizing capacity)(15), (see section 1.2). Therefore, since the effectiveness of humoral immune responses to SARS-CoV-2 vaccination in CLD or post-LT is unknown, there is an urgent need to: (i) characterize humoral responses to SARS-CoV-2 vaccination in CLD or post-LT patients, and (ii) establish a prospective registry of vaccinated CLD or post-LT

patients, up to one-year, to explore the clinical efficacy and potential for adverse effects following vaccination in these groups compared to healthy, age-matched controls.

There are three Covid-19 vaccines likely to be widely used for patients with CLD or post-LT in 2021:

(i) **Pfizer BNT162b2** – nanoparticle mRNA encoding full-length SARS-CoV-2 Spike protein

The Pfizer vaccine was the first to be approved by the MHRA (2nd December 2020) and FDA (13th December 2020). These approvals were on the basis of efficacy data reported in a phase 2/3 clinical trial in ~43,000 subjects, either healthy or with stable chronic illness(16). Very few participants (only three) had underlying CLD in this trial. Immunogenicity data from this phase 2/3 trial is yet to be reported.

(ii) **Astra Zeneca ChAdOx1** – replication-deficient simian adenovirus expressing full-length SARS-CoV-2 Spike protein

The Astra-Zeneca vaccine is currently being tested in four, large phase 2/3 randomized controlled trials (RCTs: UK, Brazil, South Africa). Efficacy data has been reported in a pooled interim analysis of these RCTs involving ~11,000 participants(17). Although participants with underlying health conditions are included, there is no record of participants with underlying CLD. Additionally, two dose regimens were analysed (LD/SD and SD/SD) – the standard dose regimen was recently approved in the UK (30th December 2020).

(iii) **Moderna mRNA-1273**-nanoparticle mRNA encoding full-length SARS-CoV-2 Spike protein

The Moderna vaccine has recently been approved for use by the FDA (18th December 2020). Efficacy and safety data from a phase 3 trial of ~30,000 participants have been recently published showing 95% efficacy(18). Liver disease patients were eligible for inclusion, with 96 CLD patients enrolled 96 in the placebo group and 100 in the vaccine group. No case of severe hepatic dysfunction was observed in any of the study participants, although subgroup level data has not been reported.

Additionally, other Covid-19 vaccines are in the process of, or have recently gained, regulatory approval, e.g. Janssen, Novavax.

Therefore, there are currently no data available to assess the efficacy or toxicity of these vaccines in liver disease patients. Despite this, these three vaccines will be widely used across liver disease populations in Europe in 2021. Determination of the relative efficacy and toxicity of vaccines for Covid-19 in CLD and post-LT is an *urgent, unmet clinical need.*

1.2. Biomarkers of immune response

Humoral responses following SARS-CoV-2 vaccination are directed towards the Spike protein, which is the viral antigen delivered by each of the three vaccine candidates. The Spike glycoprotein is an 180kDa protein formed of two functional subunits. The N-terminal S1 subunit contains the receptor binding domain (RBD) required for entry into cells via the host ACE2 receptor. The S2 subunit induces fusion of the viral envelope with host cell membranes and is conserved among coronaviruses. In phase I trials, all three vaccines induced neutralizing antibodies to the spike protein(19,20).

The efficacy of protection offered by neutralising antibodies against Spike glycoprotein has been extrapolated from vaccination data against other coronaviruses. Anti-Spike neutralising IgG confers immunity against SARS and MERS(21,22). Regarding SARS-CoV-2, immune biomarker data from phase 2 studies has only been published for the Astra Zeneca vaccine(23). Total IgG against RBD domain and trimeric (full-length) Spike protein was highly correlated with *ex vivo* live SARS-CoV-2

neutralisation. However, prospective data of correlation between neutralising antibody titres and protection against Covid-19 is not yet available.

As noted in section 1.1, it has been suggested that sub-optimal neutralising antibody responses to Spike antigen may lead to paradoxically enhanced inflammatory responses to subsequent SARS-CoV-2 infection(15). Specifically, sub-optimal antibody may not lead to clearance of the opsonized antibody-virion complex, but instead non-specific uptake of the Fc component of the antibody by the Fcγ receptor (CD16), leading to monocyte-macrophage activation and pro-inflammatory responses (figure 1). Our own data supports pro-inflammatory cell death (pyroptosis) of monocytes as a key process in Covid-19 disease progression. Pyroptosis markers are elevated in Covid-19 and correlate with disease severity. Additionally, monocytes exposed to Spike antigen undergo pyroptosis *ex vivo.* Importantly, this effect is *enhanced* by prior incubation with anti-Spike mAb (Junqueira et al., in submission).

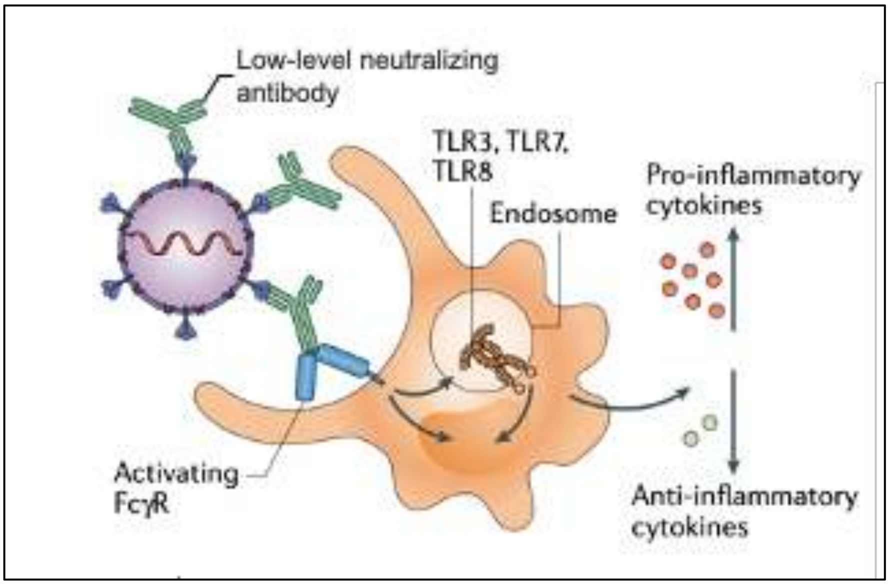

Figure 1 (modified from Iwasaki et al[15]): In the presence of low quality/titre antibodies, inadequate clearance of antibody-virion complexes can lead to engagement of activating FcRs by Fc domains of antibodies. This may lead to upregulation of pro-inflammatory responses and downregulation of anti-inflammatory responses.

2. Study Outline

2.1. Study Objectives

The primary objective of this observational study is:

- to determine if patients with CLD mount comparable humoral immune responses to healthy controls following SARS-CoV-2 vaccination.

Secondary objectives of this observational study are to determine:

- if there are differences in humoral immune response between subgroups with cirrhosis, autoimmune CLD or post-LT,
- the minimum effective level of humoral immunity in cirrhosis, autoimmune CLD or post-LT to provide protection against Covid-19,
- if there are differences in protection from different strains of SARS-CoV-2 (e.g. N501Y variant),
- if there are adverse effects or toxicity from vaccination in the context of underlying cirrhosis, autoimmune CLD or post-LT.

2.2. Study Design

We will undertake an observational, multi-centre study across ~100 European sites, sampling ~5,000 patients with cirrhosis, autoimmune CLD or post-LT for cirrhosis. Additionally, 500 healthy participants will be recruited. Recruitment will be stratified by following subgroups:

- Cirrhosis (staged by Child-Pugh, CP score):
  - CP-A 1,000 patients
  - CP-B 1,000 patients
  - CP-C 1,000 patients
- Autoimmune CLD without cirrhosis (PSC/PBC/AIH): 500 patients
- Post-LT for cirrhosis:
  - 6 months – 5 years post-LT (short): 500 patients
  - >5 years post-LT (long): 500 patients
- Healthy: 500 participants

Each site will be expected to enrol eligible patients at a ratio of 2:2:2:1:1:1:1 according to the seven subgroups above. The study design is pragmatic, to accommodate local vaccination schedules and procedures.

The primary statistical endpoint for this study is to compare the titre of anti-Spike IgG (total) in patients with cirrhosis (combined CP-A, CP-B and CP-C) with healthy participants at 7 weeks (±3 weeks) following final vaccination dose.

Secondary endpoints are divided by phase of study (see section 4.2 Statistical Analysis Plan):

Phase 1 Biological sampling: The primary endpoint will be determined by sampling at 7 weeks (±3 weeks) following final vaccination dose (for either one-dose or two-dose regimens). For secondary endpoints, further optional sampling time points will be at baseline (within 4 weeks prior to initial vaccination dose), and at 32 weeks (±3 weeks) following final vaccination dose.

Phase 2 Data registry: Data for secondary endpoints will be collected up to 12 months following inclusion, for all seven subgroups [CP-A, CP-B, CB-C, autoimmune CLD, post-LT (short), post-LT (long), healthy]. Specific episodes to be collected are: diagnosis of Covid-19 (PCR positive), hospitalisation due to Covid-19, liver-related hospitalisation, liver-related mortality, all-cause hospitalisation, all-cause mortality and incident liver transplantation for patients with CLD.

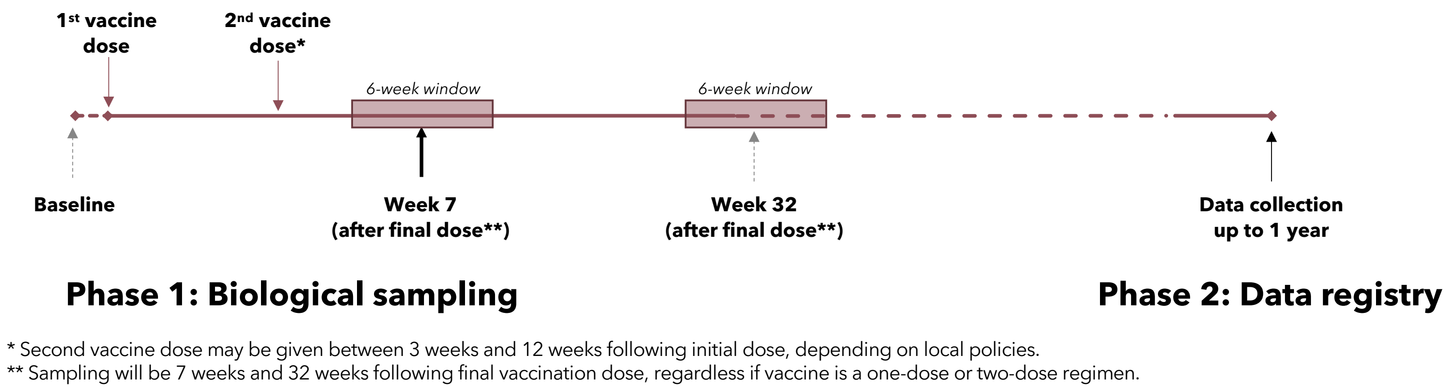

Figure 2: Study outline

2.3. Study Eligibility

All patients with cirrhosis, autoimmune CLD or post-LT for cirrhosis, who are planned to receive vaccination for SARS-CoV-2 will be considered for eligibility into the study. Additionally, healthy adults, with absence of chronic illness, will be considered healthy participants and be eligible for the study. Participants receiving all brands/regimens of SARS-CoV-2 vaccine will be eligible (i.e. Pfizer, Astra-Zeneca, Moderna etc).

Specific inclusion criteria:

i) Participant able to give written informed consent

ii) Diagnosis of:

  a. Cirrhosis (on imaging or liver biopsy), or,

  b. Autoimmune liver disease (PSC, PBC or AIH) without cirrhosis, or

  c. Post-LT for cirrhosis >6 months, or

  d. Healthy participant (absence of severe and uncontrolled cardiac, respiratory, liver, renal or endocrine disease in opinion of PI or sub-I, see appendix 1).

iii) Age >18 years

Specific exclusion criteria:

i) History of Covid-19 (PCR-positive episode)

ii) Participant unable to give written informed consent

iii) Uncontrolled HIV infection

2.4. Recruitment Strategies

i) **Approaching patients in clinic:** Eligible patients may be approached at any point until 10 weeks after final Covid-19 vaccination dose, to allow sampling for the primary endpoint (anti-Spike IgG at 7±3 weeks). Ideally, this will be *prior* to initial vaccination to also allow sampling at baseline (up to 4 weeks prior). Patients will be approached by a member of the hospital research team, will receive a PIS and be given sufficient time to read and discuss the study. Hospital staff attending for vaccination may be recruited as healthy participants.

ii) **Approaching patients by post/telephone:** Where possible, eligible patients may be approached via telephone, email or post, and sent a letter and PIS prior to clinical visit. As above, this may be any point until 10 weeks after final Covid-19 vaccination dose, although ideally prior to initial vaccination. The letter will include contact details for the Principal Investigator at the participating site and may also include contact details for the Research Nurse at the site. This will allow additional time for the patient to consider entry to the study. Hospital staff may be recruited as healthy participants.

2.5. Consent

The principal investigator (or sub-investigator) will consecutively provide an explanation regarding the study to each eligible participant, using the informed consent document. Participants who give written consent will be registered in the electronic case report form (eCRF), and then observations will be initiated. Participant registration will be closed for the entire study at the time the target number of

participants has been enrolled in each of the seven subgroups [CP-A, CP-B, CB-C, autoimmune CLD, post-LT (short), post-LT (long), healthy]. Each participant will receive a unique identification number at the time of registration on the eCRF. The principal investigator will ensure this number is anonymised and maintained with each participant at the time of informed consent, to protect the participant's private information. These unique numbers will be used and not changed throughout the study.

2.6. Withdrawal of Subjects

A participant may voluntarily discontinue participation in this study at any time. This will not affect his/her current or future treatment. The study investigator (or sub-investigator) may also, at his or her discretion, discontinue the subject from participating in this study at any time. If a blood sample has been collected and it is determined that the patient does not meet the inclusion and exclusion criteria for participation, or if the patient withdraws consent from the study, then the study physician must complete the appropriate documentation for sample destruction. It is the responsibility of the study investigator to destroy the sample and to keep a record of that destruction in the study file.

3. Study Observations and Procedures

3.1. Phase 1: Baseline data and biological sampling

Demographics, medical history and medication history

Demographic information to be obtained will include date of birth, gender, sex, height, weight, smoking status, drinking habits, and date of onset or diagnosis of liver disease (if applicable).

Medical history will include previous episodes of verified Covid-19 infection (an exclusion criterion). Additionally, the presence or absence of the following symptoms of decompensated cirrhosis: jaundice, ascites, confusion (hepatic encephalopathy), peripheral oedema, fever or signs of bacterial infection. Past history of gastrointestinal bleeding or hepatocellular cancer will also be recorded. Further clinically significant symptoms or diseases judged by the principal investigator (or sub-investigator) will also be recorded.

Ongoing conditions are considered concurrent medical conditions (see the later section of Concurrent medical conditions).

Medication history will include any immune-active drugs used within 12 months, including corticosteroids or immunosuppressants. Additionally, most recent trough immunosuppression drug monitoring level will be recorded.

Physical examination procedures

A baseline physical examination will optionally consist of the following body systems: cardiovascular system, respiratory system, gastrointestinal system, dermatological system, extremities, musculoskeletal system, nervous system and lymph nodes.

Concomitant medications

Detailed information (drug name, duration and daily dose) will be obtained on all administered medications at the time of enrolment.

Concurrent medical conditions

Concurrent medical conditions are ongoing conditions or diseases that are present at time of enrolment. This will include clinically significant abnormalities observed in laboratory tests, ECG or physical

examination, as judged by the principal investigator (or sub-investigator).

Diary monitoring for adverse effects

For participants recruited prior to the first or second dose of vaccination, they will optionally be asked to complete a paper diary of local or systemic symptoms within 2-weeks of vaccination dose (either first, or second, or both). Local and systemic reactions will be graded according to the FDA Center for Biologics Evaluation and Research (CBER) guidelines on toxicity grading scales for healthy adult volunteers enrolled in preventive vaccine clinical trials(24). These data will be returned to the investigating site (by post or email) by week 24 of the study and will be added to the eCRF (see appendix 3).

Sample collection

Blood markers will be collected for the primary and secondary endpoints of anti-SARS-CoV-2 IgM and IgG levels, as well as for further exploratory endpoints. The following samples will be systematically collected: serum (5ml BD SSTII tubes x2), plasma (4ml BD EDTA tube x2), RNA (3ml Tempus tube x1), DNA (buffy coat from EDTA sample). Samples will be collected and processed according to the laboratory manual and stored at -80°. Samples will be collected according to the schedule shown in figure 2, at baseline (-28 days), week 7 (±21 days) and week 32 (±21 days) following final vaccination (aside from RNA and DNA which are only collected at week 10).

All centres will receive a package containing the necessary tubes and processing instructions (separation, aliquoting, freezing, registration and shipment). Detailed SOPs will be provided for each sample type (see appendix 2). Following the end of the recruitment phase, samples will be shipped to the UCL-Royal Free Hospital Biobank, located at the Royal Free Hospital, Pond Street, London NW3 2QG (Amir Gander, Manager – Tissue Access for Patient Benefit, Email: a.gander@ucl.ac.uk) for centralized storage for centralized storage and additional exploratory testing on remaining samples (including DNA and RNA samples) to investigate and better understand the immune responses to SARS-CoV-2 vaccination in CLD or post-LT. Results from this analysis are to be documented and maintained, but are not necessarily reported as part of this study. Samples can be retained for up to 20 years.

Since the evaluations are not expected to benefit the subject directly or to alter the treatment course, the results of pharmacogenetic, biomarker development, or other exploratory studies will not be placed in the subject's medical record and will not be made available to the subject, members of the family, the personal physician, or other third parties, except as specified in the informed consent.

Samples will be destroyed once all protocol-defined procedures are completed at the end of the storage period, or as appropriate (i.e. the scientific rationale for experimentation with a certain sample type no longer justifies keeping the sample**).**

Clinical laboratory tests

Laboratory parameters will also be collected according to the schedule shown in figure 2. Additionally, the investigator will collect data on haematology (full blood count and coagulation) and biochemistry (liver and renal) profiles at the following time points if available: baseline (-3 months), week 7 (±21 days) and week 32 (±21 days).

3.2. Phase 2: Data registry

Data will be collected from local records at 1-year following study entry, and entered onto the eCRF. Data will be collected for hospitalisations at the study site, due to Covid-19, liver-related or all-cause. diagnosis of Covid-19 (PCR positive), hospitalisation due to Covid-19, liver-related hospitalisation, liver-related mortality, all-cause hospitalisation all-cause mortality and incident liver transplantation.

4. Statistics and Data Analysis

4.1. Sample Size Considerations

Prior data demonstrates patients with cirrhosis have approximately 40% lower IgG titre following pneumococcal vaccine at 6-months, compared to healthy controls(14). The primary statistical endpoint for this study is the compare the titre of anti-Spike IgG (total) in patients with cirrhosis (combined CP-A, CP-B and CP-C) with healthy participants at week 7. Assuming a 20% reduction in anti-Spike IgG in patients with cirrhosis compared with healthy participants at week 7, alpha risk of 0.05 and beta risk of 0.2 in a one-sided test, 362 patients are necessary in the healthy group and 2172 in the cirrhotic group. A drop-out of 5% has been anticipated. Normal values of anti-spike IgG after 28-day Astra Zeneca vaccine administration, in age group 56-69 years, were considered: median 16,170 AU/ml [IQR 10,233-40,353 AU/ml](23).

4.2. Statistical Analysis Plan

A formal statistical analysis plan will be written and agreed with the study steering committees before data lock. The primary statistical endpoint for this study is to compare the titre of anti-Spike IgG (total) in patients with cirrhosis (combined CP-A, CP-B and CP-C) with healthy participants at week 7. Data will be analysed after accrual of each 1,000 patient data sets. Secondary statistical analyses will be sub-divided by phase of study:

Phase 1: Biological sampling

Secondary analyses will include:

- Titre of anti-RBD and anti-Spike (total) IgM and IgG at baseline, week 7 and week 32, between all subgroups (between-group comparisons).

Phase 2: Data registry

Secondary analyses will include:

- Incidence of Covid-19 (symptomatic and PCR-positive) up to 1 year
- Hospitalisations (all cause, Covid-19 related and liver-related) up to 1 year
- Mortality (all cause, Covid-19 related and liver-related) up to 1 year
- Incidence of liver transplantation

Covariates for primary and secondary analyses will include: age, stage of liver disease, previous Covid-19 infection (symptomatic or on serology), type (brand of vaccine), duration post-LT, degree of immunosuppression (drug regimen, trough drug level).

Discrete variables will be shown as counts(percentage), continuous variables normally distributed as mean (SD) and not-normally distributed will be summarized by median (interquartile range; IQR). In univariate statistical comparisons, the chi-square test will be used for categorical variables, whereas the Student t-test or analysis of variance will be used for normal continuous variables and the Wilcoxon or the Kruskal-Wallis test will be used for continuous variables nor normally distributed.

Logistic binary regression will be carried out to find differences between healthy and cirrhotic groups due to COVID-19 and hospitalizations. Covariables showing a clinical and statistical significance or participating as a confounding factor for the variable of interest will be included in the final models.

The proportional-hazard model for competing risks proposed by Fine and Gray will be used to identify differences between groups of study and mortality, caused by Covid-19 and liver disease. Independent covariates will be included in the models when show statistical significance or confusion. This model will be chosen to account the different causes of mortality and liver transplantation as an event competing with the primary event in each case.

In general, missing data will not be imputed. However, if we can consider missing values as random, a multiple imputation based on a mixed model including all potential factors for multivariate analysis will be carried out.

In all statistical analysis significance will be set at P<0.05. Analyses will be done with SAS V9.4 statistical package. Interim analyses will be performed after recruitment of 1000, 2000, 3000 and 4000 participants.

5. Regulatory

5.1. Clinical Trial Authorisation

This study is not considered to be a clinical trial of a medicinal product (CTIMP), so clinical trial authorisation from the UK and EU is not applicable.

6. Ethical Considerations

The study will be conducted in accordance with the recommendations for physicians involved in research on human participants adopted by the 18th World Medical Assembly, Helsinki 1964 as revised and recognised by governing laws and EU Directives. Each participant's consent to participate in the study should be obtained after a full explanation has been given. The right of the participant to refuse to participate in the study without giving reasons must be respected.

6.1. Ethical Approval

Ethical approval for this study will be sought through the UK and EU REC processes.

6.2. Informed Consent Process

Informed consent is a process that is initiated prior to an individual agreeing to participate in a study and continues throughout the individual's participation. In obtaining and documenting informed consent, the investigator should comply with applicable regulatory requirements and should adhere to the principles of GCP.

Discussion of objectives, risks and inconveniences of the study and the conditions under which it is to be conducted are to be provided to the participant by appropriately delegated staff with knowledge in obtaining informed consent with reference to the patient information leaflet. This information will emphasise that participation in the trial is voluntary and that the participant may withdraw from the trial at any time and for any reason. The participant will be given the opportunity to ask any questions that may arise and provided the opportunity to discuss the study with family members, friend or an independent healthcare professional outside of the research team and time to consider the information prior to agreeing to participate.

6.3. Confidentiality

All investigators will preserve the confidentiality of participants taking part in the study. The investigator must ensure that participant's anonymity will be maintained and that their identities are

protected from unauthorised parties. On CRFs participants will not be identified by their names, but by an identification code.

7. Sponsor

The sponsor of this study is EF-CLIF. All members of the consortium have been delegated specific duties by the Sponsor, as documented in Study Steering Committee minutes.

7.1. Indemnity

EF-CLIF's public and professional indemnity insurance policy provides an indemnity to EF-CLIF employees for their potential liability for harm to participants during the conduct of the research.

7.2. Funding

The study is funded by EF-CLIF and the Foundation for Liver Research.

8. Study Oversight Groups

The day-to-day management of the study will be co-ordinated through the Study Management Group and the Study Steering Committee.

8.1. Study Steering Committee (SSC)

The SSC is responsible for overseeing progress of the study, including both the clinical and practical aspects. The Chair of the SSC will be the Study Coordinator. The membership of the SSC is outlined on pages 2-3.

9. Data Management

Participant data will be entered remotely at site and retained in accordance with the Data Protection Act (1988). The PI is responsible for ensuring the accuracy, completeness, and timeliness of the data entered. The participant data is pseudo anonymised by assigning each participant a participant identifier code which is used to identify the participant during the study and for any participant-specific clarification between EF-CIF Data Management Center and site. The site retains a participant identification code list which is only available to site staff.

The Informed Consent Form will specify the participant data to be collected and how it will be managed or might be shared; including handling of all Patient Identifiable Data (PID) and sensitive PID adhering to relevant data protection law.

Trained personnel with specific roles assigned will be granted access to the eCRF. Completion guidelines will be provided to the investigator sites to aid data entry of participant information.

Only the Investigator and personnel authorised by them should enter or change data in the eCRFs. When requested, laboratory data must be transcribed, with all investigator observations entered into the eCRF. The original laboratory reports must be retained by the Investigator for future reference.

Data queries will either be automatically generated within the eCRF, or manually raised by the study team, if required. All alterations made to the eCRF will be visible via an audit trail which provides the identity of the person who made the change, plus the date and time.

At the end of the study after all queries have been resolved and the database frozen, the PI will confirm the data integrity by electronically signing all the eCRFs.

10. Publication Policy

Data from all centres will be analysed together and published as soon as possible.

Individual investigators may not publish data concerning their patients that are directly relevant to questions posed by the study until the Study Steering Committee (SSC) has published its report. The SSC will form the basis of the Writing Committee and advise on the nature of publications. All publications shall include a list of investigators, and if there are named authors, these should include the Study Coordinator, Co-Investigators, and Statistician(s) involved in the trial. Named authors will be agreed by the SC and PIs. If there are no named authors, then a 'writing committee' will be identified.

11. References

1. Huang C et al. Clinical features of patients infected with 2019 novel coronavirus in Wuhan, China. *Lancet* 2020;395:497-506.
2. Hao SR et al. Liver Enzyme Elevation in Coronavirus Disease 2019: A Multicenter, Retrospective, Cross-Sectional Study. *Am J Gastroenterol* 2020;115:1075-1083.
3. Yip TC et al. Liver injury is independently associated with adverse clinical outcomes in patients with COVID-19. *Gut* July 8 2020; doi: 10.1136/gutjnl-2020-321726.
4. Bangash MN et al. SARS-CoV-2: Is the liver merely a bystander to severe disease? *J Hepatol* 2020;73:995-6.
5. Paizis G et al. Chronic liver injury in rats and humans upregulates the novel enzyme angiotensin converting enzyme 2. *Gut* 2005;54:1790-96.
6. Herath CH, et al. Upregulation of hepatic angiotensin-converting enzyme 2 (ACE2) and angiotensin-(1–7) levels in experimental biliary fibrosis. *J Hepatol* 2007;47:387-95.
7. Singh S et al. Clinical characteristics and outcomes of Coronavirus disease 2019 among patients with pre-existing liver disease in the United States: a multicenter research network study. *Gastroenterology* 2020;159:768-71.
8. Moon AM et al. High mortality rates for SARS-CoV-2 infection in patients with pre-existing chronic liver disease and cirrhosis: Preliminary results from an international registry. *J Hepatol* 2020;73:705-8.
9. Avarone M et al. High rates of 30-day mortality in patients with cirrhosis and COVID-19. J Hepatol 2020;73:1063-71.
10. Sarin SK et al. Pre-existing liver disease is associated with poor outcome in patients with SARS CoV2 infection; the APCOLIS study (APASL COVID-19 liver injury spectrum study). *Hepatol Int* 2020;14:690-700.
11. Shalimar et al. Poor outcomes in patients with cirrhosis and Corona Virus Disease-19. *Indian J Gastroenterol* 2020;39;285-91.
12. Belli LS et al. COVID-19 in liver transplant recipients: preliminary data from the ELITA/ELTR registry. *Lancet Gastroenterol Hepatol* 2020;8:724-5.
13. Belli LS et al. Protective role of tacrolimus, deleterious role of age and comorbidities in liver transplant recipients with Covid-19: results from the ELITA/ELTR multi-center European study. *Gastroenterology* 2020; doi: 10.1053/j.gastro.2020.11.045.
14. McCashland TM et al. Pneumococcal vaccine response in cirrhosis and liver transplantation. *Journal of Infectious Diseases* 2000;181:757–60.
15. Iwasaki A, Yang Y. The potential danger of suboptimal antibody responses in COVID-19. *Nat Immunol* 2020;20:339-41.
16. Polack FP et al. Safety and efficacy of the BNT162b2 mRNA Covid-19 vaccine. *NEJM* December 10 2020, doi:10.1056/NEJMoa2034577.
17. Voysey M et al. Safety and efficacy of the ChAdOx1 nCoV-19 vaccine (AZD1222) against SARS-CoV-2: an interim analysis of four randomised controlled trials in Brazil, South Africa, and the UK. *Lancet* December 8 2020, doi: 10.1016/S0140-6736(20)32661-1.
18. Baden LR et al. Efficacy and Safety of the mRNA-1273 SARS-CoV-2 Vaccine. *NEJM* December 30, 2020, doi:10.1056/NEJMoa2035389.
19. Zhu F-C et al. Immunogenicity and safety of a recombinant adenovirus type-5-vectored COVID-19 vaccine in healthy adults aged 18 years or older: a randomised, double-blind, placebo-controlled, phase 2 trial. Lancet 2020;396:479-88.

20. Folegatti PM et al. Safety and immunogenicity of the ChAdOx1 nCoV-19 vaccine against SARS-CoV-2: a preliminary report of a phase 1/2, single-blind, randomised controlled trial. Lancet2020;396:467-78.
21. Martin J et al. A SARS DNA vaccine induces neutralizing antibody and cellular immune responses in healthy adults in a Phase I clinical trial. *Vaccine* 2008;26:6338-43.
22. Folegatti PM et al. Safety and immunogenicity of a candidate Middle East respiratory syndrome coronavirus viral-vectored vaccine. *Lancet Infect Dis* 2020;20:816-826.
23. Ramasamy MN et al. Safety and immunogenicity of ChAdOx1 nCoV-19 vaccine administered in a prime-boost regimen in young and old adults (COV002): a single-blind, randomised, controlled, phase 2/3 trial. *Lancet*, November 18 2020, doi: 10.1016/S0140-6736(20)32466-1.
24. US Food and Drug Administration. Guidance for industry: toxicity grading scale for healthy adult and adolescent volunteers enrolled in preventive vaccine clinical trials. Rockville, MD: Center for Biologics Evaluation and Research; September 2007.

12. Appendices

Appendix 1: Definitions of chronic illness (for identification of healthy participants)

As noted in section 2.3, healthy participants will be recruited according to the absence of uncontrolled and severe cardiac, respiratory, liver, renal or endocrine disease, according to the discretion of the PI or sub-I. Definitions of severe disease are provided below:

Severe heart disease

- Unstable angina or recent myocardial infarction (NSTEMI or STEMI)
- Congenital heart disease requiring lifelong follow-up
- Congestive heart failure meeting criteria ≥ New York Heart Association Class III or Class C

Severe respiratory disease:

- Chronic obstructive pulmonary disease (COPD) requiring long-term oxygen therapy
- Interstitial lung fibrosis
- Asthma with previous severe or life-threatening attack or requiring critical care admission

Severe liver disease

- Advanced liver fibrosis or cirrhosis

Severe renal disease

- Chronic kidney disease with requirement for dialysis

Severe endocrine disease

- Uncontrolled diabetes mellitus (symptomatic or recent HbA1c >64 mmol/L)
- Uncontrolled (symptomatic) thyroid disease

Appendix 2: Sample collection protocols

Serum: Collect venous blood in 1x SST tubes (8.5 ml). Tube should be inverted five times, allowed 30 minutes clotting time at room temperature, and centrifuged* for 15 minutes at 4ºC at 1300 RCF (g) in a swing bucket centrifuge. Transfer the collected serum into provided cryovials in aliquots (x6) of 500µl and store at -80ºC.
*Tubes should be centrifuged no longer than 4 hours after collection.

Plasma: Collect venous blood in 1x EDTA tube (10 ml). The tube should be centrifuged for 15 minutes at 4ºC at 1300 RCF (g) in a swing bucket centrifuge. Transfer the plasma into the provided cryovials in aliquots (x6) of 500µl and store at -80ºC.

RNA sample (only at week 7 visit): Collect ~3 mL of blood directly into Tempus™ Blood RNA Tube. Shake the tube vigorously for 10-20 sec (favouring cell lysis) and store at -80ºC as soon as possible.

DNA sample (only at week 7 visit): Collect the buffy coat from the EDTA tube (the white thin cell fraction (~0.5 ml) just above the red cell cushion using a Pasteur plastic pipette) and transfer to provided cryovial and store at -80ºC.

Appendix 3: Sample diary for adverse effects monitoring (attached)

COBALT Study - Symptom Diary **Participant ID:**

Dr Gautam Mehta, Royal Free London

INFORMATION ON SYMPTOM MONITORING – PLEASE READ CAREFULLY

- **Please fill in this diary *only on the days you have symptoms during the 2 weeks after your Covid-19 vaccination*.**
  If you don't have symptoms, you don't need to complete any pages. Please complete one page for each day you have symptoms. You can return this diary when you next see the hospital research team. Or, if you are happy to do so, you could return the diary by email. We may also contact you to discuss the results of the diary.

- **We are looking to record the following symptoms:**
  - **Symptoms at the injection site: pain, redness (record the size), swelling.**
  - **Other general (systemic) symptoms: vomiting, diarrhoea, headache, fatigue, chills, muscle pain, joint pains.**

- **Unless stated, please try and grade them:**
  - **MILD (doesn't interfere with activities), MODERATE (some interference with activities), SEVERE (prevents daily routine activities). Also, mark in the comments if you needed to see your doctor for any of these symptoms.**

COBALT Study Symptom Diary	Patient ID:	Date:

Symptoms *at the injection site:*	Severity			Comments
	Mild	Moderate	Severe	
**Pain**				
**Redness** (mild: <2cm, moderate: 2-5cm, severe: >5cm)				
**Swelling** (mild: <2cm, moderate: 2-5cm, severe: >5cm)				

General symptoms	Severity			Comments
	Mild	Moderate	Severe	
**Vomiting** (mild: 1-2/day, moderate: >2/day, severe: needs medical attention)				
**Diarrhoea** (mild: 2-3/day, moderate: 4-5/day, severe: >6/day)				
**Headache**				
**Fatigue / tiredness**				
**Chills**				
**New or worsened muscle pain**				
**New or worsened joint pain**				

COBALT

COvid-19 vaccination and Biomarkers in cirrhosis And post- Liver Transplantation (COBALT)

Statistical Analysis Plan version 2.0

Study Coordinator: Rajiv Jalan

Principal Investigator: Gautam Mehta

Statistician: Eva Uson

Data Custodian: Cristina Sanchez

Name	Date	Signature
Eva Uson	08/06/22	
Gautam Mehta	14/5/22	
Cristina Sanchez	16/5/22	
Maria Pilar Ballester	16/5/22	
Antonio Riva	15/5/22	
Rajiv Jalan	15/5/22	

Abbreviations

**AE**	Adverse Event
**AILD**	Autoimmune liver disease
**CLD**	Chronic liver disease
**CP**	Child-Pugh
**CRF**	Case Report Form
**CSP**	Clinical Study Protocol
**DNA**	Deoxyribonucleic Acid
**ICH**	International Council for Harmonisation
**IQR**	Interquartile range
**mRNA**	messenger Ribonucleic Acid
**PBC**	Primary biliary cirrhosis
**Post-LT**	Post-liver transplantation
**PSC**	Primary sclerosing cholangitis
**SD**	Standard Deviation

Table of Contents

1. INTRODUCTION 4

2. STUDY OBJECTIVES 4

3. ENDPOINTS...5

3.1. PRIMARY ENDPOINTS 5
3.2. SECONDARY ENDPOINTS .... **Error! Bookmark not defined.**
3.3. TERTIARY ENDPOINTS .... **Error! Bookmark not defined.**

4. OVERALL STUDY DESIGN 6

4.1. OVERVIEW OF STUDY DESIGN 6

5. DATA SETS INCLUDED IN THE ANALYSIS 7

6. STATISTICAL ANALYSIS PLAN 7

6.1. CHANGES IN THE PLANNED ANALYSES 7
6.2. BLIND REVIEW.. 7
6.3. DEFINITIONS 7
6.4. DESCRIPTIVE ANALYSIS 8
6.5. PATIENT DATA LISTING 8
6.6. DEMOGRAPHIC AND OTHER CHARACTERISTICS 8
6.7. MEDICAL HISTORY 8
6.8. PHYSICAL EXAMINATION 8
6.9. CONCURRENT MEDICAL CONDITIONS 8
6.10. ENDPOINTS ANALYSIS 8
6.10.1. PRIMARY ENDPOINTS (these are copied from above) 8
6.1.2 SECONDARY ENDPOINTS.. **Error! Bookmark not defined.**
6.1.3 TERTIARY ENDPOINTS .... **Error! Bookmark not defined.**
6.11. CONCOMITANT MEDICATIONS 10
6.12. CLINICAL LABORATORY TESTS 10
6.13. LEVEL OF SIGNIFICANCE AND MULTIPLE COMPARISONS 10
6.14. ADJUSTMENT FOR COVARIATES 10
6.15. HANDLING OF DROPOUTS AND MISSING DATA 10
6.16. MULTICENTER STUDIES... 10
6.17. EXAMINATION OF SUBGROUPS 11
6.18. INTERIM ANALYSIS .... **Error! Bookmark not defined.**

7. LIST OF OUTPUTS 11

7.1. TABLES AND GRAPHS TO BE PRODUCED FOR THE CLINICAL STUDY REPORT 11
7.2. LISTINGS OF INDIVIDUAL PATIENT DATA AND OTHER INFORMATION TO BE PRODUCED FOR THE CLINICAL STUDY REPORT 13

8. REFERENCES...14

APPENDICES 15

1. INTRODUCTION

The COBALT study is a prospective, observational cohort study designed to interrogate immune responses in patients with chronic liver disease and post-liver transplantation, in the context of Covid-19 infection and vaccination.

This Statistical Analysis Plan (SAP) is based on the Clinical Study Protocol (CSP) Versions 2.3 (27th April 2021), 2.4 (27th August 2021) and 2.5 (20th October 2021).

This SAP outlines the pre-planned analyses that will form the core outputs from the COBALT study. Additional ancillary studies are also planned, but are not covered in this SAP.

2. QUESTIONS TO ADDRESS AND STUDY OBJECTIVES

The study is designed to answer a series of key questions:

I. Do patients with CLD or post-LT have comparable immune responses to healthy controls following Covid-19 vaccination?
II. Is there comparable durability of immune response to Covid-19 vaccination in patients with CLD or post-LT compared to healthy controls?
III. What are the risk factors associated with an altered immune response to covid-19 vaccine in patients with CLD or post-LT patients?
IV. Is there a minimum effective level of immunity (measured by humoral assays or otherwise) to provide protection against Covid-19 in patients with CLD or post-LT?
V. Are there demographic, clinical, drug-related, immunological, genetic, transcriptomic or biochemical predictors of acute decompensation or mortality in patients with CLD in the Covid-19 pandemic/post-pandemic era?

To address these questions the objectives of the study are the following:

I. To compare concentrations of anti-Spike IgM and IgG (total) and anti-RBD IgM and IgG (total) in patients with CLD or LT and healthy participants and between all subgroups (between-group comparisons) at week 7 following second and third vaccine doses,

II. To compare concentrations of anti-Spike IgM and IgG (total) and anti-RBD IgM and IgG (total) in patients with CLD or LT and healthy participants at week 7 following second vaccine dose, and week 30-34 following second vaccine dose (prior to 3rd booster dose),

III. To assess and compare any effect of the covid-19 vaccine on pre-existing or novel cross-reactive IgG and IgM directed against Spike from other known human coronaviruses, and correlate IgG and IgM reactivity against SARS-CoV-2 Spike and the magnitude of these possible cross-reactive responses in patients with CLD or LT and healthy participants at week 7 following second vaccine dose

IV. To assess and compare the effect of covid-19 vaccination on the antibody-dependent viral neutralisation against the reference (vaccine) strain SARS-CoV-2 Spike and RBD (as surrogate index of protection) in patients with CLD or LT and healthy participants at week 7 following second vaccine dose

V. To assess and compare the effect of covid-19 vaccination on the antibody-dependent viral neutralisation against Spike from SARS-CoV-2 Alpha, Beta, Gamma, Delta and Omicron variants (as surrogate index of acquired cross-protection) in patients with CLD or LT and healthy participants at week 7 following second vaccine dose

VI. To correlate measures of IgG or IgM antibodies and antibody-dependent viral neutralisation across viral types and SARS-CoV-2 variants in patients with CLD or LT and healthy participants at week 7 following second vaccine dose

VII. To compare demographic, clinical, drug-related, immunological, genetic, transcriptomic or biochemical factors between patients that do and do not respond to vaccination,

VIII. To correlate geometric mean concentrations of anti-Spike IgM and IgG and anti-RBD IgM and IgG against the reference viral strain, and levels of protective neutralisation or cross-neutralisation against the reference viral strain or the five variants of interest with incidence of Covid-19 cases in each subgroup, possibly identifying cases due to infection with different variants

IX. To perform statistical modelling of antibody decay over time in individuals who are sampled at multiple time points

X. To establish predictive models based on the use of all immunological and serological/functional parameters collected in order to assess which immunological variables may be linked to different levels of vaccine efficacy and protection against infection and/or hospital admission (the patient cohort at hand can be 70:30 or 80:20 split in training and validation subsets, to establish and test the models)

XI. To apply multivariable data analysis strategies to identify differential response profiles based on immunological, serological and clinical characteristics

XII. To compare demographic, clinical, drug-related, immunological, genetic, transcriptomic or biochemical factors between patients that require liver-related hospitalisation (acute decompensation), or who undergo liver transplantation or death during 1-year follow-up.

3. STUDY ENDPOINTS

3.1. LISTED ENDPOINTS

- Geometric mean concentration of anti-Spike IgM and IgG (total) and anti-RBD IgM and IgG (total) in patients with CLD or LT and healthy participants at week 7 and week 30-34 (before the 3rd vaccine dose) following second vaccine dose, and modelling of antibody decay over time.
- Immunogenicity of second and third doses booster vaccination (changes in anti-Spike IgM and IgG and anti-RBD IgM and IgG as pre/post analysis) in all subgroups
- Correlation of geometric mean concentrations of anti-Spike IgM and IgG and anti-RBD IgM and IgG with incidence of Covid-19 cases in each subgroup (needs to be adjusted for local incidence of Covid-19 cases at each timepoint and geographic region)
- Predictors of vaccine immunogenicity via univariable and multivariable analysis of all variables
- Predictors of hospitalisations, liver transplantation and mortality via univariable and multivariable analyses of all variables
- Assessment of antibody cross-reactivity against other human coronaviruses and antibody-dependent cross-protection against SARS-CoV-2 variants other than the vaccine reference strain, as induced or influenced by covid-19 vaccination

3.2 SAMPLE SIZE CALCULATION

The sample size calculation is based on comparison of titre of anti-Spike IgG (total) in patients with cirrhosis (combined CP-A, CP-B and CP-C) with healthy participants at week 7 following Covid-19 vaccination. Prior data demonstrates patients with cirrhosis have approximately 40% lower IgG titre following pneumococcal vaccine at 6-months, compared to healthy controls(1). Assuming a 20% reduction in anti-Spike IgG in patients with cirrhosis compared with healthy participants at week 7, 5% type-1 error risk (alpha=0.05) and 20% type-2 error risk (power=0.8) in a one-sided test, 362 patients are necessary in the healthy group and 2172 in the cirrhotic group. A drop-out of 5% has been anticipated. Normal values of anti-spike IgG after 28-day Astra Zeneca vaccine administration, in age group 56-69 years, were considered: median 16,170 AU/ml [IQR 10,233-40,353 AU/ml](2).

4. OVERALL STUDY DESIGN

4.1. OVERVIEW OF STUDY DESIGN

COBALT is an observational, multi-centre study across ~5000 European sites, sampling ~1000 patients with cirrhosis, autoimmune CLD or post-LT for cirrhosis. Additionally, ~500 healthy participants will be recruited.

Selection of study population: Inclusion/exclusion criteria:

The inclusion criteria of patients are:

i) Participant able to give written informed consent
ii) Diagnosis of:
    a. Cirrhosis (on imaging or liver biopsy), or,
    b. Autoimmune liver disease (PSC, PBC or AIH) without cirrhosis, or
    c. Post-LT for cirrhosis >6 months, or
    d. Healthy participant (absence of severe and uncontrolled cardiac, respiratory, liver, renal or endocrine disease in opinion of PI or sub-I, see appendix 1).
iii) Age >18 years

The exclusion criteria are:

i) History of Covid-19 (PCR-positive episode)
ii) Participant unable to give written informed consent
iii) Uncontrolled HIV infection

Figure 1. Study outline

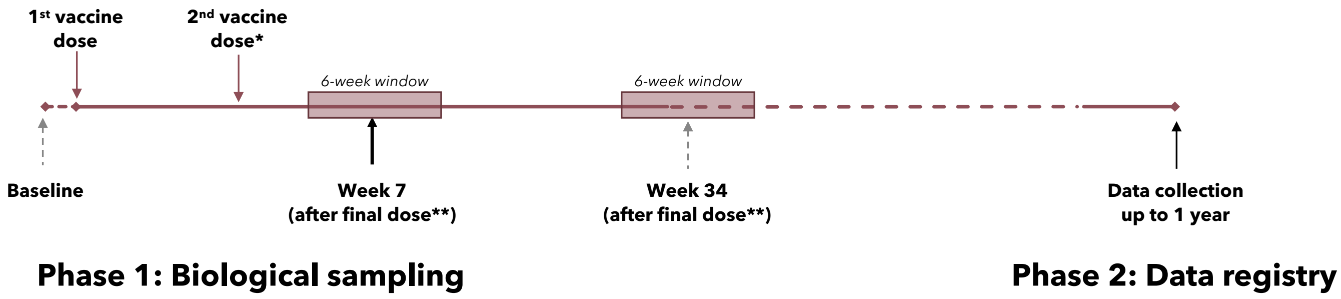

* Second vaccine dose may be given between 3 weeks and 12 weeks following initial dose, depending on local policies.
** Sampling will be 7 weeks and 34 weeks following final vaccination dose, regardless if vaccine is a one-dose or two-dose regimen.

5. DATA SETS INCLUDED IN THE ANALYSIS

All patients with cirrhosis, autoimmune CLD or post-LT for cirrhosis, who are planned to receive vaccination for SARS-CoV-2 will be considered for eligibility into the study. Additionally, healthy adults, with absence of chronic illness, will be considered healthy participants and be eligible for the study. Participants receiving all brands/regimens of SARS-CoV-2 vaccine will be eligible (i.e. Pfizer, Astra-Zeneca, Moderna etc).

6. STATISTICAL ANALYSIS PLAN

The planned tables and listings are presented in *Section 7*.

Statistical analyses will be carried out using SAS v 9.4 or a later version, R v 4.1.0 or a later version, SPSS v26/27 or SIMCA v15/v17 depending on package availability and functionality.

Study days are numbered relative to the baseline, which is considered as the number of days following second dose of vaccination.

Tables, figures, and listings to be presented will follow the standards found in *Sections 14* and *16.2* from ICH E3.

6.1. CHANGES IN THE PLANNED ANALYSES

No changes have been made since the definition of the CSP. If any change in the planned analysis should happen, a proper amendment would be presented specifying the related variables, the new analysis approach, and the theoretical and clinical reasoning behind such decision.

6.2. BLIND REVIEW

N/A

6.3. DEFINITIONS

<u>Duration post-LT</u>

Duration post-LT is derived as follows:

Duration post-LT (months)= (Baseline visit date - Date of liver transplantation +1) / 30.4375

6.4. DESCRIPTIVE ANALYSIS

Discrete variables will be shown as counts (percentage), continuous variables normally distributed as mean (SD) and not-normally distributed will be summarized by median (interquartile range; IQR). In univariable statistical comparisons, (1) associations between categorical variables will be tested using the Pearson's Chi-Square test or Log-linear models depending on data complexity; (2) the Student t-test or Analysis of Variance will be used for normal or normal-transformed continuous variables; (3) the Wilcoxon-Mann-Whitney or the Kruskal-Wallis tests will be used for continuous variables not normally distributed. (4) Predictive assessments will be considered using appropriate GLM models. (5) Bi-variate correlations will be evaluated using the Spearman's rank correlation coefficient (for linearity-independent monotonic relationships) or the Pearson's product-moment correlation coefficient (for linear relationships).

Collected data may require transformation prior to analysis. Whenever normalisation by transformation is applicable (either directly or through GLM link functions), methods based on the Normal distribution should be preferred.

6.5. PATIENT DATA LISTING

Main data collected in the Case Report Form (CRF) will be listed. Listings will be sorted by patient number and treatment group. Refer to *Section 7.2* for more specifications.

6.6. DEMOGRAPHIC AND OTHER CHARACTERISTICS

Demographic information to be obtained will include date of birth, gender, ethnicity, smoking status, drinking habits, and date of onset or diagnosis of liver disease and aetiology (if applicable).

6.7. MEDICAL HISTORY

Medical history will include previous episodes of verified Covid-19 infection (an exclusion criterion). Additionally, the presence or absence of the following symptoms of decompensated cirrhosis: jaundice, ascites, confusion (hepatic encephalopathy), peripheral oedema, fever or signs of bacterial infection. Past history of gastrointestinal bleeding or hepatocellular cancer will also be recorded. Further clinically significant symptoms or diseases judged by the principal investigator (or sub-investigator) will also be recorded.

Ongoing conditions are considered concurrent medical conditions (see the later section of Concurrent medical conditions).

Medication history will include any immune-active drugs used within 12 months, including corticosteroids or immunosuppressants. Additionally, most recent trough immunosuppression drug monitoring level will be recorded.

6.8. PHYSICAL EXAMINATION

A baseline physical examination will optionally consist of the following body systems: cardiovascular system, respiratory system, gastrointestinal system, dermatological system, extremities, musculoskeletal system, nervous system and lymph nodes.

6.9. CONCURRENT MEDICAL CONDITIONS

Concurrent medical conditions are ongoing conditions or diseases that are present at time of enrolment. This will include comorbidies such as diabetes, hypertension, chronic lung disease, cardiovascular disease and clinically significant abnormalities observed in laboratory tests, or physical examination, as judged by the principal investigator (or sub-investigator).

6.10. ENDPOINTS ANALYSIS

These listed endpoints are below (from section 3.1):

- Geometric mean concentration of anti-Spike IgG (total) in patients with cirrhosis (combined CP-A, CP-B and CP-C) with healthy participants at week 7 following second vaccine dose.
- Geometric mean concentration of anti-Spike IgM and IgG (total) and anti-RBD IgM and IgG (total) from the reference/vaccine strain at week 7 and week 30-34 following SARS-CoV-2 vaccination between all subgroups (between-group comparisons).
- Mean concentration of IgM and IgG induced by the reference strain vaccine directed against Spike from other human coronaviruses (cross-reactivity)
- Percentage neutralisation (inhibition of ACE2:RBD binding) as a surrogate index of protection induced by the reference strain vaccine against the reference viral strain
- Percentage neutralisation (inhibition of ACE2:RBD binding) as a surrogate index of protection induced by the reference strain vaccine achieved against viral variants other than the reference strain (cross-protection)
- Relationship between antibody titres against reference Spike or reference RBD (expressed as total IgG or IgM antibody quantifications or IgG/IgM ratios), neutralisation against the reference strain, and percentage neutralisation against reference Spike/RBD or viral variants other than the reference strain
- Immunogenicity of third dose booster vaccination (increment in anti-Spike IgM and IgG and anti-RBD IgM and IgG as pre/post analysis) in all subgroups.
- Correlation of geometric mean concentrations of anti-Spike IgM and IgG and anti-RBD IgM and IgG with incidence of Covid-19 cases in each subgroup (needs to be adjusted for local incidence of Covid-19 cases at each timepoint and geographic region)
- Correlation of neutralisation against Spike or RBD from different viral variants with incidence of Covid-19 cases and/or hospital admission in each subgroup, stratified by infecting viral variant (needs to be adjusted for local incidence of Covid-19 cases at each timepoint and geographic region)Predictors of vaccine immunogenicity as univariable and multivarible analysis of all variables.
- Predictors of liver-related hospitalisations, liver transplantation and mortality as univariable and multivariable analyses of all variables.

Student t-test or analysis of variance will be used to compare normally-distributed variables, normalised (log-transformed) concentrations of anti-Spike/RBD IgM and IgG, log-transformed IgG/IgM ratios (as index of antibody maturation, which we know would involve the presence of vaccine-specific or virus-specific T-cells), or logit-transformed neutralisation percentages at week 7 and week 30-34 following SARS-CoV-2 vaccination between groups.

Paired Student t-test or repeated-measures ANOVA (or Mixed Linear models or non-parametric tests, depending on the data at hand) at week 7 and week 30-34 will be used to evaluate the increment in anti-Spike IgM and IgG and anti-RBD IgM and IgG after the third vaccine dose.

Spearman’s rank correlations will be used to assess the relationship between continuous variables and degree of response determined by antibody levels. Linear relationships will be assessed by Pearson’s product-moment correlation.

Univariable (univariable and multivariable) logistic regression will be carried out to study independent predictors of antibody response. Covariables showing a clinical and statistical significance or participating as a confounding factor for the variable of interest will be included in the final models.

GLM uni-/multi-variable models other than logistic regression may be used to investigate relationships and linear predictivity for non-normally distributed variables.

The proportional-hazard model for competing risks proposed by Fine and Gray will be used to identify differences between groups of study and development of covid-19 infection, liver-related hospitalisation or mortality, caused by Covid-19 and liver disease. Independent covariates will be included in the models when show statistical significance or confusion. This model will be chosen to account liver transplantation or the different causes of mortality as events competing with the primary event in each case.

Multi-omic data data (e.g. whole blood transcriptome) will be analysed by Student's t-test or ANOVA to identify differentially expressed variables (e.g. genes) according to Ab response. R values will be used to correlate multi-omic data (e.g. RNA microarray) with Ab levels and other clinical data (e.g. acute decompensation of cirrhosis). Paired-t-test comparisons will be performed at 7 and 34-weeks following vaccination.

Multivariable strategies will be employed to investigate how immunological, serological and clinical covariates link together and whether multivariable profiles may be used to predict vaccine response and efficacy.

For the purpose of statistical modelling, a 70:30 or 80:20 split of the cohort at hand (with equal proportional group representation) may be applied to obtaining into a training set and a validation set to build and test the models respectively.

6.11. CONCOMITANT MEDICATIONS

Detailed information (drug name, duration and daily dose) will be obtained on all administered medications at the time of enrolment.

6.12. CLINICAL LABORATORY TESTS

Laboratory parameters will also be collected according to the schedule shown in figure 1. Additionally, the investigator will collect data on haematology (full blood count and coagulation) and biochemistry (liver and renal) profiles at the following time points if available: baseline (-3 months), week 7 (±21 days), week 34 (±28 days) and booster dose + 7 weeks (±28 days).

6.13. LEVEL OF SIGNIFICANCE AND MULTIPLE COMPARISONS

In the primary analysis, hypothesis testing to compare patients with cirrhosis (combined CP-A, CP-B and CP-C) with healthy participants at week 7 will be performed at the two-sided 0.05 significance level.

Data will be reviewed and corrected for multiple hypothesis testing if necessary.

6.14. ADJUSTMENT FOR COVARIATES

Age and sex will be adjusted for.

6.15. HANDLING OF DROPOUTS AND MISSING DATA

In general, missing data will not be imputed. However, if we can consider missing values as random, a multiple imputation based on a mixed model including all potential factors for multivariable analysis will be carried out.

6.16. MULTICENTER STUDIES

Data comes from several hospitals around Europe. No multicenter evaluation was initially described in the Study Protocol, so it is not planned to use any method to link results to the hospital involved. If

appropriate and feasible (if centers have enough patients to perform quantitative analysis), individual center results should be presented. Any extreme or opposite results among centers should be noted and discussed. If there are significant differences between centres, multivariable frailty competing risk analyses could be performed.

6.17. EXAMINATION OF SUBGROUPS

Planned subgroup analyses include:

- Liver transplant (short vs long)
- Cirrhosis (compensated, CP-A, vs decompensated, CP-B/C)
- Autoimmune liver disease

If multivariable clustering analyses will highlight the presence of unexpected latent profiles of correlated variables or latent clusters of co-varying subjects, unplanned subgroup analyses may include retrospective analysis of clinical data based on differential clustering profiles.

7. LIST OF OUTPUTS

Numbers presented below refer to the corresponding appendix number in ICH E3 (e.g., number 14.3 refers to the safety data tables in ICH E3).

7.1. TABLES AND GRAPHS TO BE PRODUCED FOR THE CLINICAL STUDY REPORT

PATIENT CHARACTERISTICS

Number	Type	Content
14.1.1	Table	Patient disposition in analysis sets and reason for exclusion
14.1.2	Table	Patient discontinuation
14.1.3	Table	Number of patients by visit
14.1.4	Table	Demographics and anthropometric characteristics, vital signs and disease characteristics at baseline
14.1.5	Table	Medical history
14.1.6	Table	Study disease history and substance use (tobacco, alcohol)
14.1.7	Table	Physical examination

PRIMARY ENDPOINTS

Number	Type	Content
14.2.1	Table	Geometric mean concentration of anti-Spike IgG (total) in patients with cirrhosis (combined CP-A, CP-B and CP-C) with healthy participants at week 7 following second vaccine dose
14.2.2	Table	Geometric mean concentration of anti-Spike IgG (total) in patients with cirrhosis (combined CP-A, CP-B and CP-C) with healthy participants at week 7 following second vaccine dose. Model with covariates

Number	Type	Content
14.3.1	Table	Geometric mean concentration of anti-Spike IgM and IgG (total) and anti-RBD IgM and IgG (total) at week 7 and week 30-34 following SARS-CoV-2 vaccination between all subgroups (between-group comparisons).
14.3.2	Figure	Modelling of antibody (anti-Spike IgM and IgG and anti-RBD IgM and IgG) decay over time in individuals sampled at multiple time points
14.3.3	Table	Immunogenicity of third dose booster vaccination (increment in anti-Spike IgM and IgG and anti-RBD IgM and IgG as pre/post analysis) in all subgroups.
14.3.4	Table	Correlation of geometric mean concentrations of anti-Spike IgM and IgG and anti-RBD IgM and IgG with incidence of Covid-19 cases and/or hospitalisations in each subgroup (may need to be adjusted for local incidence of Covid-19 cases at each timepoint and geographic region)

Number	Type	Content
14.3.5	Table	Geometric mean concentration of anti-Spike IgM and IgG (total) and anti-RBD IgM and IgG (total) at week 7 and week 30-34 following SARS-CoV-2 vaccination between all subgroups (between-group comparisons).
14.3.6	Figure	Geometric mean IgG and IgM levels against Spike from other human coronaviruses
14.3.7	Figure	Geometric mean IgG/IgM ratios to Spike and RBD from the reference viral strain
14.3.8	Figure	Geometric mean IgG/IgM ratios to Spike from other human coronaviruses
14.3.9	Figure	Mean neutralisation percentages (and related mean neutralisation odds) against Spike and RBD from the reference strain
14.3.10	Figure	Mean neutralisation percentages (and related mean neutralisation odds) against Spike from viral variants other than the reference strain
14.3.11	Table	Predictors of vaccine immunogenicity as univariable analysis.
14.3.12	Table	Predictors of vaccine immunogenicity as multivariable analysis of all variables.
14.3.13	Table	Predictors of hospitalisations as univariable analysis.
14.3.14	Table	Predictors of hospitalisations as multivariable analysis of all variables.
14.3.15	Table	Predictors of acute decompensation of liver disease as univariable analysis.

14.3.16	Table	Predictors of acute decompensation of liver disease as multivariable analysis of all variables.
14.3.17	Table	Predictors of liver transplantation as univariable analysis.
14.3.18	Table	Predictors of liver transplantation as multivariable analysis of all variables.
14.3.19	Table	Predictors of mortality as univariable analysis.
14.3.20	Table	Predictors of mortality as multivariable analysis of all variables.

SAFETY

Number	Type	Content
14.3.1.1	Table	Summary of local and systemic reactions (AE)
14.3.4.1	Table	Clinical laboratory: Haematology
14.3.4.2	Table	Clinical laboratory: Biochemistry
14.3.5	Table	Clinical events
14.3.6	Table	Meld and Child Pugh Scores
14.3.7	Table	Concomitant medication and procedures

7.2. LISTINGS OF INDIVIDUAL PATIENT DATA AND OTHER INFORMATION TO BE PRODUCED FOR THE CLINICAL STUDY REPORT

Number	Content
16.2.1.1	Patient disposition
16.2.1.2	Study termination
16.2.1.3	Visit dates
16.2.4.1	Demographics and other characteristics
16.2.4.2	Medical history and study disease history
16.2.4.3	Inclusion criteria not met, and exclusion criteria met
16.2.6.1	Titre of anti-Spike IgG (total)
16.2.6.2	Titre of anti-RBD IgM
16.2.6.3	Meld and Child Pugh Scores
16.2.7.1	Adverse Effects
16.2.7.2	Hospital Stays
16.2.8.1	Listing of individual laboratory measurements by patient

16.2.9.1 Concomitant medication and procedures

16.2.10.1 Physical examination

8. REFERENCES

(1) McCashland TM et al. Pneumococcal vaccine response in cirrhosis and liver transplantation. *Journal of Infectious Diseases* 2000;181:757–60.
(2) Ramasamy MN et al. Safety and immunogenicity of ChAdOx1 nCoV-19 vaccine administered in a prime-boost regimen in young and old adults (COV002): a single-blind, randomised, controlled, phase 2/3 trial. *Lancet*, November 18 2020, doi: 10.1016/S0140-6736(20)32466-1.
(3) US Food and Drug Administration. Guidance for industry: toxicity grading scale for healthy adult and adolescent volunteers enrolled in preventive vaccine clinical trials. Rockville, MD: Center for Biologics Evaluation and Research; September 2007.

APPENDICES

Appendix 1: Definitions of chronic illness (for identification of healthy participants)

Healthy participants will be recruited according to the absence of uncontrolled and severe cardiac, respiratory, liver, renal or endocrine disease, according to the discretion of the PI or sub-I. Definitions of severe disease are provided below:

Severe heart disease

- Unstable angina or recent myocardial infarction (NSTEMI or STEMI)
- Congenital heart disease requiring lifelong follow-up
- Congestive heart failure meeting criteria ≥ New York Heart Association Class III or Class C

Severe respiratory disease:

- Chronic obstructive pulmonary disease (COPD) requiring long-term oxygen therapy
- Interstitial lung fibrosis
- Asthma with previous severe or life-threatening attack or requiring critical care admission

Severe liver disease

- Advanced liver fibrosis or cirrhosis

Severe renal disease

- Chronic kidney disease with requirement for dialysis

Severe endocrine disease

- Uncontrolled diabetes mellitus (symptomatic or recent HbA1c >64 mmol/L)
- Uncontrolled (symptomatic) thyroid disease
